# Detection of a High-Turnover Serotonin Circuit in the Mouse Brain Using Mass Spectrometry Imaging

**DOI:** 10.1016/j.isci.2019.09.036

**Published:** 2019-09-27

**Authors:** Eiji Sugiyama, Matteo M. Guerrini, Kurara Honda, Yuko Hattori, Manabu Abe, Patrik Källback, Per E. Andrén, Kenji F. Tanaka, Mitsutoshi Setou, Sidonia Fagarasan, Makoto Suematsu, Yuki Sugiura

**Affiliations:** 1 Department of Biochemistry, Keio University School of Medicine, 35 Shinanomachi, Shinjuku, Tokyo 160-8582, Japan; 2 Laboratory for Mucosal Immunity, Center for Integrative Medical Sciences, RIKEN Yokohama Institute, Tsurumi Ward, Suehirocho, 1 Chome-7-22, Yokohama, Kanagawa Prefecture 230-0045, Japan; 3 Department of Animal Model Development, Brain Research Institute, Niigata University, 1-757 Asahimachi-dori, Chuo-ku, Niigata 951-8585, Japan; 4 Medical Mass Spectrometry Imaging, Department of Pharmaceutical Biosciences, Uppsala University, Box 591 BMC, 75124 Uppsala, Sweden; 5 Science for Life Laboratory, National Resource for Mass Spectrometry Imaging, Uppsala University, Box 591 BMC, 75124 Uppsala, Sweden; 6 Department of Neuropsychiatry, Keio University School of Medicine, 35 Shinanomachi, Shinjuku, Tokyo 160-8582, Japan; 7 Department of Cellular and Molecular Anatomy and International Mass Imaging Center, Hamamatsu University School of Medicine, 1-20-1 Handayama, Higashi-ku, Hamamatsu, Shizuoka 431-3192, Japan

**Keywords:** Spectroscopy, Neuroscience, Systems Neuroscience

## Abstract

Monoamine neurotransmitters are released by specialized neurons regulating behavioral, motor, and cognitive functions. Although the localization of monoaminergic neurons in the brain is well known, the distribution and kinetics of monoamines remain unclear. Here, we generated a murine brain atlas of serotonin (5-HT), dopamine (DA), and norepinephrine (NE) levels using mass spectrometry imaging (MSI). We found several nuclei rich in both 5-HT and a catecholamine (DA or NE) and identified the paraventricular nucleus of the thalamus (PVT), where 5-HT and NE are co-localized. The analysis of 5-HT fluctuations in response to acute tryptophan depletion and infusion of isotope-labeled tryptophan *in vivo* revealed a close kinetic association between the raphe nuclei, PVT, and amygdala but not the other nuclei. Our findings imply the existence of a highly dynamic 5-HT-mediated raphe to PVT pathway that likely plays a role in the brain monoamine system.

## Highlights

• A murine brain atlas of monoamine (5-HT, DA, NE) levels was generated via MS imaging
• We identified several nuclei rich in both 5-HT and a catecholamine (DA or NE)
• The paraventricular nucleus of the thalamus (PVT) had high levels of 5-HT and NE
• The level of 5-HT in raphe to PVT pathway changed dynamically in response to blood Trp level

## Graphical Abstract

**Figure:**
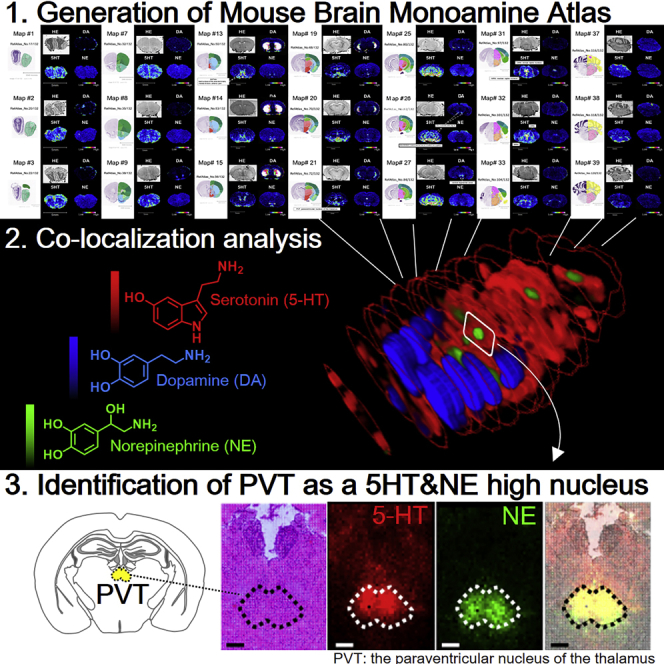

## Introduction

Monoamine neurotransmitters are a family of small molecules that include serotonin (5-hydroxytryptamine, 5-HT), dopamine (DA), and norepinephrine (NE), which are secreted by specific neuronal populations that regulate executive functions (Aston-Jones and Cohen, 2005, Lucki, 1998, Wise, 2004). Considering their essential roles in controlling goal-directed and adaptive behavior, the abundance and localization of monoamine neurotransmitters in the brain must be tightly controlled. The total amount of monoamine neurotransmitters in the brain is determined by the ratio between local synthesis and degradation, since they cannot cross the blood-brain barrier (Hardebo and Owman, 1980). The availability of monoamines to neurons is regulated by synaptic secretion (Gantz et al., 2015, Yuen et al., 2014), volume transmission (Fuxe et al., 2010), and neuronal re-uptake mediated by specific transporters (Bermingham and Blakely, 2016).

The pharmacological or genetic alterations in monoamine neurotransmitter levels can cause changes in behavior, such as impulsiveness (Schweighofer et al., 2008), hypolocomotion (Benazzouz et al., 2014), or the sleep-wake cycle (Singh et al., 2015). Numerous studies have shown that regional monoaminergic tuning directs behavior: that is, the depletion of 5-HT in the basolateral amygdala (BLA) reduces anxiety-like behaviors and weakens fear-associated memory (Johnson et al., 2015); the level of DA in the dorsal striatum is directly correlated to the adaptive changes of locomotion in a mouse model of Parkinson disease (Panigrahi et al., 2015); and reduced NE levels in the BLA increases the occurrence of anxiety-like behavior (McCall et al., 2017). Nevertheless, current data do not validate the actual variation in the local concentration or coexistence of monoamines. In addition, the monoaminergic neurons target multiple brain regions, but the kinetics and magnitude of monoaminergic signaling along different projections are not identified. At present, data regarding the intracerebral localization of 5-HT, DA, and NE remain limited and unclear owing to several long-standing technical limitations inherent to their detection. Thus, we assumed that the direct detection of monoamine levels in the brain may be a powerful and unbiased approach to identify the intensity and kinetics of monoaminergic signaling targeting different brain regions.

We used mass spectrometry imaging (MSI) (Caprioli et al., 1997), which is a technique recently developed for the analysis of the molecular composition of a planar sample with high sensitivity and specificity (Nilsson et al., 2015). Unlike other fluorescence- or radioisotope-based imaging techniques (i.e., the Falck-Hillarp method [Falck and Hillarp, 1959], immunohistochemistry [IHC], or autoradiography [ARG]), MSI does not require any probes and preparations that could affect the distribution of metabolites (i.e., pharmaceutical activation, perfusion, fixation, washing, or blocking); therefore, it is an ideal tool for mapping monoamines in the brain. Although the applicability of MSI had been limited by the low ionization efficacy of monoamines (Shariatgorji et al., 2014, Sugiura et al., 2012), this issue was recently solved with the use of on-tissue chemical derivatization, which converts the primary amino groups of monoamines into quaternary amino groups that provide high ionization efficacy (Shariatgorji et al., 2014). We used 2,4-diphenyl-pyrylium (DPP) since DPP derivatives were detected with better signal intensities than those of other compounds (Esteve et al., 2016, Shariatgorji et al., 2015). We have previously used this approach and detected changes in monoamine levels in the mouse brain (Miyajima et al., 2017, Shariatgorji et al., 2014, Sugiyama et al., 2019).

In this study, we used MSI to generate an atlas of 5-HT, DA, and NE in the whole brain of the C57BL/6J mouse. Our data revealed an unexpected accumulation of multiple monoamines, particularly 5-HT and a catecholamine (DA or NE), in several brain nuclei. Notably, quantitation of 5-HT revealed the paraventricular nucleus of the thalamus (PVT), which receives a raphe-derived dense serotonergic innervation (Migliarini et al., 2013, Muzerelle et al., 2016), as the region containing almost the second highest 5-HT level within the mouse brain. We then analyzed the dynamics of the deprivation and replenishment of 5-HT in specific brain regions following acute tryptophan depletion (ATD) or tryptophan (Trp) supplementation in the mouse diet, which are two treatments known to affect anxiety-like behavior in mice. Our results provided details about monoamine distribution in the brain and the regional differences in 5-HT metabolism and showed that MSI is a powerful system that can be used to detect monoamine fluctuations in the brain of mice subjected to a behavioral experiment.

## Results

### Generation of a Mouse Brain Atlas of 5-HT, DA, and NE

**Figure 1:**
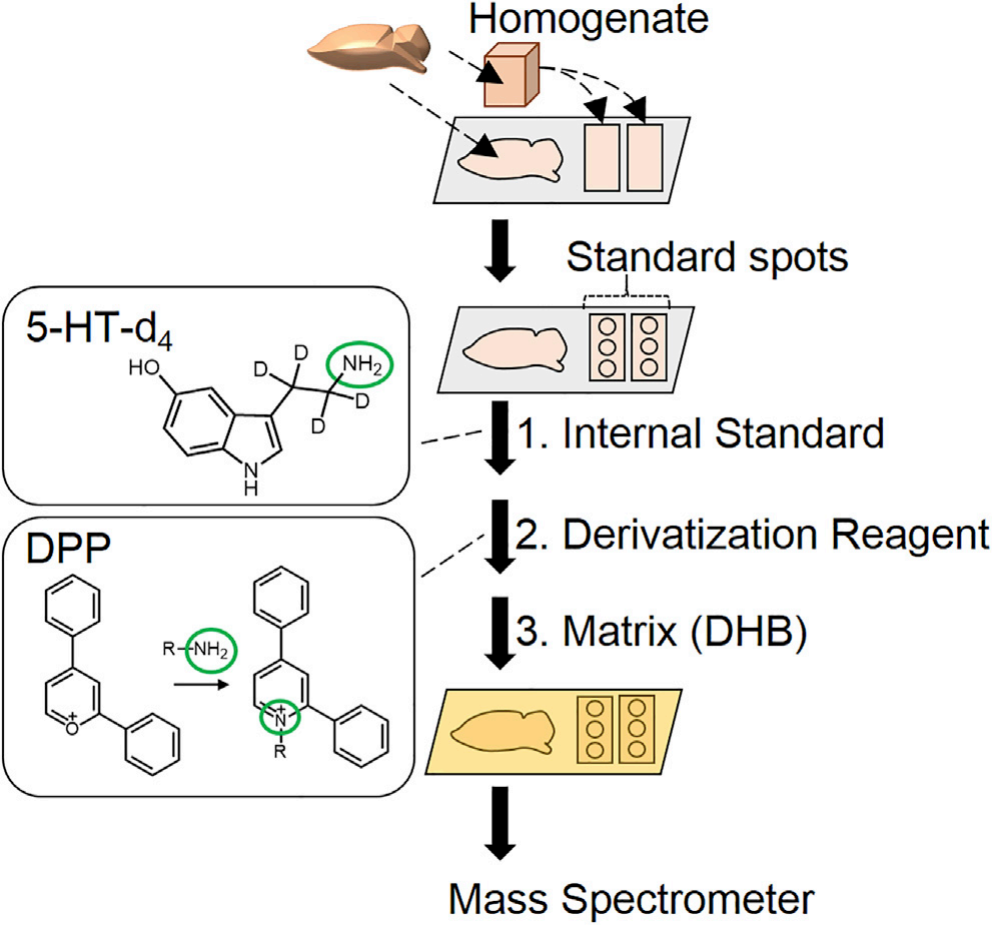
Sample Preparation for Mapping Brain Monoamines Using Mass Spectrometry Imaging Mouse brain slices and brain homogenate were disposed on conductive glass slides coated with indium-tin-oxide. A series of standard solution containing the known concentration of 5-HT was spotted onto the homogenate sections. An internal standard solution (5 μM of D_4_-5-HT in 50% methanol), derivatization solution (1.3 mg/mL of 2,4-diphenyl-pyranylium [DPP] in methanol), and matrix solution (40 mg/mL of 2,5-dihydroxybenzoic acid [DHB] in 50% methanol) were sprayed onto the sections using an automatic sprayer, airbrush, and automatic sprayer, respectively. The DPP derivatives (5-HT-DPP, NE-DPP, and DA-DPP) were detected using mass spectrometers equipped with a MALDI-ion source. See also Transparent Methods and Figure S7.

**Figure 2:**
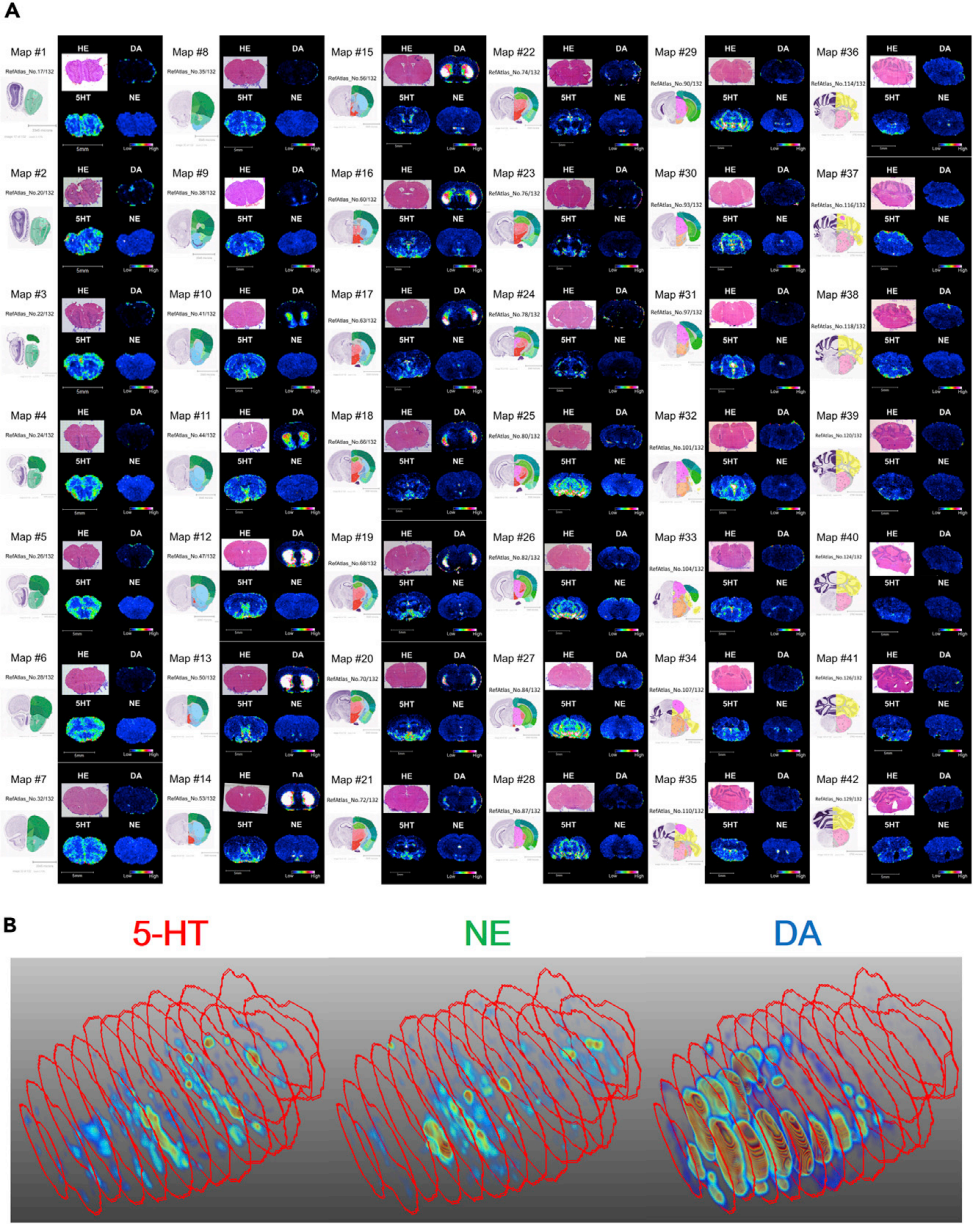
Distribution of 5-HT, NE, and DA in the Mouse Brain (A) A mouse whole brain atlas of 5-HT, NE, and DA. Hematoxylin and eosin staining and signal intensity of 5-HT, DA, and NE in 42 brain sections of an 8-week-old male C57BL/6J mouse referenced to brain maps from the Allen Brain Reference Atlases (http://atlas.brain-map.org/). Signal intensities were normalized using total ion current and the signal obtained from the slice of the homogenate. The color scales were consistent throughout the series but not between different monoamines. High-resolution images are presented in the Supplemental Information. Data were obtained using a time-of-flight (TOF) mass spectrometer. (B) Three-dimensional distribution of 5-HT, NE, and DA in the mouse brain. Graphical representation of the normalized signal intensity of 5-HT (left), NE (center), and DA (right) in 15 brain coronal sections of the brain of a C57BL/6J mouse. Red frames represented the shapes of the measured area of each section. See also Video S1. The data were obtained by the Fourier transform ion cyclotron resonance (FT-ICR) mass spectrometer.

We analyzed the coronal slices of a mouse brain prepared at an interval of 330 μm (a total of 42 sections) using MSI using an 8-weeks old C57BL/6J male mouse as a representative example (Figure 1). The sections were then stained with hematoxylin and eosin and matched to a reference atlas. The monoamines were found to be highly abundant in the expected brain nuclei, thereby validating the specificity and spatial resolution of MSI (Figure 2A, Data S1). The dorsal raphe nucleus (DRN) and the median raphe nucleus (MRN) were extremely rich in 5-HT, the substantia nigra (SN) and the ventral tegmental area (VTA) had high DA levels, and an extremely high concentration of NE was observed in the locus coeruleus (LC) (Table 1). The concentration of DA in the striatum and nucleus accumbens, which are regions targeted by extensive dopaminergic innervation, was higher than that in the nuclei in which DA is produced (SN or VTA). Meanwhile, the concentration of 5-HT was highest in the DRN, which is the largest raphe nucleus (Table 1, Figure 2A). Such tendency in NE was not clearly elucidated. In addition, we detected other monoamine-rich nuclei. DA was abundant in the basolateral and basomedial amygdala (BLA/BMA) and 5-HT was rich in the SN, VTA, BLA/BMA, PVT, reuniens nucleus and rhomboid nucleus (ReN/RhN), caudal part of the hippocampus (cHip), and midbrain raphe nucleus (MiRN). NE was abundant in the PVT, posteroventral part of the bed nucleus of the stria terminalis (pBNST), ventral part of the periaqueductal gray (vPAG), posterior hypothalamic nucleus (PHN), and DRN (Table 1). To the best of our knowledge, the functions of 5-HT or NE in the PVT and ReN/RhN are not known (Cassel et al., 2013, Do Monte et al., 2016). Next, we generated tridimensional distributions of the three monoamines using a set of 15 coronal sections from another C57BL/6J male mouse of the same age. 5-HT, DA, and NE were widely distributed through the brain nuclei and forebrain, mainly with a non-overlapping pattern (Figure 2B and Video S1). The monoamine concentrations were high in the whole limbic system but significantly lower in the cortex, indicating their different qualitative roles in different regions. Moreover, monoamine neurotransmitters presented a non-overlapping distribution pattern, except in few regions, thereby indicating a mutually exclusive regulation in most brain nuclei.

**Table 1:** Major Brain Regions Enriched with Monoamines

Brain Regions	5-HT	DA	NE
DRN	++++		++
SN	+++	++	
VTA	+++	++	
BLA/BMA	+++	++	
PVT	+++		++++
MRN	+++		
vPAG	++		+++
ReN/RhN	++		
cHip	++		
MiRN	++		
aBNST	+	+	
CP		+++++	
NAc		++++	
LC			++++
pBNST			++++
PHN			+++

DRN, dorsal raphe nucleus; SN, substantia nigra; VTA, ventral tegmental area; BLA/BMA, basolateral amygdala and basomedial amygdala; PVT, paraventricular nucleus of the thalamus; MRN, median raphe nucleus; vPAG, ventral part of the periaqueductal gray; ReN/RhN, reuniens nucleus and rhomboid nucleus; cHip, caudal part of the hippocampus; MiRN, midbrain raphe nucleus; aBNST, anterior division, anteromedial area, and anterior part of the bed nucleus of the stria terminalis; CP, caudate-putamen; NAc, nucleus accumbens; LC, locus coeruleus; pBNST, anterior division, anteromedial area, and posteroventral part of the bed nucleus of the stria terminalis; PHN, posterior hypothalamic nucleus.

Video S1. The 3D Distribution of Serotonin, Dopamine, and Norepinephrine in the Brain of an 8-Weeks Old C57BL/6J Male Mouse, Related to Figure 2B

### Co-localization of 5-HT and Catecholamines

**Figure 3:**
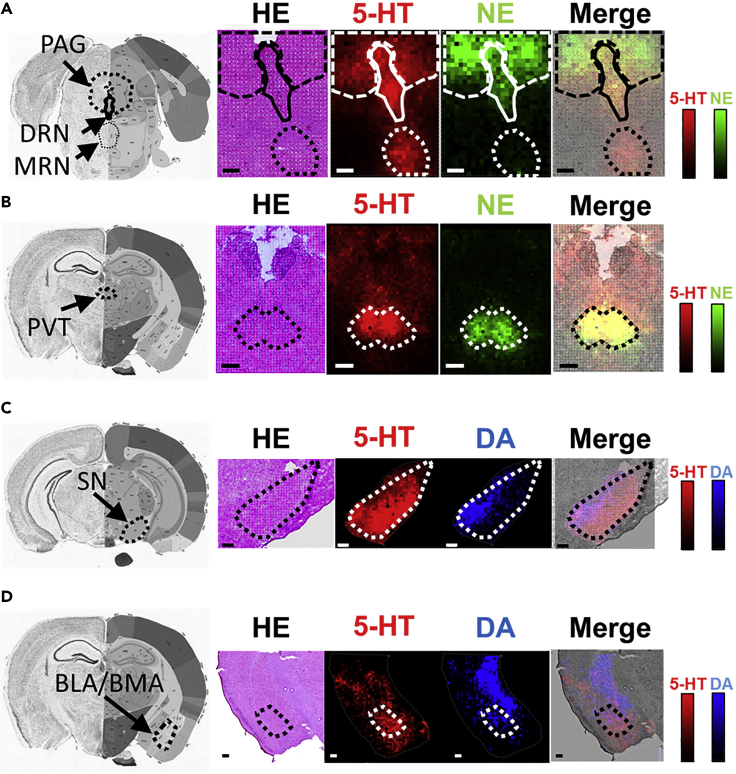
Representative Images Showing the Co-localization of 5-HT and Catecholamine in the Brain Nuclei Reference coronal brain atlas, hematoxylin and eosin staining findings, graphical representation of 5-HT levels, catecholamine (NE or DA) levels, and merged 5-HT and catecholamine are shown from left to right. (A) Ventral part of the periaqueductal gray (PAG) and dorsal raphe nucleus (DRN). (B) Paraventricular nucleus of the thalamus (PVT). (C and D) (C) Substantia nigra (SN) and (D) basolateral/basomedial amygdala (BLA/BMA). Scale bars, 200 μm. Data were obtained using the FT-ICR mass spectrometer.

The simultaneous imaging of the three monoamines facilitated the identification of few areas of monoamine co-localization. We then focused our analysis on these regions. Monoamines modulate neuronal activity based on other neurotransmitters, and they can affect other functions (Hamon and Blier, 2013, Niederkofler et al., 2015, Pudovkina et al., 2002). For example, 5-HT acts on dopaminergic neurons in the SN, thereby suppressing the production of DA (Cobb and Abercrombie, 2003, Patel et al., 2004). Therefore, the presence of multiple monoamines in some regions of the brain indicates that they might be regulatory sites in which different monoaminergic systems directly interact. We detected the co-localization of 5-HT and NE in the DRN, but not in the MRN, and in the vPAG, akin to the PVT (Figures 3A and 3B). 5-HT and DA were found in the SN (Figure 3C), BLA/BMA (Figure 3D), and VTA (Figure 2A, Supplemental Information). Thus, we identified multiple sites where 5-HT co-localized with either DA or NE. In contrast, none of the nuclei were rich in both NE and DA. These results indicated that 5-HT has a unique potential of integrating to the three monoaminergic signaling networks because it overlaps with the other two systems in several major brain nuclei, including the SN.

### Quantitative Comparison Identifies PVT as a 5-HT-Rich Nucleus

**Figure 4:**
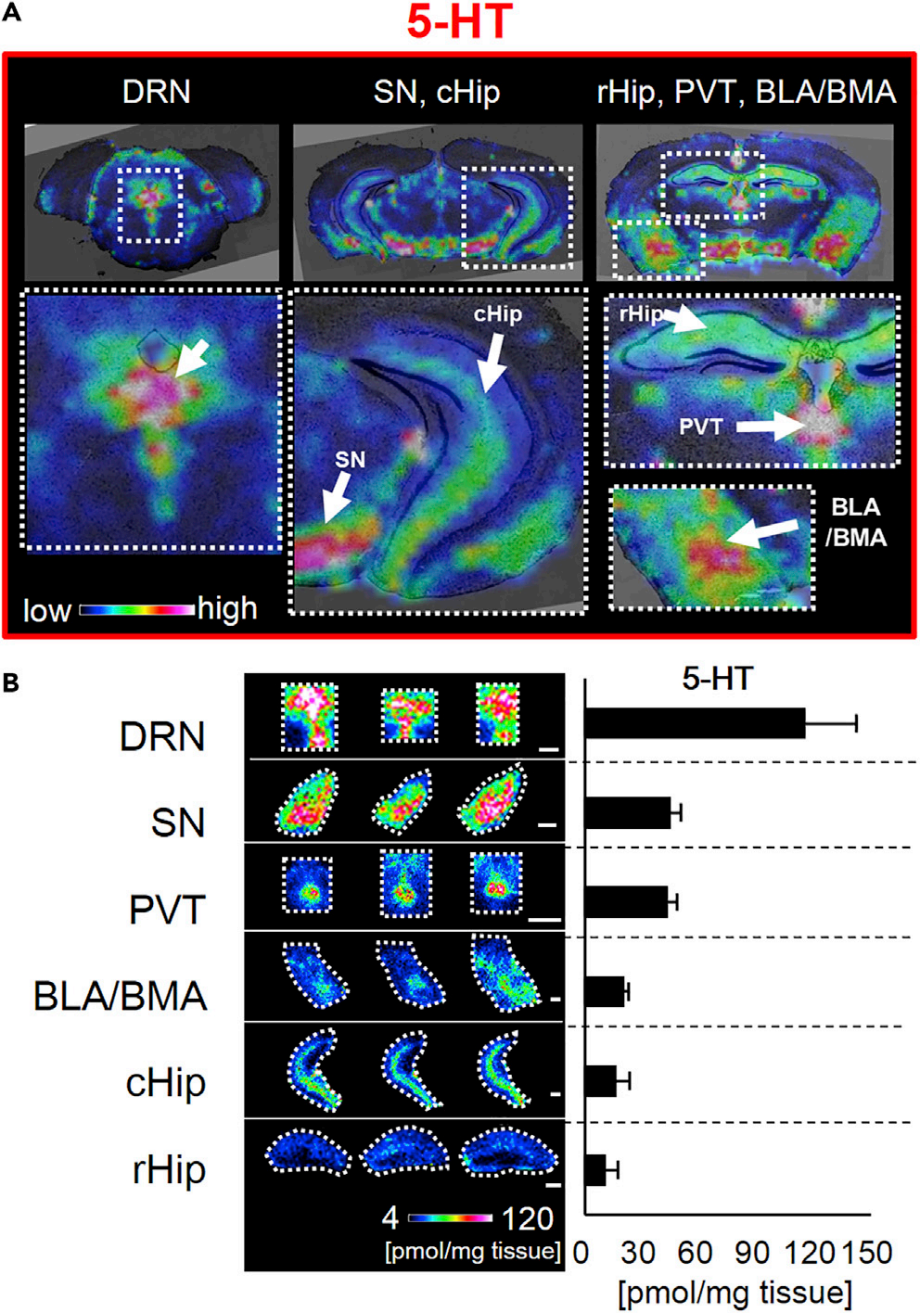
Quantitative Comparison of 5-HT in Selected Brain Nuclei (A) Representative ion images showing the quantities of 5-HT-DPP in selected brain nuclei. The color scales were adjusted for each section. DRN, dorsal raphe nucleus; SN, substantia nigra; cHip, caudal part of the hippocampus; rHip, rostral part of the hippocampus; PVT, paraventricular nucleus of the thalamus; BLA/BMA, basolateral/basomedial amygdala. (B) Left. Ion images showing 5-HT in the selected brain regions of three mice. Scale bar, 500 μm. The color scale depicting 5-HT quantitation is shown in the figure panel. Right. Averaged quantities of 5-HT per tissue weight in the regions shown in the panel. Data were presented as mean ± SD; mice, n = 3. Data were obtained using the FT-ICR mass spectrometer.

Our study aimed to quantify the levels of 5-HT in several 5-HT-rich brain nuclei. We selected three coronal sections and mapped the concentration of 5-HT in six regions of interest: the DRN, SN, PVT, BLA/BMA, and hippocampus, divided in its caudal (cHip) and rostral parts (rHip) (Figure 4A). Among these regions, the somata of the serotonergic neurons can be found only in the DRN, where the highest concentration of 5-HT was observed. The average quantities of 5-HT in the SN and PVT were unexpectedly high, which were approximately 40% of the DRN value (Figure 4B). Therefore, the SN and PVT are the main brain regions targeted by serotonergic signaling in the steady state. The average quantities of 5-HT in the BLA/BMA, cHip, and rHip were 20%, 16%, and 10% of that detected in the DRN, respectively. Although minimal, the difference between cHip and rHip was statistically significant.

### Classification of Brain Nuclei Based on Changes in 5-HT Level in the ATD Model

**Figure 5:**
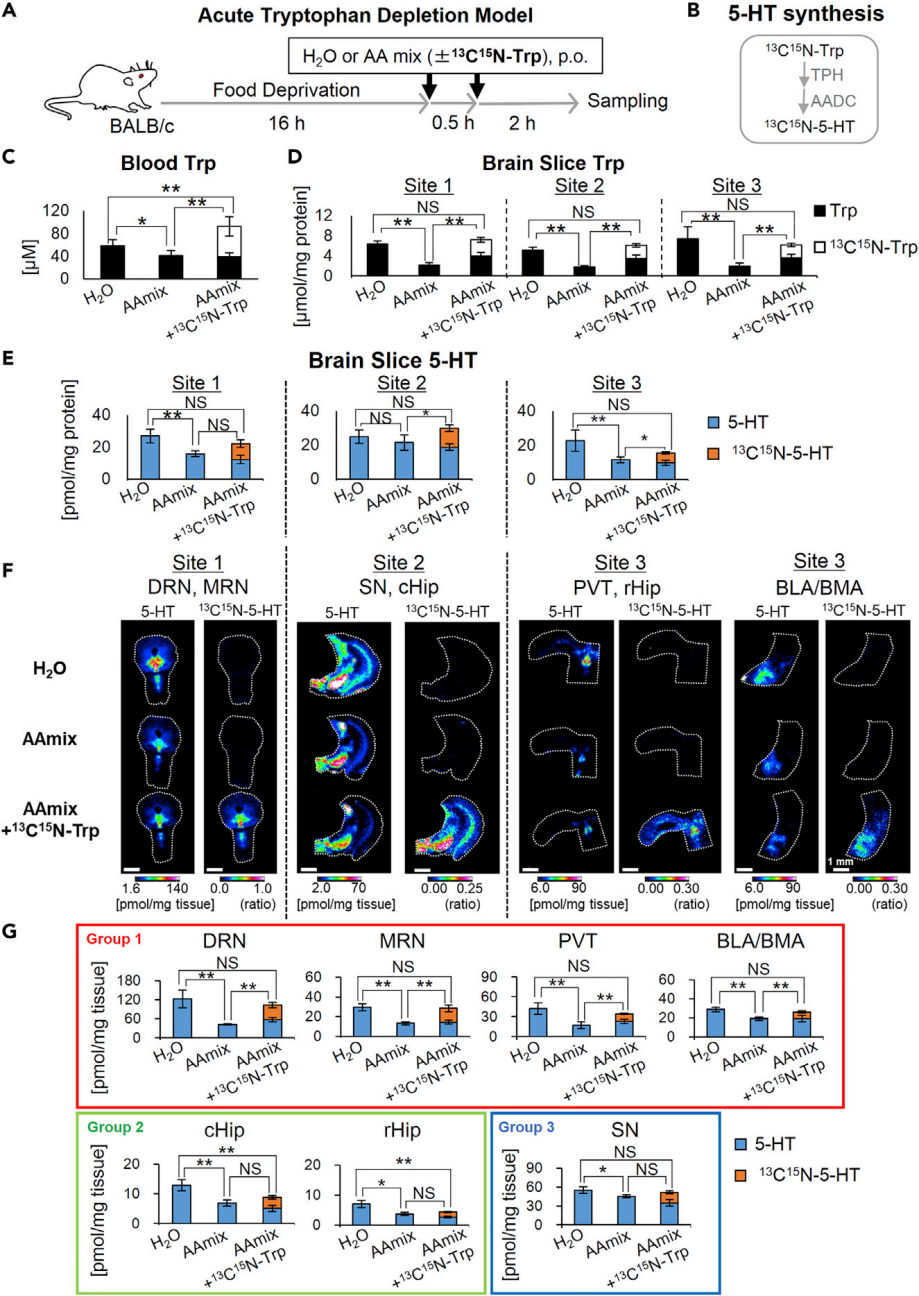
Classification of Brain Nuclei Based on 5-HT Turnover Using an ATD Model (A) Schematics of the ATD experiment. After 16 h of food deprivation, the BALB/c mice were gavaged twice with H_2_O, amino acid mixture without tryptophan (AAmix), or AAmix supplemented with labeled tryptophan (AAmix + ^13^C^15^N-Trp) at an interval of 30 min. (B) Synthetic pathway of ^13^C^15^N-5-HT from ^13^C^15^N-Trp. TPH, tryptophan hydroxylase; AADC, Aromatic L-amino acid decarboxylase. (C) Concentration of tryptophan (Trp) and ^13^C^15^N-Trp in the whole blood (mice gavaged with H_2_O, AAmix, and AAmix + ^13^C^15^N-Trp: n = 9, 11, and 10, respectively). (D) Normalized amounts of Trp and ^13^C^15^N-Trp in the extracts of whole brain slices at three sites (mice per group: n = 5, 5, and 5, respectively). (E) Normalized amounts of 5-HT (blue) and labeled ^13^C^15^N-5-HT (orange, synthesized from ^13^C^15^N-Trp) in three whole coronal brain sections. Site 1 includes DRN and MRN; site 2 includes SN and cHip; site 3 includes PVT, rHip, and BLA/BMA (mice gavaged with H_2_O, AAmix, and AAmix+^13^C^15^N-Trp: n = 5, 5, and 5, respectively). (F) Representative ion images of 5-HT-DPP and ^13^C^15^N-5-HT-DPP in the sections consecutive to the ones analyzed in (C). Note that the measured areas do not cover the whole sections. (G) Classification of the seven nuclei based on 5-HT turnover. The bars show normalized amounts of 5-HT (blue) and labeled ^13^C^15^N-5-HT (orange, synthetized from ^13^C^15^N-Trp) in the indicated brain nuclei (mice for BLA/BMA, n = 4 and mice for other nuclei per group, n = 5). Data in (C), (D), (E), and (G) were presented as mean ± SD. *, p < 0.05; **, p < 0.005 (Tukey's HSD test). DRN, dorsal raphe nucleus; MRN, median raphe nucleus; PVT, paraventricular nucleus of the thalamus; BLA/BMA, basolateral/basomedial amygdala; cHip, caudal part of the hippocampus; rHip, rostral part of the hippocampus; SN, substantia nigra. Data in (F) and (G) were obtained using the FT-ICR mass spectrometer.

The serotonergic axon terminals targeting SN and PVT belong to different subsets of raphe neurons (Kiyasova et al., 2011, Muzerelle et al., 2016). Therefore, we hypothesized that the metabolic regulation of 5-HT differs among these nuclei. To test this possibility, we used the ATD model in which the mice received two oral administrations of a mixture of amino acids devoid of Trp (hereafter AAmix). A lower Trp level in the blood and brain was observed in the ATD model than in the water gavage group (Biskup et al., 2012) since essential amino acids in the Trp-free diet increases protein synthesis (Gessa et al., 1974, Moja et al., 1991) and large neutral amino acids compete with Trp in entering into the brain across the blood-brain barrier (Gessa et al., 1974). In addition, the decrease in Trp concentration in the blood affects anxiety-related behaviors (Biskup et al., 2012, Crockett et al., 2012, Young, 2013). Thus, ATD was expected to reduce the 5-HT levels in the brain nuclei, and Trp supplementation would increase them. To test this hypothesis, we used BALB/c mice, which have demonstrated decreased anxiety behavior on the ATD protocol (Biskup et al., 2012). MSI techniques were used to visualize the fluctuation of 5-HT in the SN and PVT after ATD. We then supplemented the mice with ^13^C^15^N-labeled Trp (^13^C^15^N-Trp) to monitor the *de novo* synthesis of mass-labeled 5-HT (^13^C^15^N-5HT) (Figures 5A and 5B). The concentration of total Trp (the sum of Trp and ^13^C^15^N-Trp) in the blood of mice receiving AAmix was lower by 70% than that of the control group receiving water gavages, corresponding to 44% of the group receiving the AAmix with ^13^C^15^N-Trp (Figure 5C). We evaluated the total amounts of Trp and 5-HT in the extracts of brain coronal slices at three sites: site 1, including DRN and MRN; site 2, including SN and rHip; and site 3, including PVT, cHip, and BLA/BMA. The ATD treatment substantially decreased the total concentration of Trp to 34%, 36%, and 27% of the control levels in sites 1, 2, and 3, respectively, and the co-administration of AAmix and ^13^C^15^N-Trp restored the Trp levels to control levels (Figure 5D). Smaller variations in 5-HT levels were observed: the 5-HT levels significantly decreased to 59% and 51% in sites 1 and 3, respectively, compared with control levels, but not in site 2. The co-administration of AAmix and ^13^C^15^N-Trp significantly increased the total 5-HT levels in sites 2 and 3, but not in site 1, compared with the administration of AAmix alone (Figure 5E).

We then analyzed the 5-HT metabolism in seven 5-HT-rich nuclei (DRN, MRN, SN, PVT, rHip, cHip, and BLA/BMA). We readily detected both unlabeled and newly synthesized 5-HT (^13^C^15^N-5HT) in all the nuclei (Figures 5F and S1). The 5-HT level in the PVT was significantly reduced by ATD (AAmix group versus water gavage group, Figure S2, left panel) and was increased with the supplementation of ^13^C^15^N-Trp, a kinetic similar to DRN and MRN (Figure S2, right panel). We classified the seven nuclei into three groups based on their 5-HT metabolism (Figure 5G). In the first group, which included DRN, MRN, PVT, and BLA/BMA, the total 5-HT level decreased in response to ATD, but the levels increased to control level with the addition of ^13^C^15^N-Trp to the AAmix (Figure 5G, red rectangle). In the second group, which comprised cHip and rHip, ATD led to a significant decrease in total 5-HT levels, but the levels did not increase with the addition of ^13^C^15^N-Trp to the AAmix (Figure 5G, green rounded rectangle). In the third group, which consisted of SN alone, the total 5-HT level was only marginally affected by the depletion and repletion of Trp (Figure 5G, blue rounded rectangle). These findings showed that PVT belongs to a group of nuclei metabolically comparative with the DRN, MRN, and BLA/BMA, indicating that its function is modulated in a similar time frame and that it may play a similar role in regulating anxiety-like behavior. The metabolism of 5-HT differed between the hippocampus and SN, which is consistent with their limited relevance to anxiety-like behavior. We examined tryptophan hydroxylase 1 (TPH1)-deficient mice that cannot synthesize 5-HT in the peripheral tissues and pineal gland (Cote et al., 2003, Patel et al., 2004). In these mice, the pineal gland and the lumen of the blood vessels, but not DRN and PVT, showed a significant reduction in 5-HT levels (Figure S3). The result supports the concept that the function of PVT is modulated by serotonergic neurons that synthesize 5-HT in a TPH1-independent fashion via TPH2 (Mosienko et al., 2012, Patel et al., 2004).

To obtain more insights about the fate of 5-HT newly synthesized from labeled Trp in the blood, we compared the ^13^C^15^N-5-HT levels over time in the DRN, MRN, PVT, rHip, and SN after the oral administration of AAmix plus ^13^C^15^N-Trp (Figure S4A). In the DRN and MRN, ^13^C^15^N-5-HT peaked at 45–90 min and then rapidly decreased. In other regions, the ^13^C^15^N-5-HT levels remained relatively low and reached their maximum levels at 150–300 min (Figure S4B). In addition, the ^13^C^15^N-5-HT level in the rHip was lower than that in the PVT and SN during the entire experimental time course (600 min) (Figure S4B).

**Figure 6:**
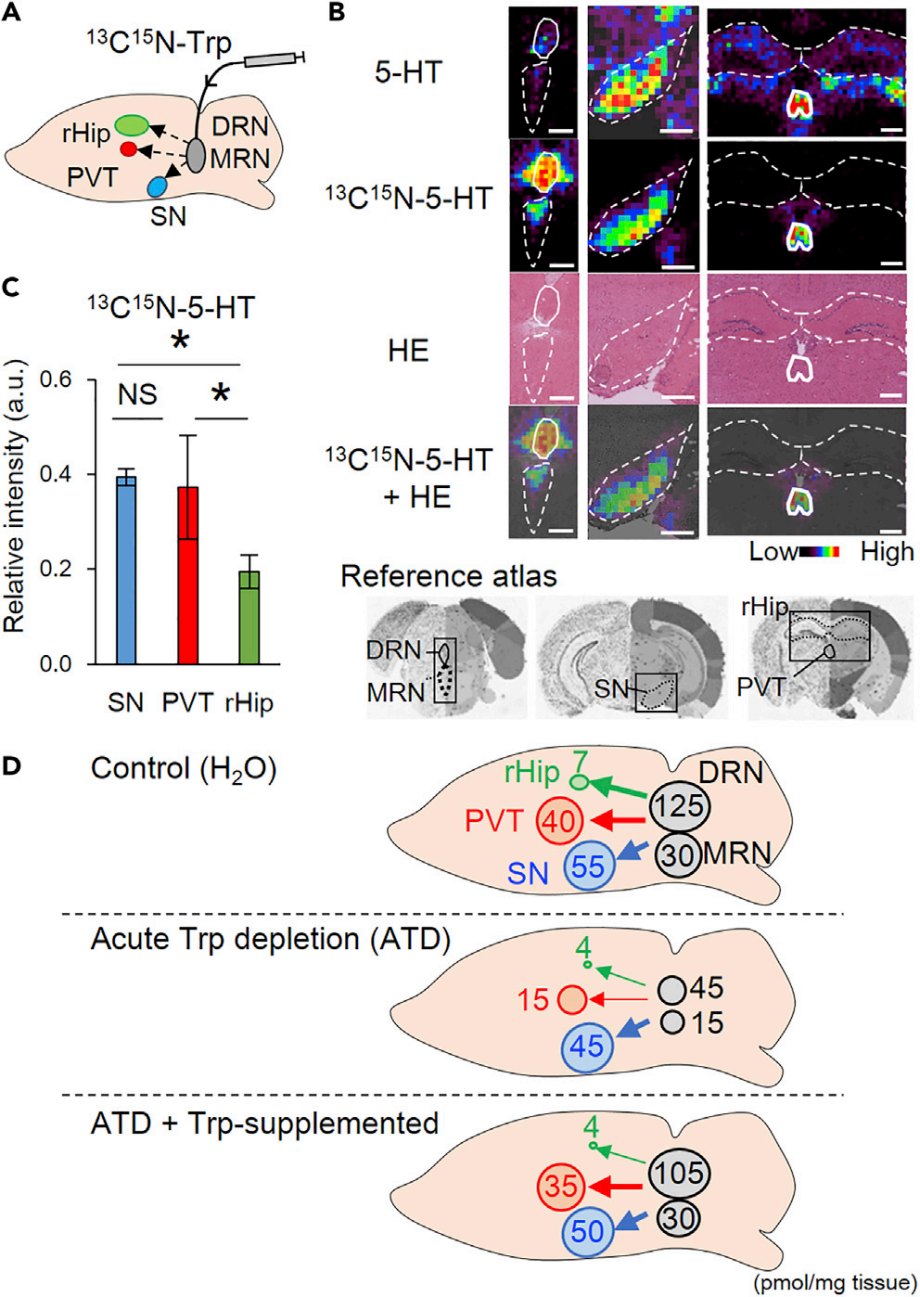
Distribution of the Newly Synthesized 5-HT Provided from the DRN and MRN (A) Schematic diagram of the experimental procedure. Continuous infusion of ^13^C^15^N-Trp to the DRN and MRN lasted for 2.5 h. (B) From the top line. Levels of 5-HT and ^13^C^15^N-5-HT, H&E staining of the same sections performed after measurement, and merged images of the ^13^C^15^N-5-HT and the H&E stain. The reference coronal brain atlas is shown at the bottom. Scale bar, 500 μm. (C) Comparison of the relative abundance of ^13^C^15^N-5-HT between the indicated regions. SN, substantia nigra; PVT, paraventricular nucleus of the thalamus; rHip, rostral hippocampus. Data were presented as mean ± SD (n = 3 mice), *, p < 0.05 (Tukey's HSD test). (D) A model of the pathway-specific adjustment of 5-HT delivery. The arrows show the levels of newly synthetized 5-HT irradiating along the serotonergic neurons from the DRN and MRN. Thick arrows represent a preferential supply, and thin arrows low priority supply. The size of the circle at each nucleus showed the level of pooled 5HT based on Figure 3G. The relative distribution in the control, ATD model, and Trp-supplemented groups are shown in the top, center, and bottom, respectively. Data were obtained using the linear ion trap mass spectrometer.

In the ATD experimental setting, the synthesis of 5-HT in the brain depends on both the rate of Trp import across the blood-brain barrier and the rate of its enzymatic conversion into 5-HT. To provide a more direct evidence, we administrated ^13^C^15^N-Trp into the DRN and MRN (Figure 6A). Two and a half hours later, most 5-HT in the DRN and MRN were newly synthesized as ^13^C^15^N-5-HT (Figure 6B). At the same time point, we detected high levels of ^13^C^15^N-5-HT in the PVT and SN but not in the rHip (Figures 6B, 6C, and S5). These results showed that the serotonergic neurons in the DRN and MRN prioritize the delivery of 5-HT to the PVT and SN rather than the rHip.

## Discussion

Herein, we report a new mouse whole brain atlas of 5-HT, DA, and NE using MSI. Our data represent an entire map of physiological monoamine level in the mouse brain, thereby opening the venue to more precise studies of their functions. Currently, most data about monoamine distributions in the brain are based on the gene expression of the key neuronal synthetic enzymes (Nagatsu, 1991, Patel et al., 2004): TPH for 5-HT, tyrosine hydroxylase for DA, and dopamine beta hydroxylase for NE. Even if a direct correlation exists between metabolic key enzymes and the production of their respective monoamines, we must consider that the availability of monoamines in the brain is affected by the catabolic activity of monoamine oxidases A and B (Shih et al., 1999) or catechol-O-methyltransferase (Gogos et al., 1998) and even further by the reuptake transporters (Bermingham and Blakely, 2016) recycling the secreted monoamines from extracellular sites.

Direct mapping of the brain monoamine levels was extremely challenging since it requires both quantitative detection and spatial resolution: the two analytical requisites are generally fulfilled using different techniques. The best quantitative performance is currently guaranteed by a chromatograph equipped with a detector (e.g., liquid chromatograph-electrochemical detector and gas chromatograph-mass spectrometer [Aragon et al., 2017, Miyajima et al., 2017]). Such instruments can be combined to sampling techniques, such as laser microdissection, to map quantities of a target molecule (Franck et al., 2013). However, these approaches are highly time consuming and are not suitable for high-throughput analyses. Alternatively, histochemical fluorescence-based imaging techniques, such as the Falck-Hillarp method (Falck and Hillarp, 1959) and IHC as well as ARG, have been used to identify the presence of monoamine nuclei in the brain (Fuxe et al., 2007, Masuoka and Alcaraz, 1975). The discovery of the monoaminergic neurons was achieved using the Falck-Hillarp method (Carlsson et al., 1962, Falck and Hillarp, 1959, Falck and Torp, 1962) by which fluorescence signals from derivatives of monoamines can be observed under a microscope. The method was the first useful method for histochemical monoamines analysis; however, its specificity and precision were not sufficient to perform a quantitative comparison or monitoring of monoamine levels. IHC for conjugated (immunoreactive) monoamines was developed at a later time, and most data about monoaminergic pathways are based on IHC data. However, IHC is affected by intrinsic variability and is not robust enough to compare large sets of samples (Nielsen et al., 2006). Our atlas showed a more physiological distribution of the monoamines than the previously reported ones (Steinbusch, 1981) since no drugs were administered before brain imaging and the samples were neither perfused nor fixed. ARG, which includes positron emission tomography, conjugates high spatial resolution, sensitivity, and quantitative performance (Masuoka and Alcaraz, 1975, Takano, 2018). However, despite being useful to chase injected molecules, it cannot detect unmodified brain monoamines.

Our data showed that monoamine quantities vary between the cell bodies and axon terminals, thereby possibly reflecting the different rates of synthesis or transport within single neurons. Indeed, previous studies have shown the different metabolic activities in the nuclei innervated by the monoaminergic axons supporting the former view (Halaris et al., 1976, Kim et al., 2005). It should be considered that the secreted monoamines either mediate synaptic transmission or diffuse in the extracellular space and bind to distant, extrasynaptic receptors on multiple target neurons. The latter type of transmission, referred to as volume transmission (Fuxe et al., 2010), involves a significant proportion of the axon terminals of monoaminergic neurons and helps to control various brain functions, despite their small number. MSI likely detects monoamines belonging to both pools. However, it cannot discriminate between intracellular compartments (i.e., soma, dendrites, and axons) and intracellular/extracellular monoamines, since the minimum spatial resolution for the monoamines is about 1–3 μm (Kaya et al., 2018, Kompauer et al., 2016, Passarelli et al., 2017). Although a higher spatial resolution <1 μm may be achieved using secondary ion mass spectrometry in the near future (Kaya et al., 2018, Passarelli et al., 2017), the use of MSI combined with other techniques that detect extracellular monoamines (i.e., microdialysis [Chen et al., 1999], fast-scan cyclic voltammetry [Borue et al., 2010]) as well as optogenetics (Takata et al., 2018, Watanabe et al., 2018) or fiber photometry (Tsutsui-Kimura et al., 2017) is essential to better understand the monoaminergic regulation of animal behaviors. We anticipate that further MSI analysis of monoamine turnover will provide direct evidence of synthesis, transport, and regional availability of the brain monoamines.

In the course of this study, we identified several brain nuclei in which high concentrations of 5-HT and one catecholamine overlapped, indicating that they are nodes of reciprocal regulation between different monoaminergic networks. Indeed, several reports have shown the functional interaction of multiple monoamines in some nuclei (e.g., 5-HT and NE at the DRN [Baraban and Aghajanian, 1980, Pudovkina et al., 2002], 5-HT and DA at the SN [Burke et al., 2014, Cobb and Abercrombie, 2003, Demireva et al., 2018, Lindgren et al., 2010], and 5-HT and DA at the VTA [Alex and Pehek, 2007, Prisco et al., 1994]). The sites of DA production (the SN and VTA) showed high 5-HT levels, thereby indicating that the dopaminergic system may be controlled by 5-HT (Doly et al., 2017, Niederkofler et al., 2015). Similarly, the central site of 5-HT production, which is the DRN, showed a high NE level, indicating a similar relationship. By contrast, in the site of NE production, the level of other monoamines was not high in the LC. Therefore, LC may play a central role in monoaminergic regulation (Figure S6). We do not exclude the possibility that neurotransmitters, other than 5-HT or DA, regulate noradrenergic neurons in the LC, in analogy with a proposed model in the rat brain (Aston-Jones, 2004).

We performed the metabolic characterization of 5-HT-rich nuclei manipulating the levels of Trp in the blood, a procedure known to affect both the 5-HT synthesis in the brain and anxiety-like behavior in the BALB/c mice. We note that C57BL/6J mice commonly show reductions of Trp and 5-HT levels after the ATD treatment, although their behavior was unchanged (Biskup et al., 2012). Such differences are likely due to more serotonergic (TPH2 positive) neurons and higher 5-HT content of C57BL/6J mice when compared with BALB/c mice (Bach et al., 2011).

We identified two brain nuclei (BLA/BMA and PVT) with 5-HT kinetics similar to the major raphe nuclei (DRN and MRN). In these nuclei, the 5-HT concentrations closely followed the availability of Trp in the blood, rapidly decreasing after the depletion of Trp, and were rescued by newly synthetized 5-HT during the administration of Trp. Therefore, they may be correlated to anxiety-like behavior associated with ATD. Indeed, the 5-HT levels in the BLA/BMA, DRN, and MRN regulate anxiety-like behavior (Johnson et al., 2015, Marcinkiewcz et al., 2016, Teissier et al., 2015). Notably, PVT neurons project into the amygdala, and the inhibition of PVT causes abnormal fear responses (Do-Monte et al., 2015, Kirouac, 2015, Do Monte et al., 2016, Penzo et al., 2015) and depressive-like behavior (Kasahara et al., 2016). However, as these studies are based on PVT inhibition or deletion, they do not provide information about the effect of 5-HT in PVT on behavior (Pratelli et al., 2017, Steinbusch, 1981, Vertes et al., 2010). The findings of the present study indicate that 5-HT in PVT may play an important role in the control of anxiety-like behavior, which may have been substantially overlooked. We anticipate that the 5-HT_7_ receptor could be involved because of its highly localized expression in the PVT (Neumaier et al., 2001).

We revealed that newly synthesized 5-HT is rapidly conveyed from the raphe nuclei to the PVT and SN, rather than to the rHip. We considered that the delivery to the PVT, SN, and rHip could be summarized, as depicted in Figure 6D. If the levels of peripheral Trp are not limiting, 5-HT neurons in the DRN and MRN convert Trp to 5-HT and provide the new 5-HT preferentially to the PVT and SN rather than rHip. When the levels of peripheral Trp decrease, as in the ATD model or in our previous model of chronic immune activation (Miyajima et al., 2017), the delivery of 5-HT to PVT and rHip significantly decreases, whereas that to the SN is less affected. Supplementation with Trp restored the supply of 5-HT to the PVT and SN but not to the rHip. Our results support the idea that the serotonergic nervous system targets different brain regions with different priorities. This finding could facilitate a better understanding of the complexity of the serotonergic network in the brain.

### Limitations of the Study

In this study, the whole brain atlas of 5-HT, DA, and NE was built using an animal (8-week-old C57BL/6J male mouse) as a preceding model. We confirmed consistency and reproducibility regarding high levels of 5-HT in DRN, MRN, SN, PVT, rHip, cHip, and BLA/BMA. However, further studies examining other strains at different ages are required to establish a generalized atlas.

Despite the fact that MSI can be used to identify specific brain nuclei in which monoamines are dynamically fluctuated, it cannot distinguish changes in extracellular spaces from those in the intracellular fraction. By using other techniques to monitor released monoamine concentrations, such as microdialysis or fast-scan cyclic voltammetry, complementary information that is essential for understanding how the alteration in monoamine metabolism affects animal behavior can be obtained.

## Methods

All methods can be found in the accompanying Transparent Methods supplemental file.

## Supplemental Information

Document S1. Transparent Methods and Figures S1–S7

Data S1. The Monoamine Atlas of the Mouse Brain, Related to Figure 2A
