# Supplementary material for: Detection of a High-Turnover Serotonin Circuit in the Mouse Brain Using Mass Spectrometry Imaging
Source: iScience. 2019 Sep 27;20:359–72. doi: 10.1016/j.isci.2019.09.036 (PMC6818351; doi:10.1016/j.isci.2019.09.036)
Supplement: Document S1. Transparent Methods and Figures S1–S7 [file mmc1.pdf]

ISCI, Volume *20*

Supplemental Information

Detection of a High-Turnover Serotonin Circuit in the Mouse Brain Using Mass Spectrometry Imaging

Eiji Sugiyama, Matteo M. Guerrini, Kurara Honda, Yuko Hattori, Manabu Abe, Patrik Källback, Per E. Andrén, Kenji F. Tanaka, Mitsutoshi Setou, Sidonia Fagarasan, Makoto Suematsu, and Yuki Sugiura

Contents

Figure S1. Distribution of 5-HT and $^{13}C^{15}N$-5HT used for the analysis of the ATD model, Related to Figure 5.

Figure S2. Relative concentrations of 5-HT in the brain nuclei, Related to Figure 5.

Figure S3. Disruption of TPH1 does not affect 5-HT levels in PVT, Related to Figure 5.

Figure S4. Levels of $^{13}C^{15}N$-5-HT in the brain of the ATD mice, Related to Figure 5.

Figure S5. Preferential delivery of newly synthesized $^{13}C^{15}N$-5-HT from the DRN and MRN to the PVT and SN, Related to Figure 6.

Figure S6. Schematic illustration of the monoaminergic main pathways based on levels of the monoamines, Related to Figure 2.

Figure S7. Mass spectra including target signals obtained by the 3 types of mass spectrometers, Related to Figure 1.

Transparent Methods

Supplemental References

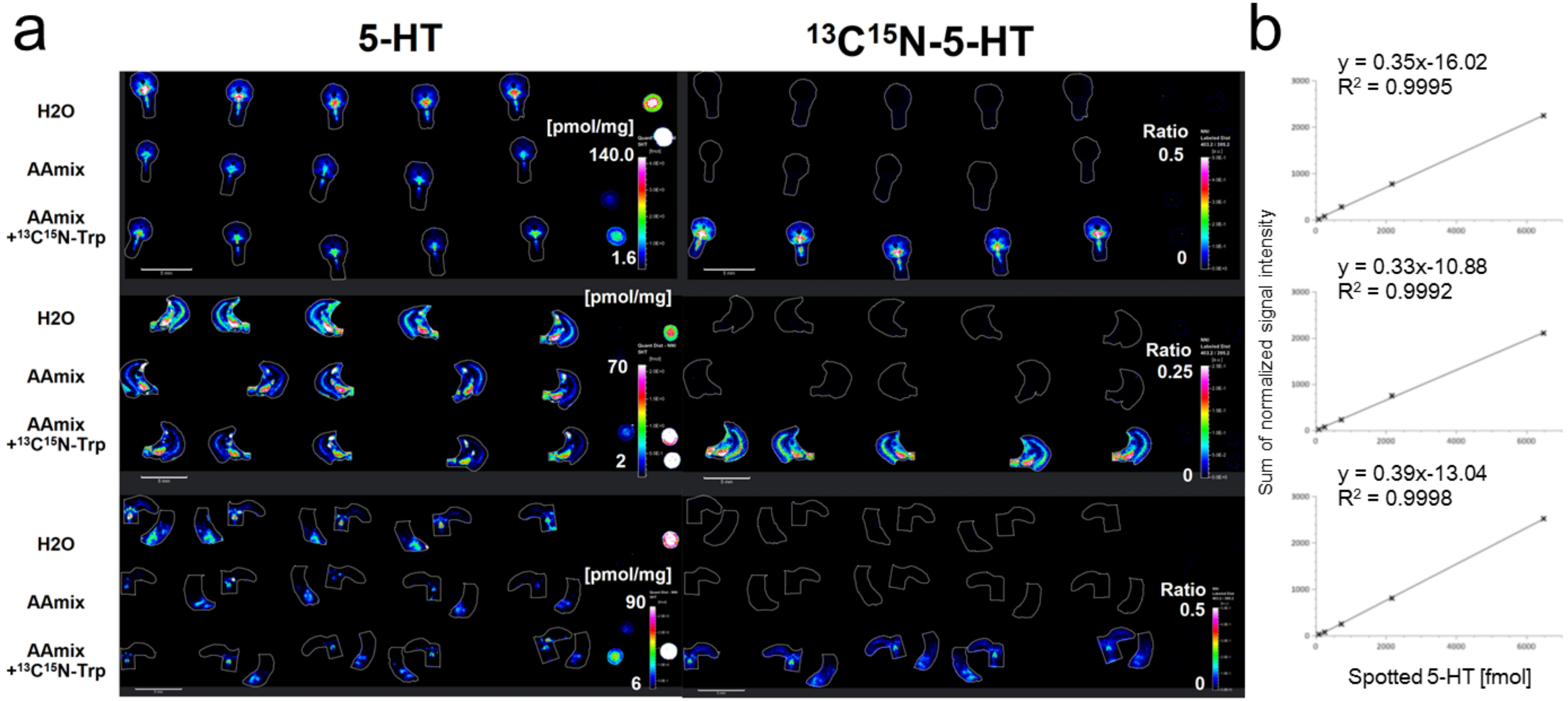

Figure S1. Distribution of 5-HT and $^{13}C^{15}N$-5HT used for the analysis of the ATD model, Related to Figure 5.

All the ion images used for the bars in **Figure 5E** were shown. The left (for 5HT-DPP) and the right (for $^{13}C^{15}N$-5HT-DPP) were obtained from the same mass spectra. Color scales show pmol/mg tissue for 5HT-DPP and relative intensity normalized by D_4-5HT-DPP for $^{13}C^{15}N$-5HT-DPP. **(b)** Calibration curves used for quantifying amount of 5-HT in site 1 (top), site 2 (middle), and site 3 (bottom). The data were obtained by the FT-ICR mass spectrometer.

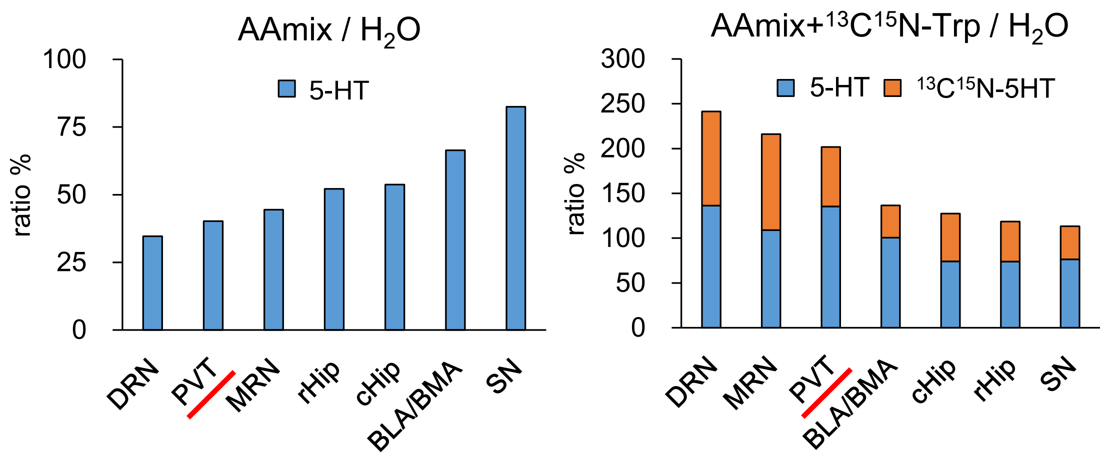

Figure S2. Relative concentrations of 5-HT in the brain nuclei, Related to Figure 5.

Left, relative levels of 5-HT in brain nuclei of the BALB/c male mice orally administrated with the AAmix normalized on the same nuclei of the group orally administrated with H_2O; **Right**, relative levels of 5-HT in brain nuclei of the mice orally administrated with the AAmix+$^{13}C^{15}N$-Trp group normalized on the same nuclei of the group orally administrated with AAmix only. The data were obtained by the FT-ICR mass spectrometer.

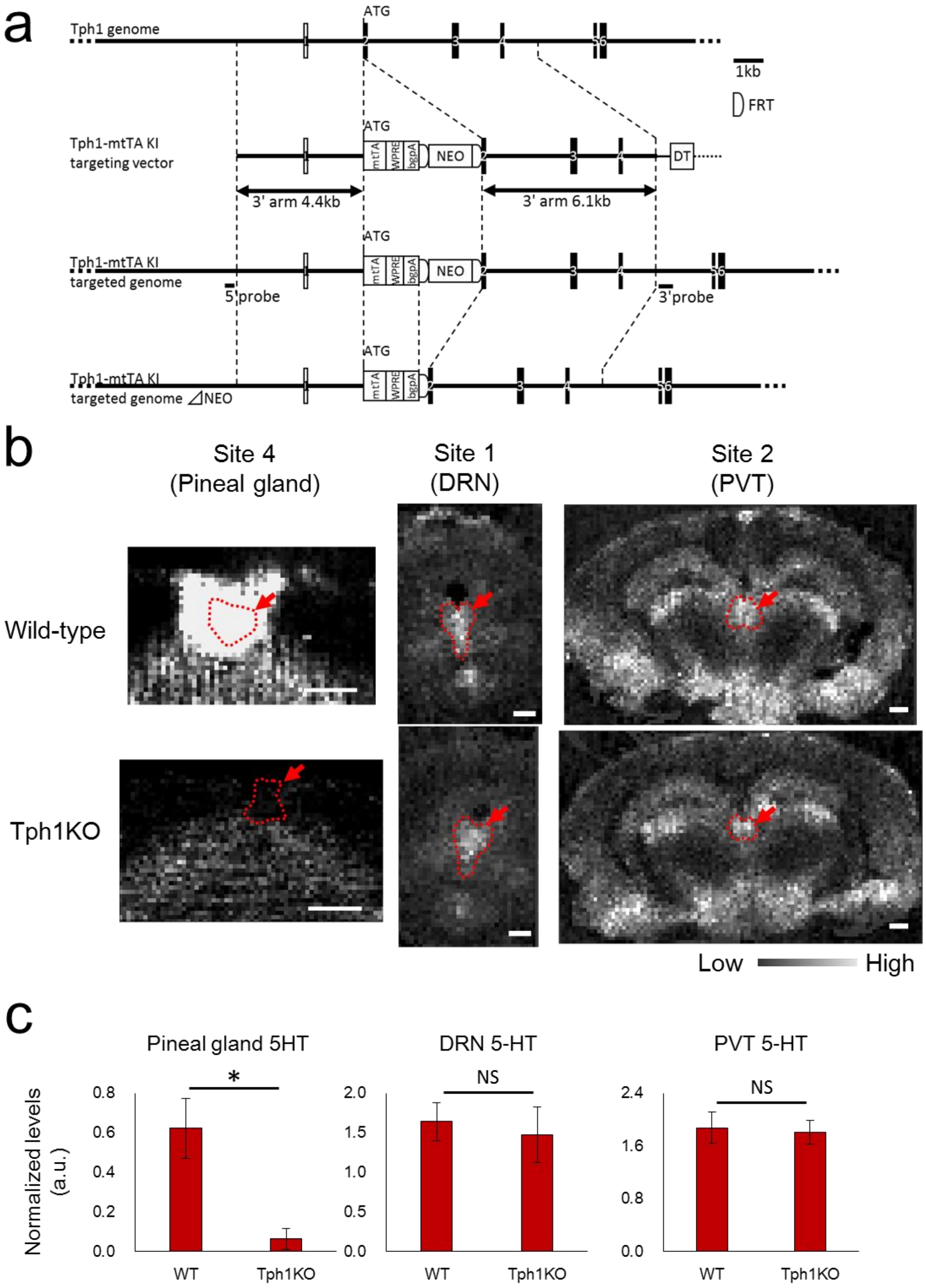

Figure S3. Disruption of TPH1 does not affect 5-HT levels in PVT, Related to Figure 5.
(a) Construction of Tph1-mtTA knock-in targeting vector. **(b)** Distribution of 5-HT in the three slices containing the pineal gland, DRN, or PVT. The regions were indicated by red arrows. The

area of the pineal gland was identified with H&E stain for a consecutive section. In wild-type, 5-HT was intensely detected from pineal gland as well as lumen of blood vessels. Scale bar = 500 μm. (**c)** 5-HT levels in the three regions. Data are shown in mean ± SD (n = 5 wild-type male and female mice at 8 weeks old and n = 3 Tph1-knockout male and female mice at 8 weeks old for the pineal gland, n = 4 male and female mice at 8 weeks old per group for the DRN, and n = 5 male and female mice at 8 weeks old per group for the PVT). NS: not significant ($P > 0.05$); *: $P < 0.05$ (Welch's t test). The data were obtained by the LIT mass spectrometer.

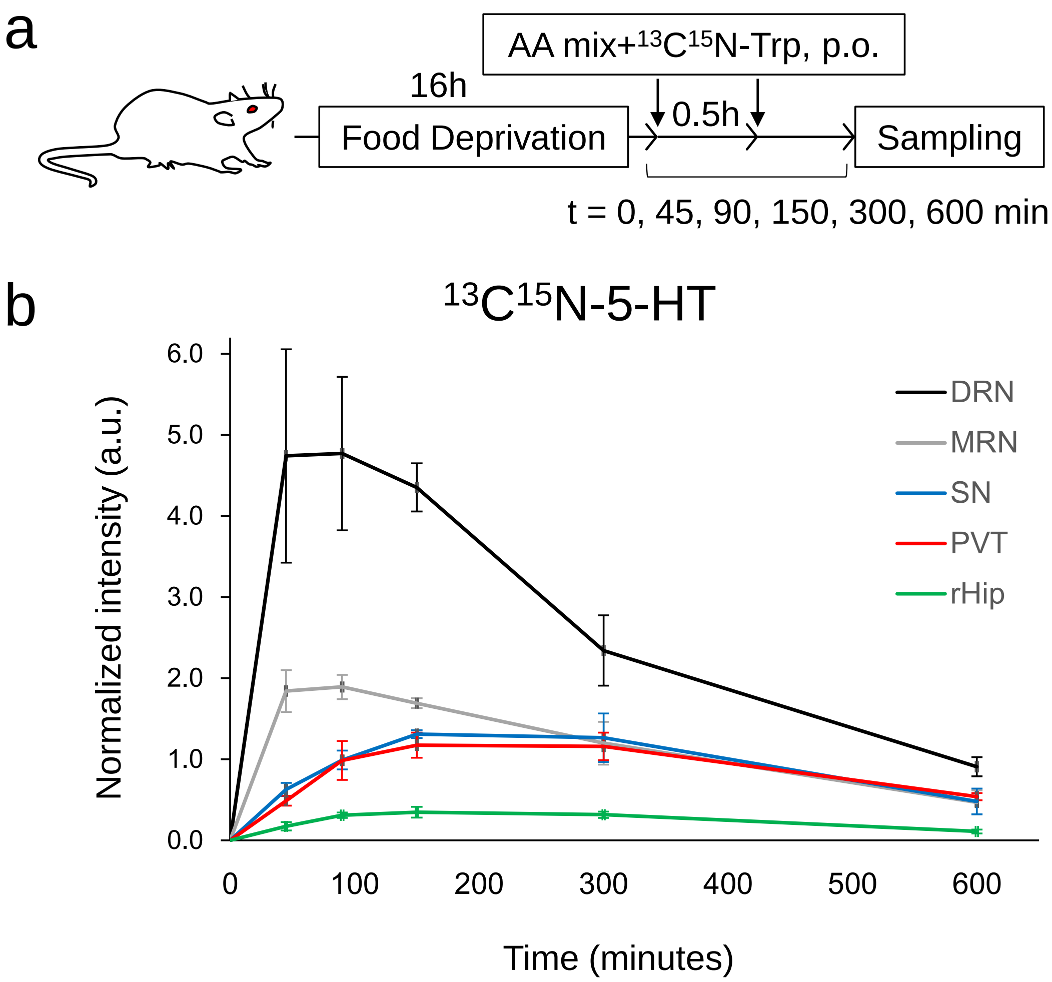

Figure S4. Levels of $^{13}C^{15}N$-5-HT in the brain of the ATD mice, Related to Figure 5.

Time course of newly-synthesized 5-HT in the indicated brain regions. (**a)** Schematics of the experimental procedure. After 16 h of food depletion, the BALB/c male mice at 10-11 weeks old were orally administered the AAmix+$^{13}C^{15}N$-Trp at t = 0 h and t = 0.5 h. Sampling of the brain was performed at the indicated time points. (**b)** Levels of newly-synthesized 5-HT ($^{13}C^{15}N$-5-HT) in the indicated brain regions. Black, white, blue, red, green lines connect the mean levels of $^{13}C^{15}N$-5-HT in the DRN, MRN, SN, PVT, and rHip at the indicated times. Error bars show SD (n = 4 mice). The data were obtained by the LIT mass spectrometer.

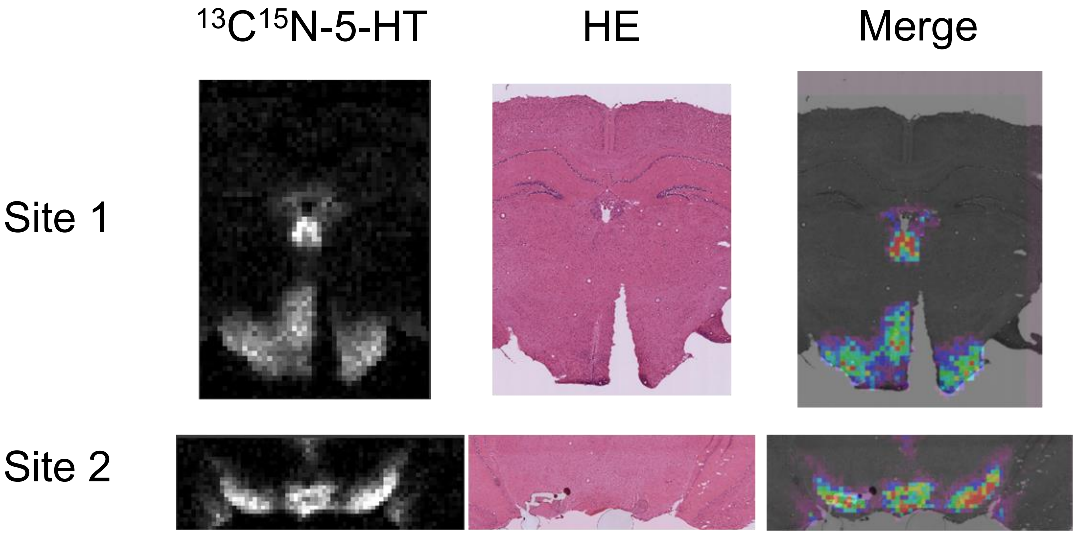

Figure S5. Preferential delivery of newly synthesized $^{13}C^{15}N$-5-HT from the DRN and MRN to the PVT and SN, Related to Figure 6.

Images of $^{13}C^{15}N$-5-HT, HE staining, and the merged are shown in left, center, right, respectively. The data were obtained by the LIT mass spectrometer.

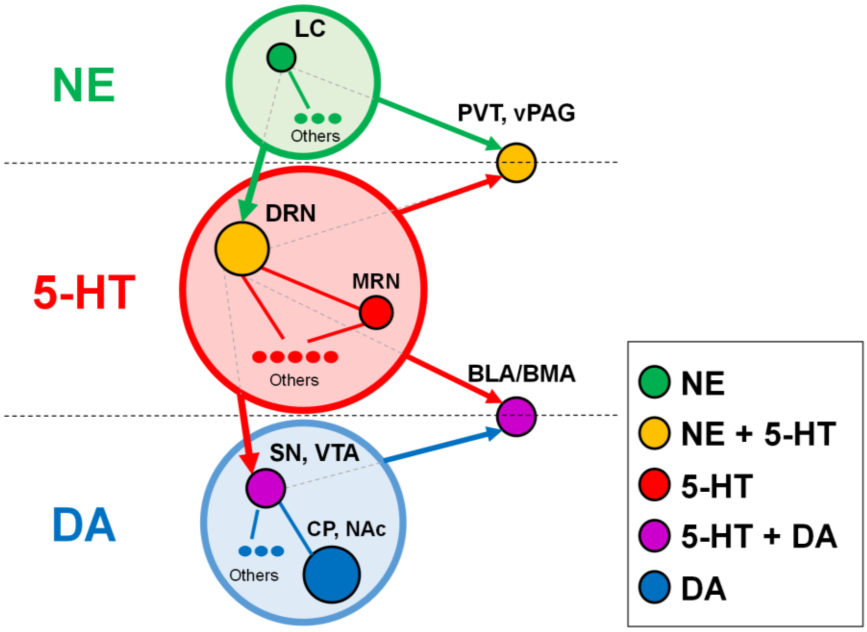

Figure S6. Schematic illustration of the monoaminergic main pathways based on levels of the monoamines, Related to Figure 2.

BLA/BMA: basolateral/ basomedial amygdala, CP: caudate-putamen; DRN: dorsal raphe nucleus, LC: locus coeruleus; MRN: median raphe nucleus, NAc: nucleus accumbens; PVT: paraventricular nucleus of the thalamus, SN: substantia nigra, vPAG: periaqueductal gray, ventral part; VTA: ventral tagmental area.

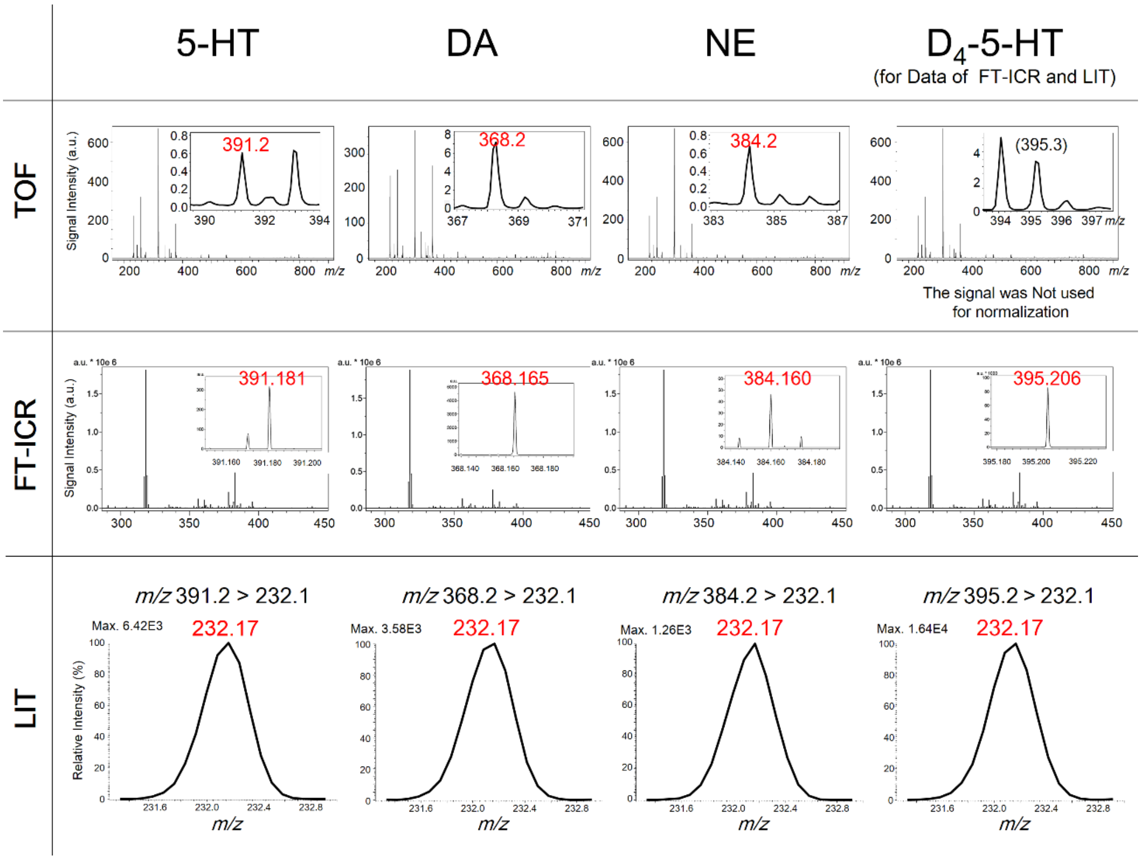

Figure S7. Mass spectra including target signals obtained by the 3 types of mass spectrometers, Related to Figure 1.

Representative mass spectra obtained with the TOF (top), the FT-ICR (middle), and the LIT (bottom) mass spectrometer are shown. Spectra for 5-HT, NE, and D_4-5-HT were obtained from the paraventricular nucleus of the thalamus, while the spectra for DA were obtained from the caudate putamen. Note that signal derived from D_4-5-HT was used as internal standard for not TOF but FT-ICR and LIT. The TOF data were normalized with TIC and the signal intensity from standard spots of 5-HT.

Transparent Methods

Chemicals

We obtained D_4-5HT creatinine sulfate complex from IsoSciences (PA, USA), tetrafluoroborate salts of 2,4-diphenyl-pyrylium (DPP) from Sigma-Aldrich (MO, USA), 2,5-dihydroxybenzoic acid (DHB) from Bruker Daltonics (MA, USA), $^{13}C_{11}{}^{15}N_2$-Tryptophan (^{13}C 97%, ^{15}N 95%) ($^{13}C^{15}N$-Trp) from Taiyo Nippon Sanso (Tokyo, Japan).

Mice

C57BL/6J male or BALB/cByJ male mice at 8-12 weeks old were purchased from Japan SLC (Hamamatsu, Japan) or CLEA Japan (Tokyo, Japan). All animal experimental procedures were approved by the Experimental Animal Committee of the Hamamatsu University School of Medicine and Keio University School of Medicine. After cervical dislocation euthanasia, the brains of the mice were isolated and immediately frozen in powder dry ice and then stored at −80°C. ATD model was prepared according to a protocol used in previous studies (Biskup et al., 2012). Briefly, after a food depletion for 16 h, the BALB/c male mice at 10-11 weeks were treated by gavage with amino acid mixture (AAmix), AAmix with labeled tryptophan (AAmix+$^{13}C^{15}N$-Trp) or water two times with an interval for 30 min. The mice were killed to obtain blood and brain after 150 min of the initial gavage. The brains were collected as described above, and the whole bloods were immediately collected from the hearts and then frozen with powdered dry ice. For time course analysis, brains of the BALB/c male mice at 10-11 weeks belonging to the group of AAmix+$^{13}C^{15}N$-Trp were collected at 0, 45, 90, 150, 300, 600 min. We allocated the mice randomly into experimental groups, and we did not use masking during data collection and data analysis.
Infusion of $^{13}C^{15}N$-Trp into DRN and MRN was performed as follows: the BALB/c male mice at 12 weeks were anesthetized with 2% isoflurane and were implanted stereotaxically with a guide cannula (CXG-4, Eicom, Kyoto, Japan) directed between DRN and MRN the prefrontal cortex (AP –4.5 mm, ML –0.0 mm, DV –3.0 mm from the bregma). Cannula was inserted with a 10° angle to avoid damage to the superior sagittal sinus. The guide cannula was immobilized with dental acrylic and filled with a dummy cannula (CXD-4). After the surgery, the mice were housed individually for at least 1 week before the following infusion. After infusing $^{13}C^{15}N$-Trp 50 mM, diluted in Ringer's solution (147 mM Na^+, 4.0 mM K^+, 2.3 mM Ca^{2+} and 156 mM Cl^-) by use of an injection cannula (CX) and a microinfusion pump (Fusion 100T, Chemyx, TX, USA) with a gas-tight syringe at a rate of 0.05 µL/min, mice were immediately anesthetized with isoflurane and the brains were collected.
We used male and female TPH1-deficient mice at 8 weeks old, which are the Tph1-mammalianized tTA (mtTA) homozygotes prepared as follows. A C57BL/6 BAC genomic clone RP23–4G4, which contains the Tph1 gene, was isolated from a RP23 mouse genomic BAC library

(Advanced GenoTechs, Tsukuba, Japan). The Quick and Easy BAC Modification Kit (Gene Bridges) was used for vector construction. We designed a gene-targeting vector in which the mtTA gene was placed just behind the translation initiation site of the Tph1 gene in frame (**Figure. S3**). Fragments of the mtTA coding sequence, woodchuck hepatitis virus posttranscriptional regulatory element (WPRE) and rabbit beta-globin poly(A) were ligated into pDTMC/FRTpgkgb2Neo (modified from pgkgb2Neo; Gene Bridges) containing a pgk/gb2–Neo cassette flanked by two *frt* sequences. This construct was used as the PCR template for the amplification of the fragment composed of the mtTA–Neo cassette. By using two primers, mtTA_KI_arm_F (5'-GTTCTGTGCTTAGCACTCATTTATCTGAACTTTCACACTTCAGATTCACCATGGCTCGCCTGGACAAGTC-3') and mtTA_KI_arm_R (5'-CTTTCGGAGGAATGGTCTTTGTTCTCTTTGTTCTCCTTGTTGTCTTCAATCACTATAGGGCTCGAGGAAG-3'), the cassette attached by 5' and 3' homology arms was amplified by PCR and then recombined into the translation initiation site of the Tph1 gene in RP23–4G4. The 10.5 kb fragment containing the exon 2–4 of the Tph1 gene and the iCre–Neo cassette was subcloned into pDT–MC#3 vector, including an MC1-promoter-driven diphtheria toxin gene. The resulting construct contained the translation initiation site of the Tph1 gene inserted by the mtTA–Neo cassette, 4.4 kb upstream and 6.1 kb downstream genomic sequences, and 4.4 kb pDT–MC#3. We introduced the linearized gene-targeting vector into the C57BL/6N-derived ES line RENKA (Mishina and Sakimura, 2007) and then selected recombinant clones under the medium containing 175 µg/mL G418. . The targeted clones were confirmed by Southern blot analysis and microinjected into eight-cell-stage embryos of a CD-1 mouse strain. The resulting chimeric embryos were developed to the blastocyst stage by incubation for ~24 h and then transferred to a pseudopregnant CD-1 mouse uterus. Germ-line chimeras were crossed with C57BL/6N female mice to establish the Tph1-mtTA mouse line.

Sample preparation for MALDI-MSI

Two male mouse brains were homogenized and frozen with an embedding dish at −80°C. The homogenate block or a whole brain was hold on a disc with an embedding medium (Super Cryoembedding Medium, SECTION-LAB, Hiroshima, Japan) and equilibrated at −16°C in cryostats (Leica Biosystems, Nussloch, Germany). Tissues were sectioned at 8-µm thick and thaw-mounted on a conductive indium-tin-oxide (ITO)-coated glass slides (Matsunami Glass Industries, Osaka, Japan). We spotted a blank and 5HT solutions (0.4 µL/spot, 0.2 to 16.2 µM with a dilution factor of 3, dissolved in 50% methanol) onto the homogenate sections and then sprayed D_4-5HT solution (10 µM in 50% methanol) on the sections as an internal standard (IS) with an automated sprayer, which is a TM-Sprayer (HTX Technologies, NC, USA) or a SunCollect

(SunChrom, Friedrichsdorf, Germany). The parameters for TM-Sprayer were set as follows: spray gas flow at 10 psi, nozzle temperature at 80°C, the number of spray cycles at 12, the speed of the nozzle movement at 1200 mm/min, and flow rate at 40 µL/min. The parameters for SunCollect were set as follows: spray gas flow at 29 psi, nozzle temperature at room temperature, the number of spray cycles at 10, the speed of the nozzle movement at 1000 mm/min, and flow rate at 20 µL/min. To perform on-tissue derivatization of monoamines, the DPP solution (1.3 mg/mL in methanol) was manually sprayed using an airbrush (Procon Boy FWA platinum; Mr. Hobby, Tokyo). The manual spray was performed at room temperature with 40 µL/mm^2 and a distance of approximately 50 mm. The DHB solution (40 mg/mL in 50% methanol) was sprayed on the sections in the same manner as that for the IS. DHB solution (40 mg/mL in 50% methanol) was sprayed on the sections by the same way for the IS.

MALDI-MSI

The samples were measured by a time-of-flight (TOF) type mass spectrumeter (ultrafleXtreme, Bruker Daltonics) or a MALDI-Fourier transformation ion cyclotron resonance (FT-ICR) mass spectrometer (solariX XR 7T, Bruker Daltonics), or a linear ion trap (LIT) mass spectrometer (LTQ XL, Thermo Fishcer Scientific). We selected an optimal mass analyzer depending on the purpose of the experiments. Each instrument has functional differences in regard to data acquisition speed, signal sensitivity, and acquired data size, which limit the available data analysis software. Owing to its high-speed data acquisition, the TOF mass spectrometer was used for screening monoamine rich in regions from among the whole mouse brain sections (Figure 2a), although its sensitivity is lower than those of others because of insufficient mass resolution to resolve small signals (Figure S8). An instrument with a FT-ICR mass analyzer, which has greater mass resolution (Figure S8), thus, can detect trace signals and was therefore used for the selected brain slice measurement to reconstruct the three-dimensional model (Figure 2b and Video S1) and for quantitative comparison with the calibration curves (Figures 4, 5, and S1). The disadvantage of FT-ICR MS is its larger data size than others. The LIT mass spectrometer, which has higher sensitivity and selectivity due to its larger capacity for ion trapping and specific monitoring of fragment ions produced by tandem mass spectrometry, was used for other quantitative comparison. Its data size is much smaller than those of others (Figure S8), although the speed of data acquisition is slower. The raster scan pitch was set depending on the experiment from 30 to 150 µm. Data were acquired from *m*/*z* 160 to 920 (for TOF data) or *m*/*z* 286.65 to 450.00 (for FT-ICR data) in positive ion mode. For LIT data, signals of 5-HT-DPP (*m/z* 391 > 232), $^{13}C_{10}{}^{15}N_2$-5-HT-DPP ($^{13}C^{15}N$-5-HT-DPP, *m/z* 403 > 233), D_4-5-HT-DPP (*m/z* 395 > 232) were monitored with a precursor ion isolation width of *m/z* 1.0 and a normalized collision energy of 45%. Ion images were reconstructed with flexImaging 4.1 (Bruker Daltonics), msIQuant

2.0.1.14(Källback et al., 2016), ImageQuest 1.1.0 software (Thermo Fischer Scientific) for the TOF data, the FT-ICR data, and the LIT data, respectively. Ion intensities of target ions were normalized with total ion current (for the TOF data), or a signal intensity of D_4-5HT-DPP (a signal at *m/z* 395.20615 for the FT-ICR data, and the signal monitored in LIT data). To quantify 5HT content, calibration curve was made using the signal intensity ratio (5HT-DPP to D_4-5HT- DPP) derived from the spots. We subtracted the value of blank spot subtracted from the values of each area. Quantity of $^{13}C^{15}N$-5HT was estimated by calculating a ratio ($^{13}C^{15}N$-5HT-DPP to 5-HT-DPP). Three dimensional images and a movie was constructed using SCiLS lab Premium3D software (SCiLS GmbH, Bremen, Germany). After finishing the measurement, the sections were stained by H&E stain to annotate the regions.

Sample preparation for LC-ESI-MS/MS

We mixed the frozen sections or the bloods with methanol containing an internal standard (L-methionine sulfone). We then added ultrapure water and chloroform. The mixture was centrifuged at 20,400 ×g for 15 min at 4°C. After centrifugation, the upper phase was filtered using an ultrafiltration tube (Ultrafree-MC, UFC3 LCC NB, Human Metabolome Technologies). The filtrate was dried with a vacuum concentrator (miVac, Genevac, UK), and then dissolved with ultrapure water (100 µL for bloods, 20 µL for sections) and was analyzed by LC-MS/MS. The lower and intermediate phases were dried up and the protein amount was determined using a BCA Protein Assay Kit (TAKARA BIO, Shiga, Japan).

LC-ESI-MS/MS

The metabolites in the samples were separated on the Discovery HS F5-3 column (2.1 mm I.D. × 150 mm, 3 µm particle, Sigma-Aldrich), using a gradient program with 0.1% formic acid in water (mobile phase A) and 0.1% formic acid in acetonitrile (mobile phase B). The gradient was scheduled as follows: 0.0–2.0 min, 0% B; 2.0–5.0 min, linear gradient to 25% B; 5.0–11.0 min, linear gradient to 35% B; 11.0–15.0 min, linear gradient to 95% B; 15.0–20.0 min, 95% B; 20.0–20.1 min, linear gradient to 0% B; 20.1–25.0 min, 0% B. The flow rate was set at 250 µL/min. The column temperature was maintained at 40°C. Triple-quadrupole mass spectrometers equipped with an electrospray ionization (ESI) ion source was used in the positive ion mode (LCMS-8040 for the blood samples and LCMS-8060 for the brain sections, Shimadzu). The mass spectrometers were operated in multiple reaction monitoring (MRM) mode with the following ion transitions: tryptophan *m/z* 205.1 > 188.2; $^{13}C^{15}N$-Trp *m/z* 218.1 > 200.05; 5HT *m/z* 177.1 > 132.1; $^{13}C^{15}N$-5HT *m/z* 189.1 > 142.1; L-methionine sulfone; *m/z* 182.0 > 56.1.

Supplemental References

Biskup, C.S., Sánchez, C.L., Arrant, A., van Swearingen, A.E.D., Kuhn, C., and Zepf, F.D. (2012). Effects of acute tryptophan depletion on brain serotonin function and concentrations of dopamine and norepinephrine in C57BL/6J and BALB/cJ mice. PLoS One 7, 7–14.

Källback, P., Nilsson, A., Shariatgorji, M., and Andrén, P.E. (2016). MsIQuant - Quantitation Software for Mass Spectrometry Imaging Enabling Fast Access, Visualization, and Analysis of Large Data Sets. Anal. Chem. 88, 4346–4353.

Mishina, M., and Sakimura, K. (2007). Conditional gene targeting on the pure C57BL/6 genetic background. Neurosci. Res. 58, 105–112.
