# Supplementary figures and images for: Detection of a High-Turnover Serotonin Circuit in the Mouse Brain Using Mass Spectrometry Imaging
Source: iScience. 2019 Sep 27;20:359–72. doi: 10.1016/j.isci.2019.09.036 (PMC6818351; doi:10.1016/j.isci.2019.09.036)

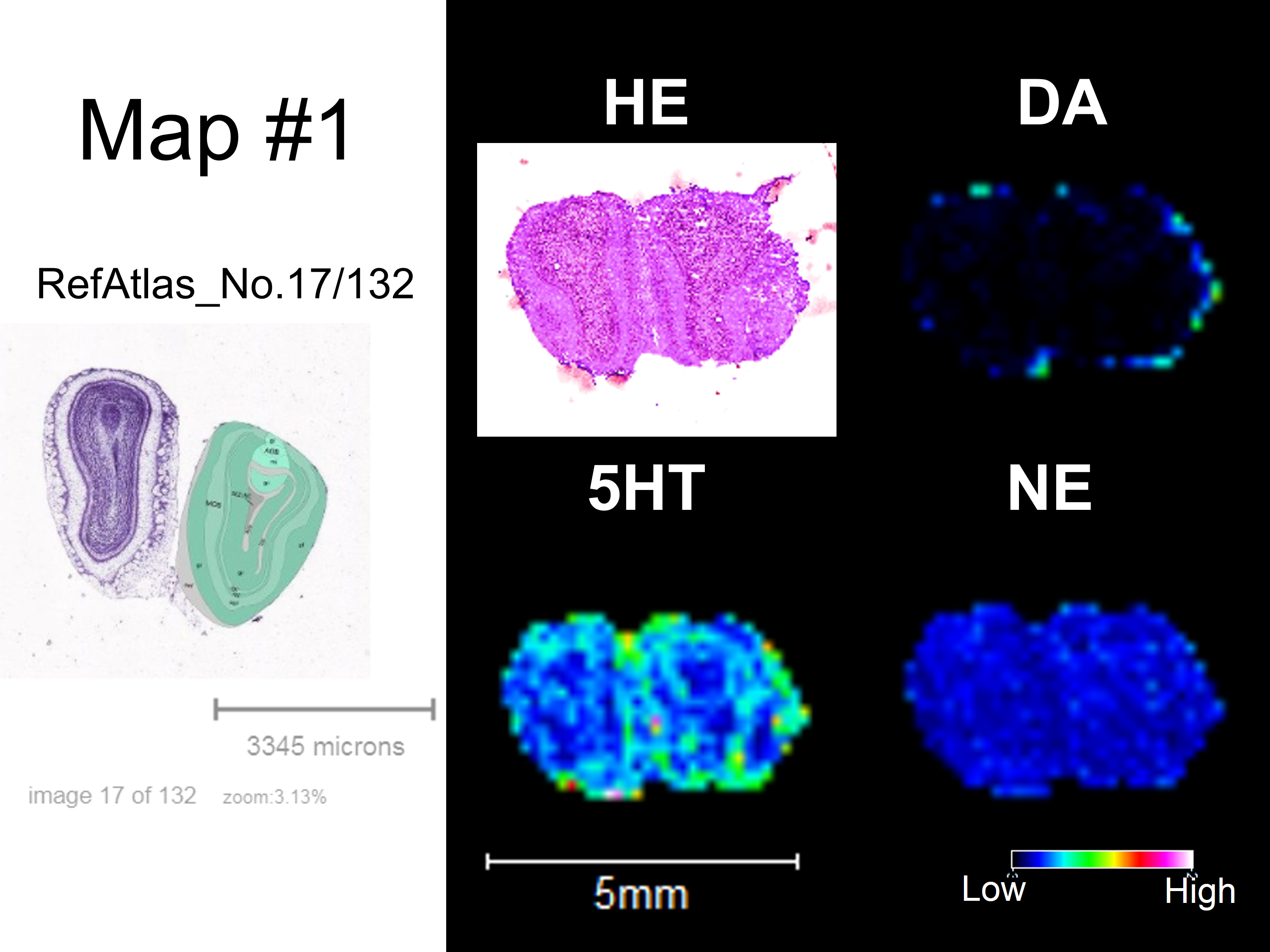

Supplement: Data S1. The Monoamine Atlas of the Mouse Brain, Related to Figure 2A [file mmc2.zip › Data1/âXâëâCâh1.TIF]

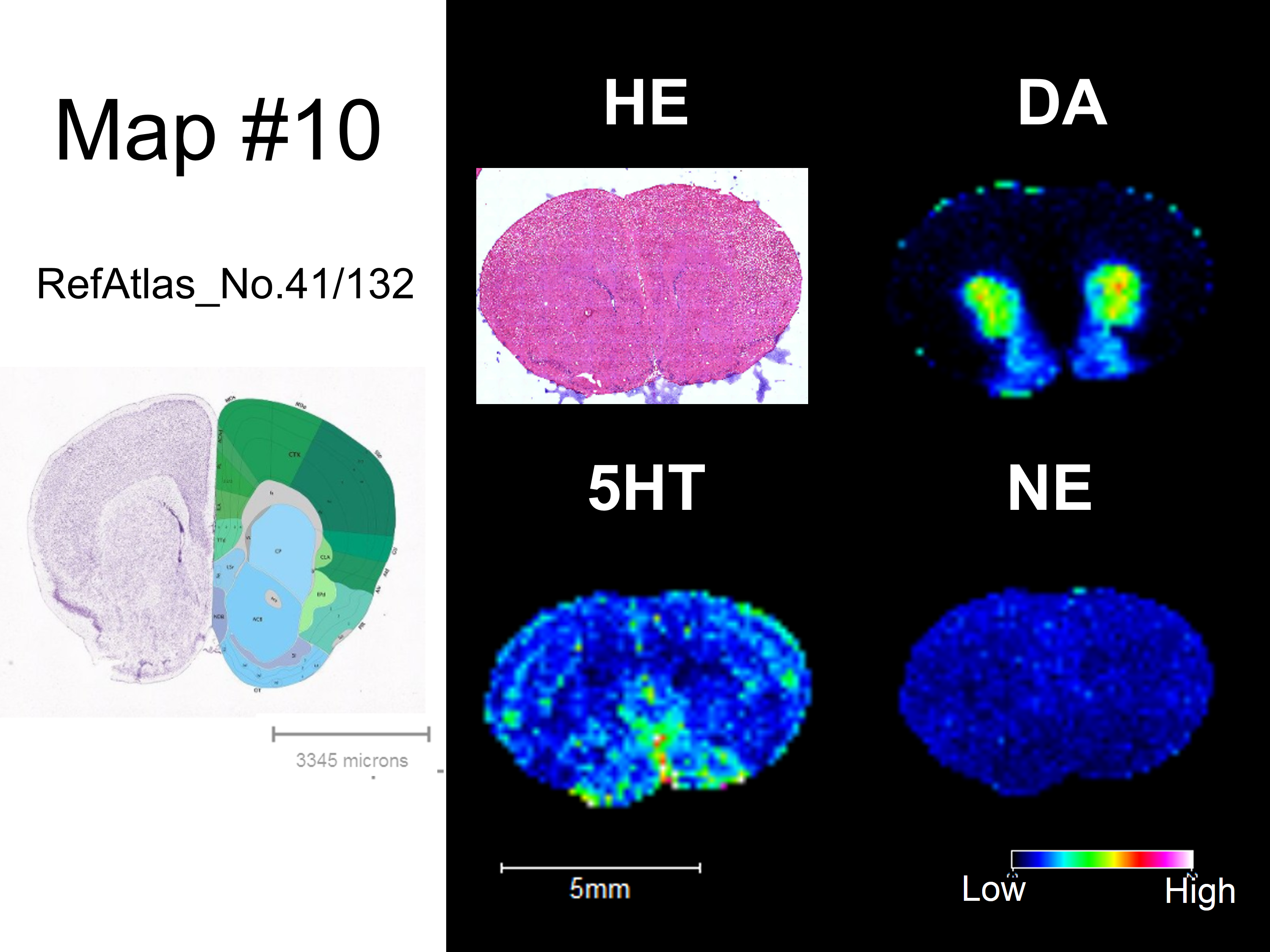

Supplement: Data S1. The Monoamine Atlas of the Mouse Brain, Related to Figure 2A [file mmc2.zip › Data1/âXâëâCâh10.TIF]

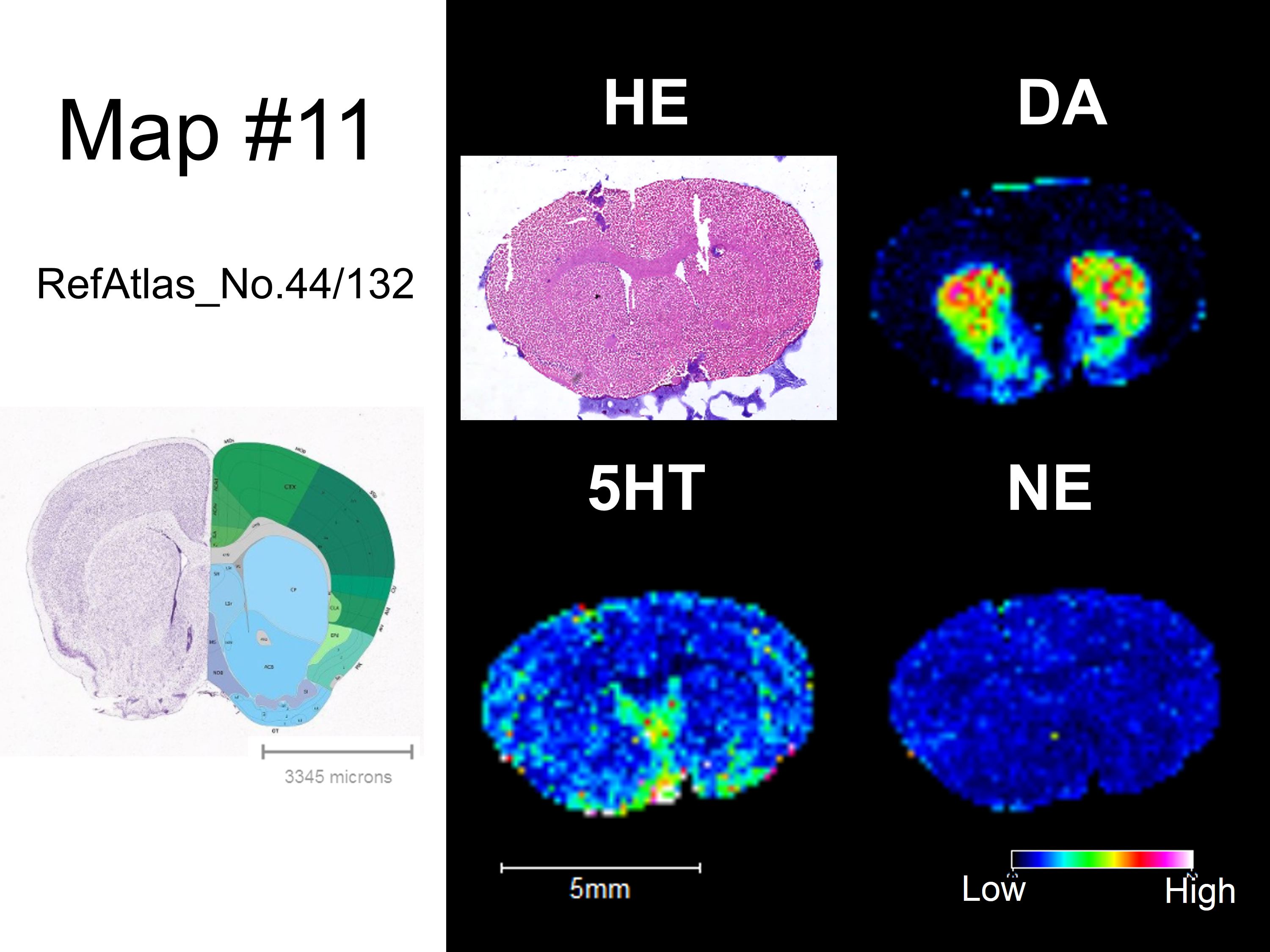

Supplement: Data S1. The Monoamine Atlas of the Mouse Brain, Related to Figure 2A [file mmc2.zip › Data1/âXâëâCâh11.TIF]

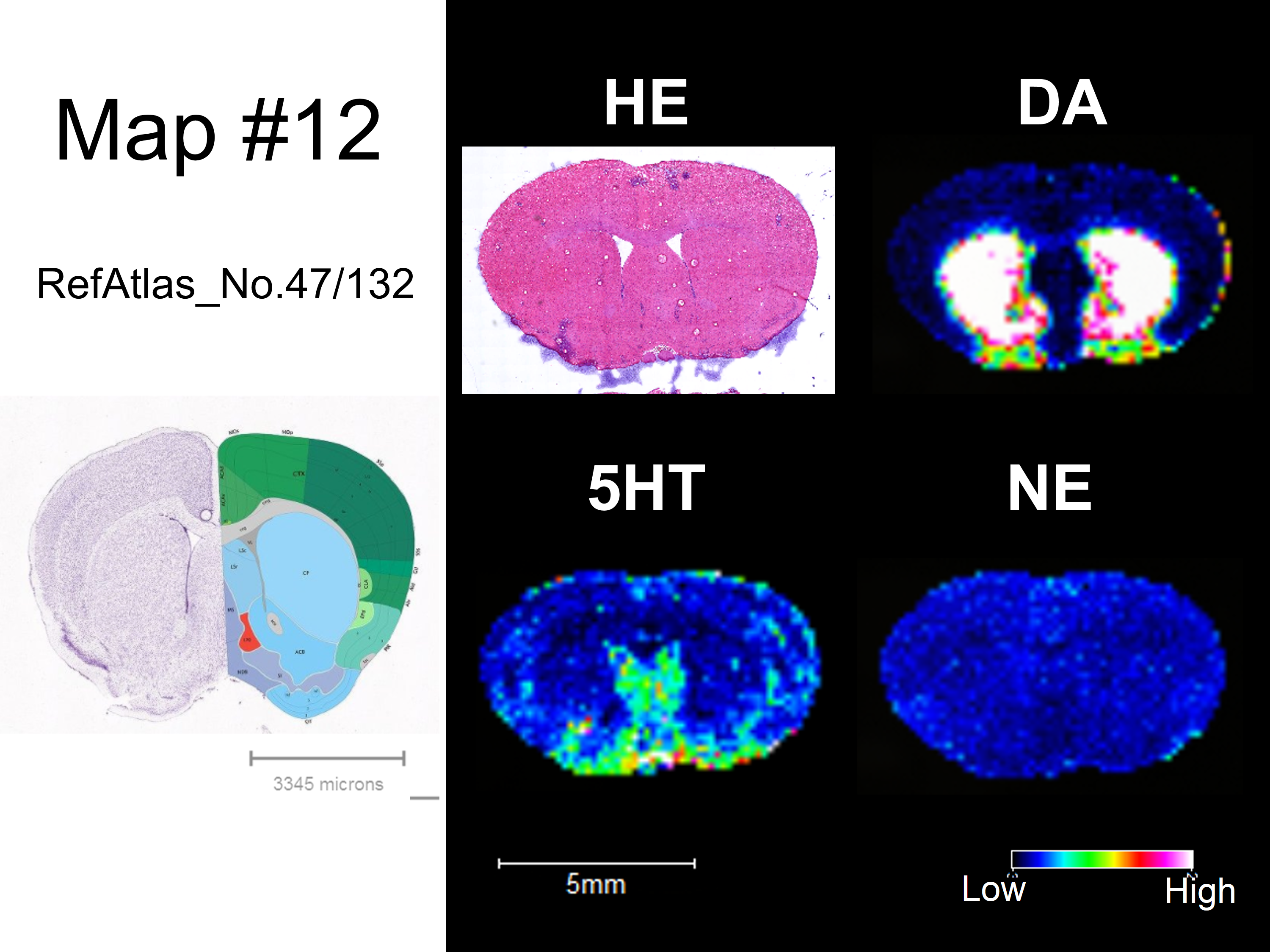

Supplement: Data S1. The Monoamine Atlas of the Mouse Brain, Related to Figure 2A [file mmc2.zip › Data1/âXâëâCâh12.TIF]

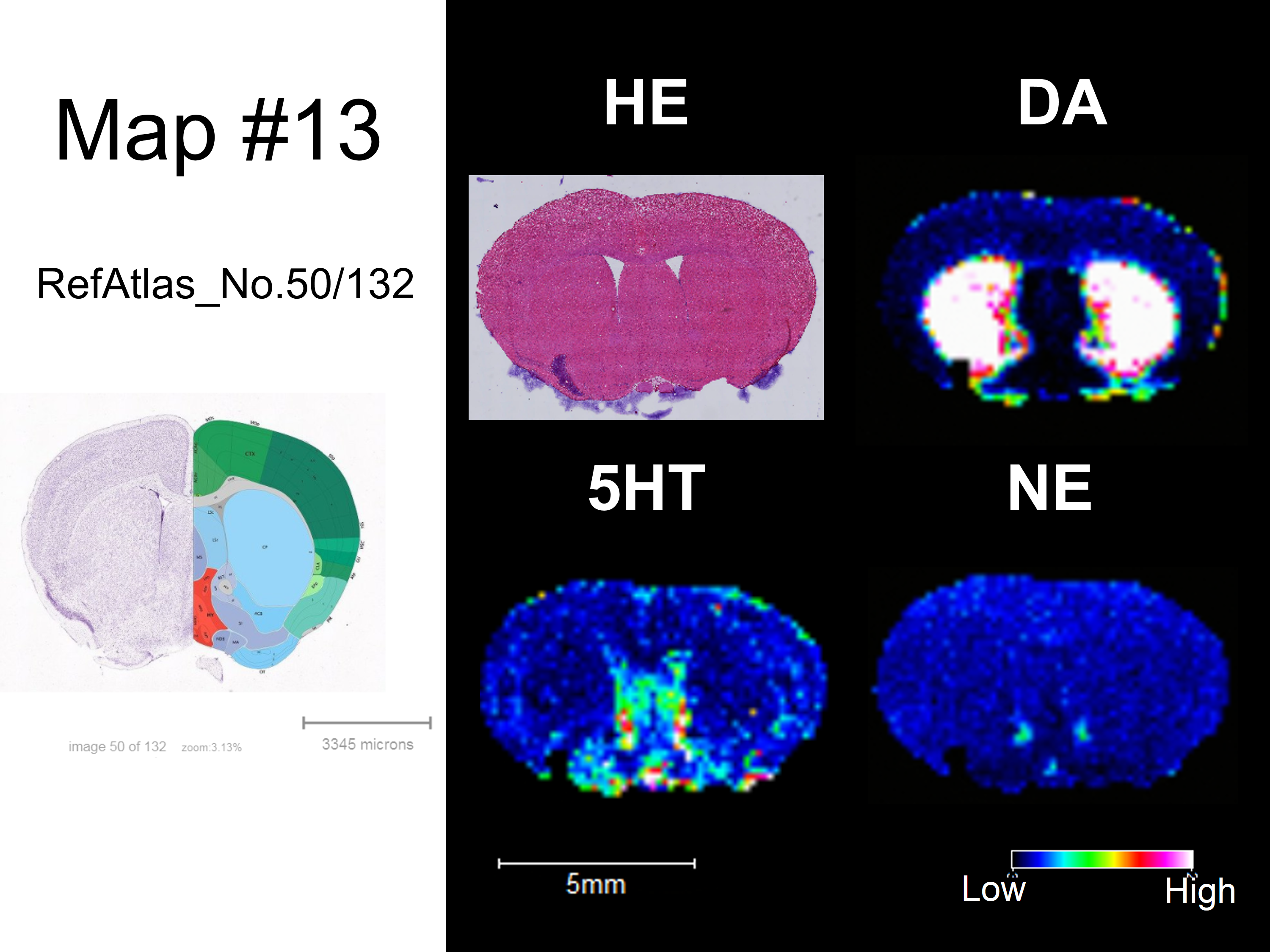

Supplement: Data S1. The Monoamine Atlas of the Mouse Brain, Related to Figure 2A [file mmc2.zip › Data1/âXâëâCâh13.TIF]

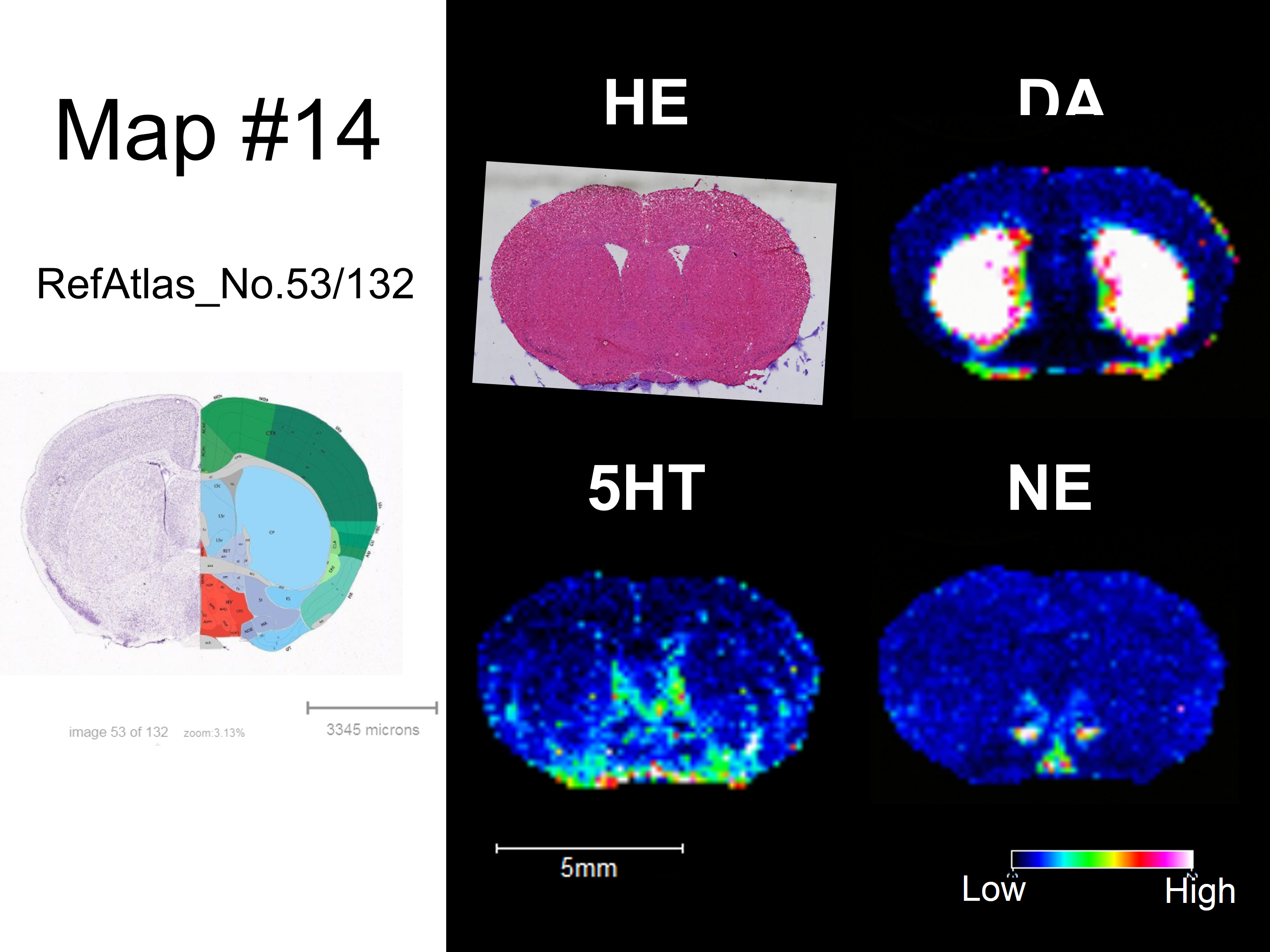

Supplement: Data S1. The Monoamine Atlas of the Mouse Brain, Related to Figure 2A [file mmc2.zip › Data1/âXâëâCâh14.TIF]

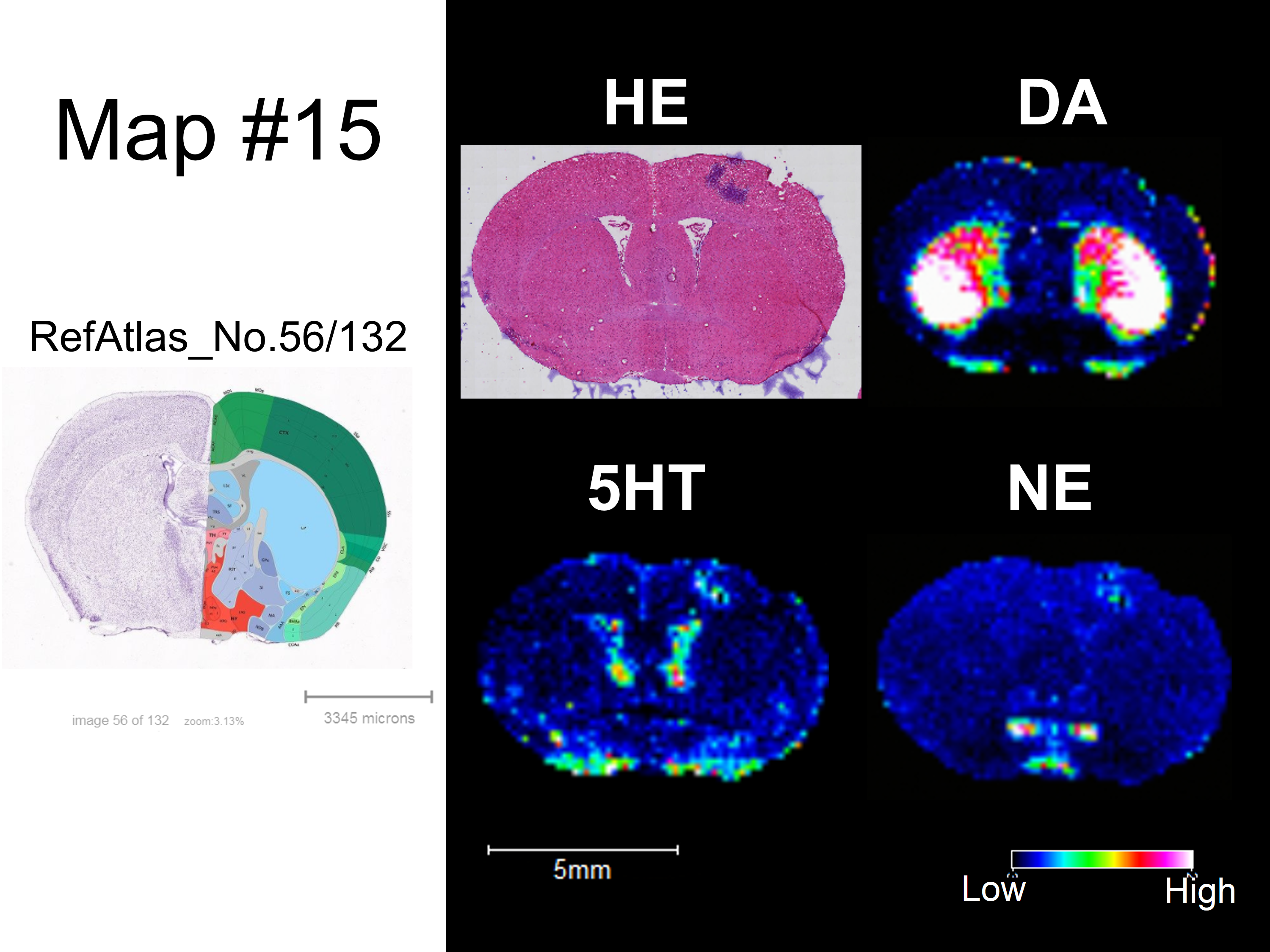

Supplement: Data S1. The Monoamine Atlas of the Mouse Brain, Related to Figure 2A [file mmc2.zip › Data1/âXâëâCâh15.TIF]

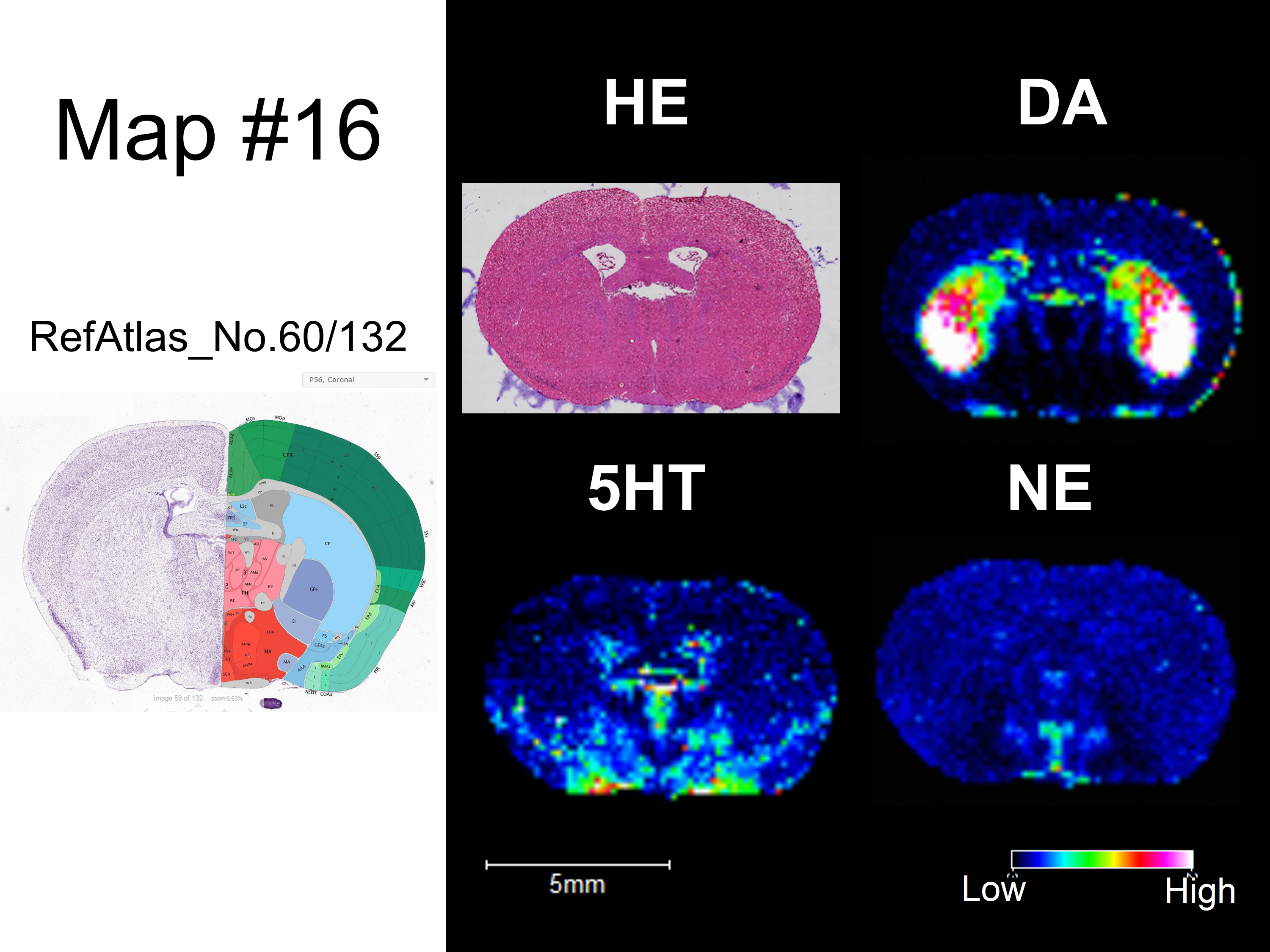

Supplement: Data S1. The Monoamine Atlas of the Mouse Brain, Related to Figure 2A [file mmc2.zip › Data1/âXâëâCâh16.TIF]

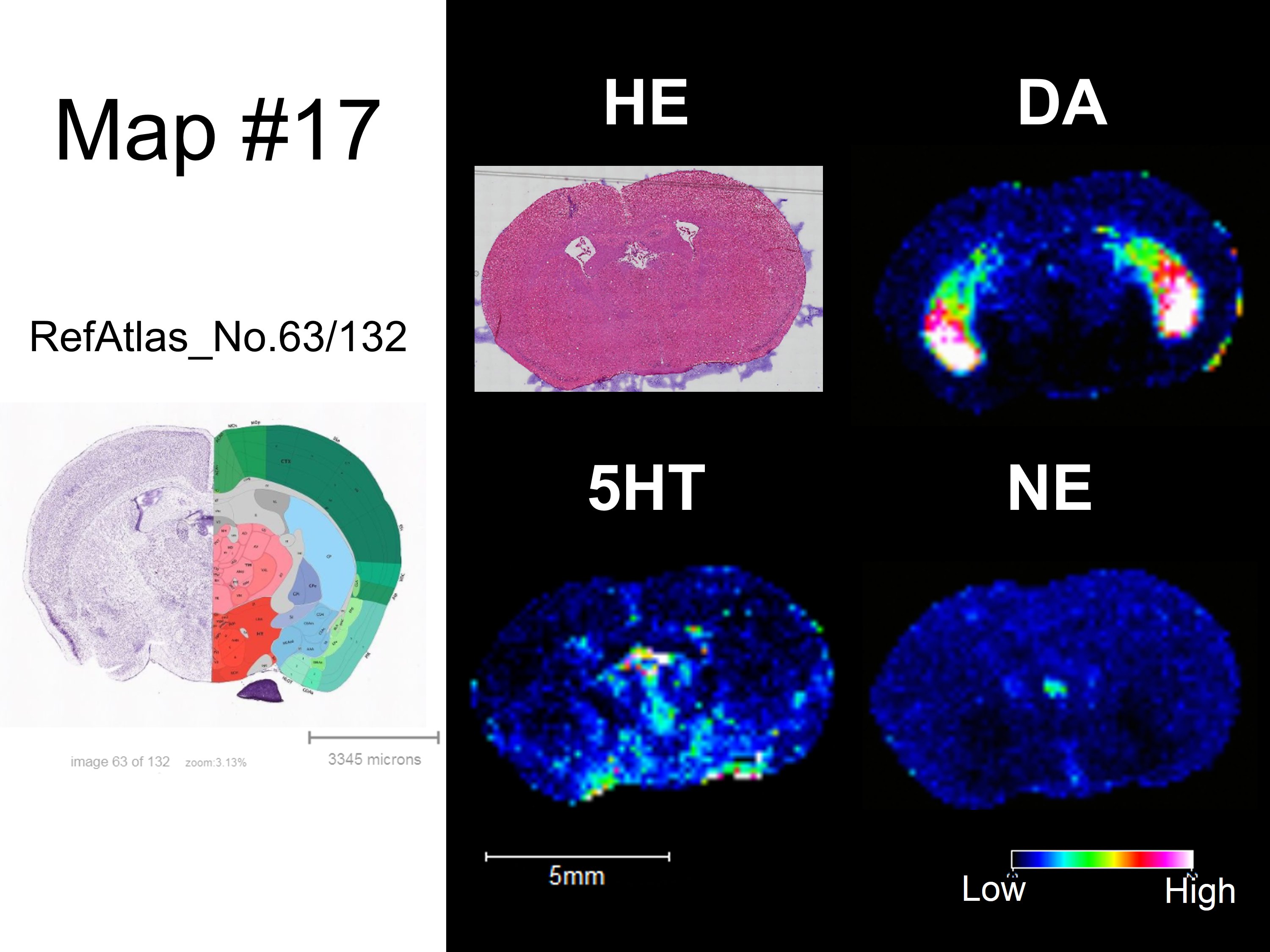

Supplement: Data S1. The Monoamine Atlas of the Mouse Brain, Related to Figure 2A [file mmc2.zip › Data1/âXâëâCâh17.TIF]

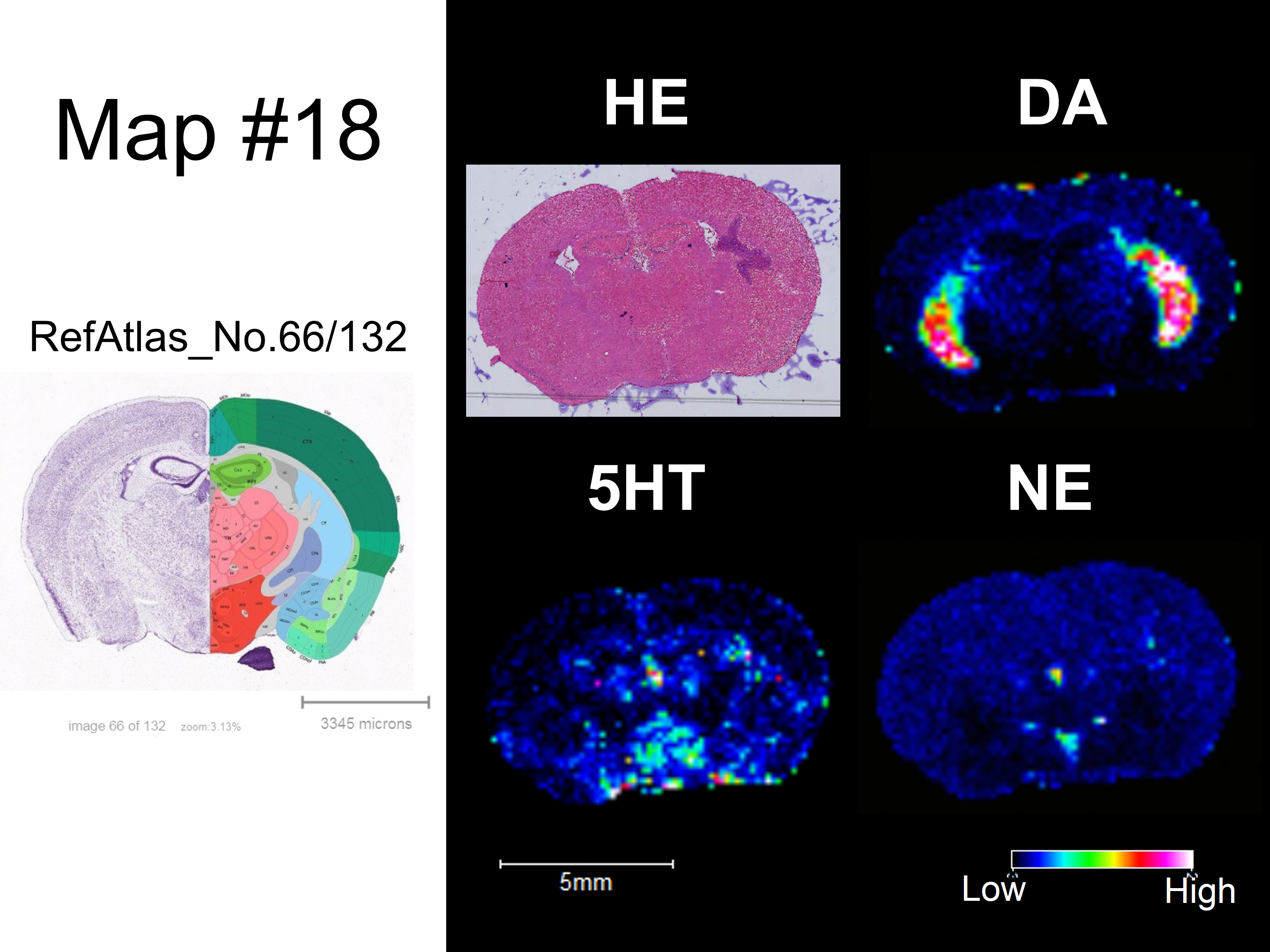

Supplement: Data S1. The Monoamine Atlas of the Mouse Brain, Related to Figure 2A [file mmc2.zip › Data1/âXâëâCâh18.TIF]

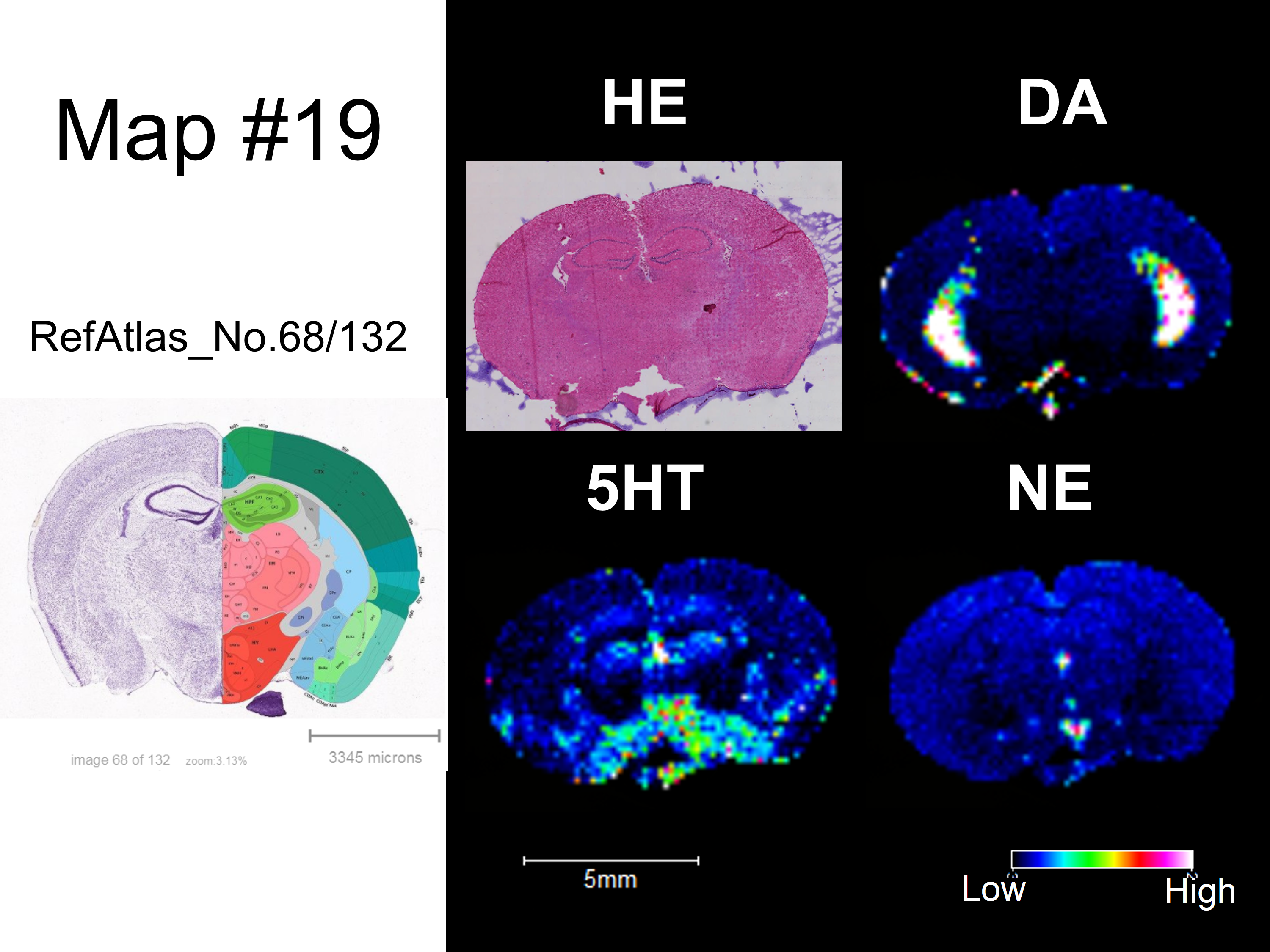

Supplement: Data S1. The Monoamine Atlas of the Mouse Brain, Related to Figure 2A [file mmc2.zip › Data1/âXâëâCâh19.TIF]

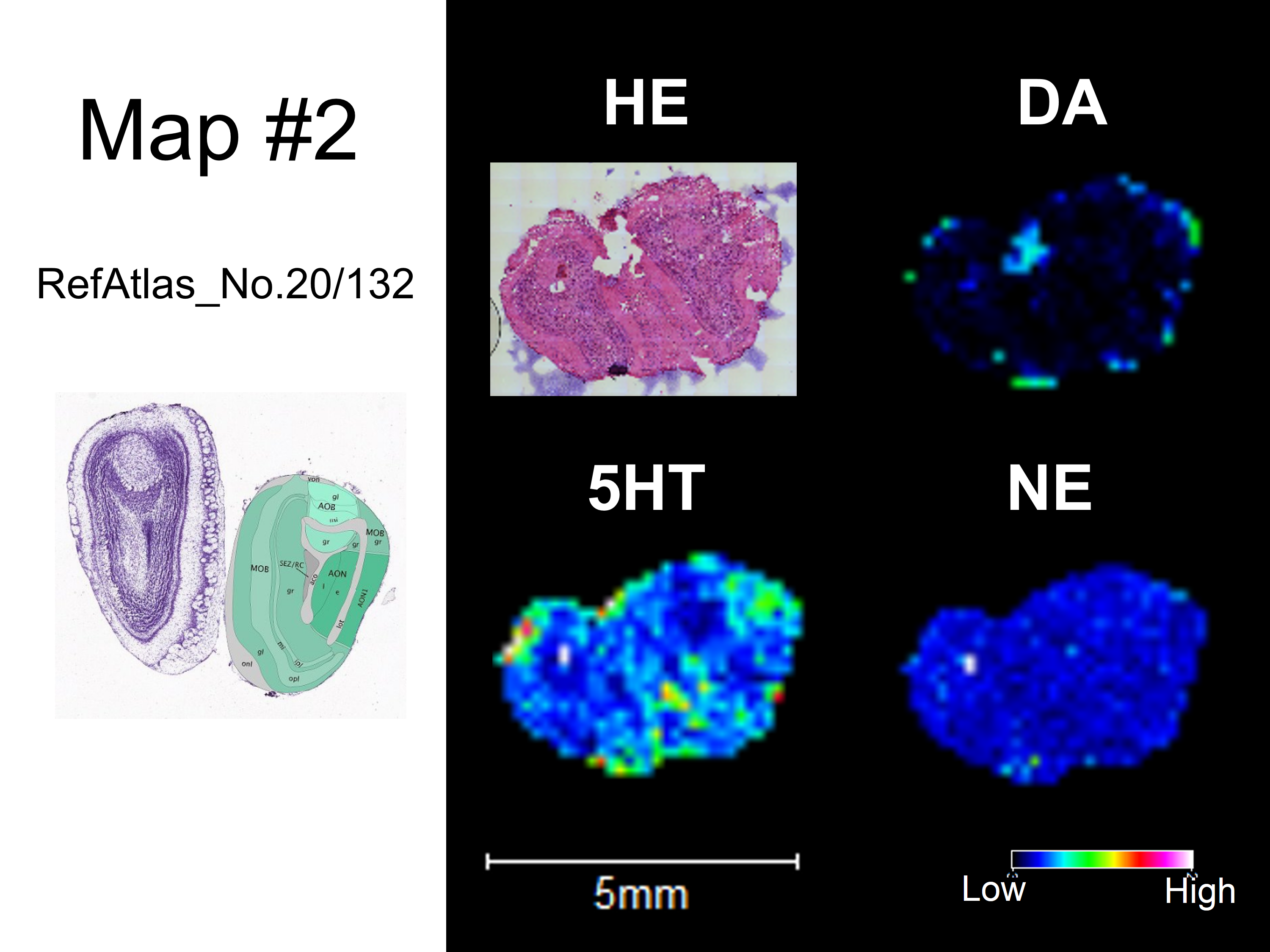

Supplement: Data S1. The Monoamine Atlas of the Mouse Brain, Related to Figure 2A [file mmc2.zip › Data1/âXâëâCâh2.TIF]

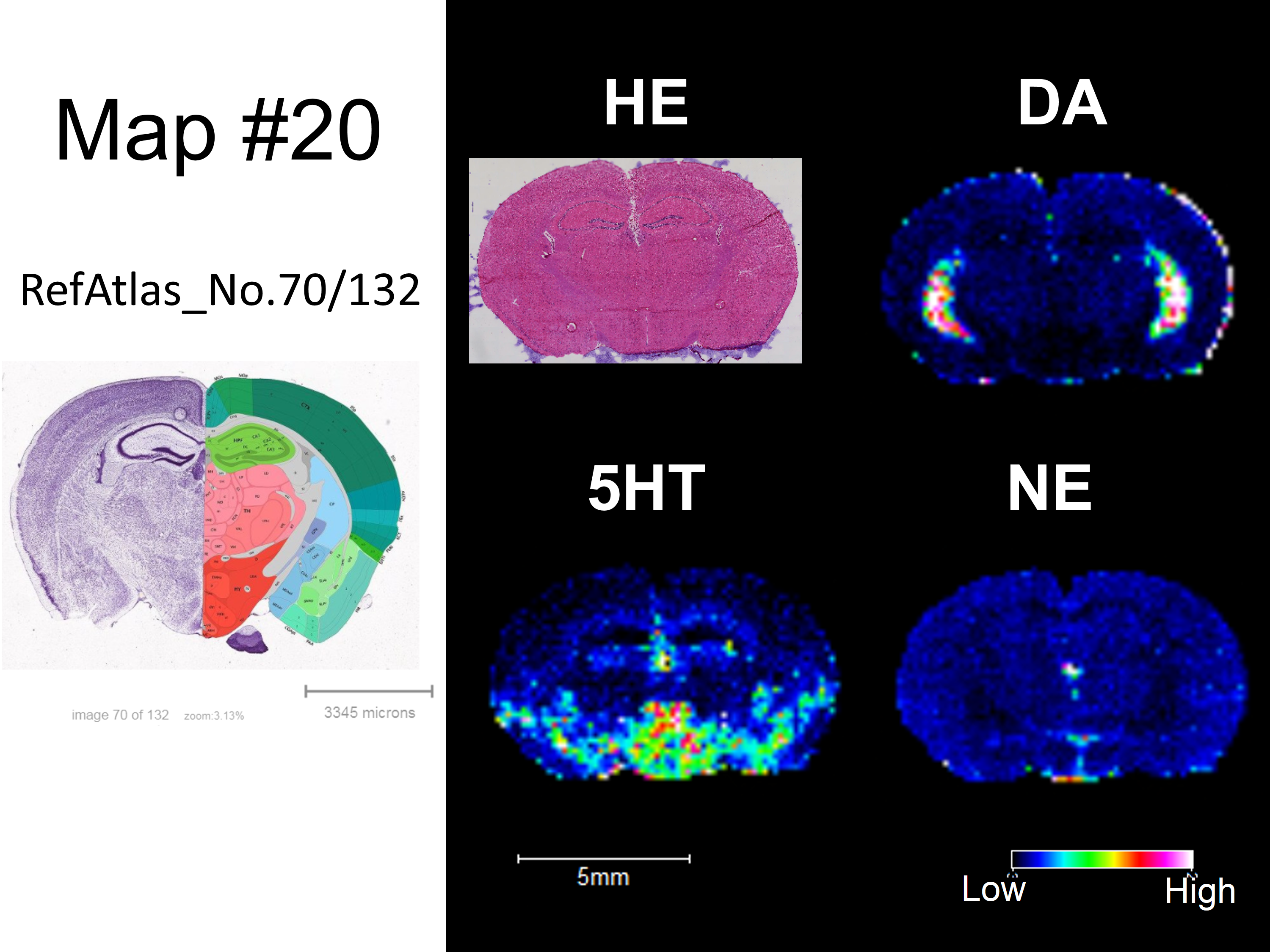

Supplement: Data S1. The Monoamine Atlas of the Mouse Brain, Related to Figure 2A [file mmc2.zip › Data1/âXâëâCâh20.TIF]

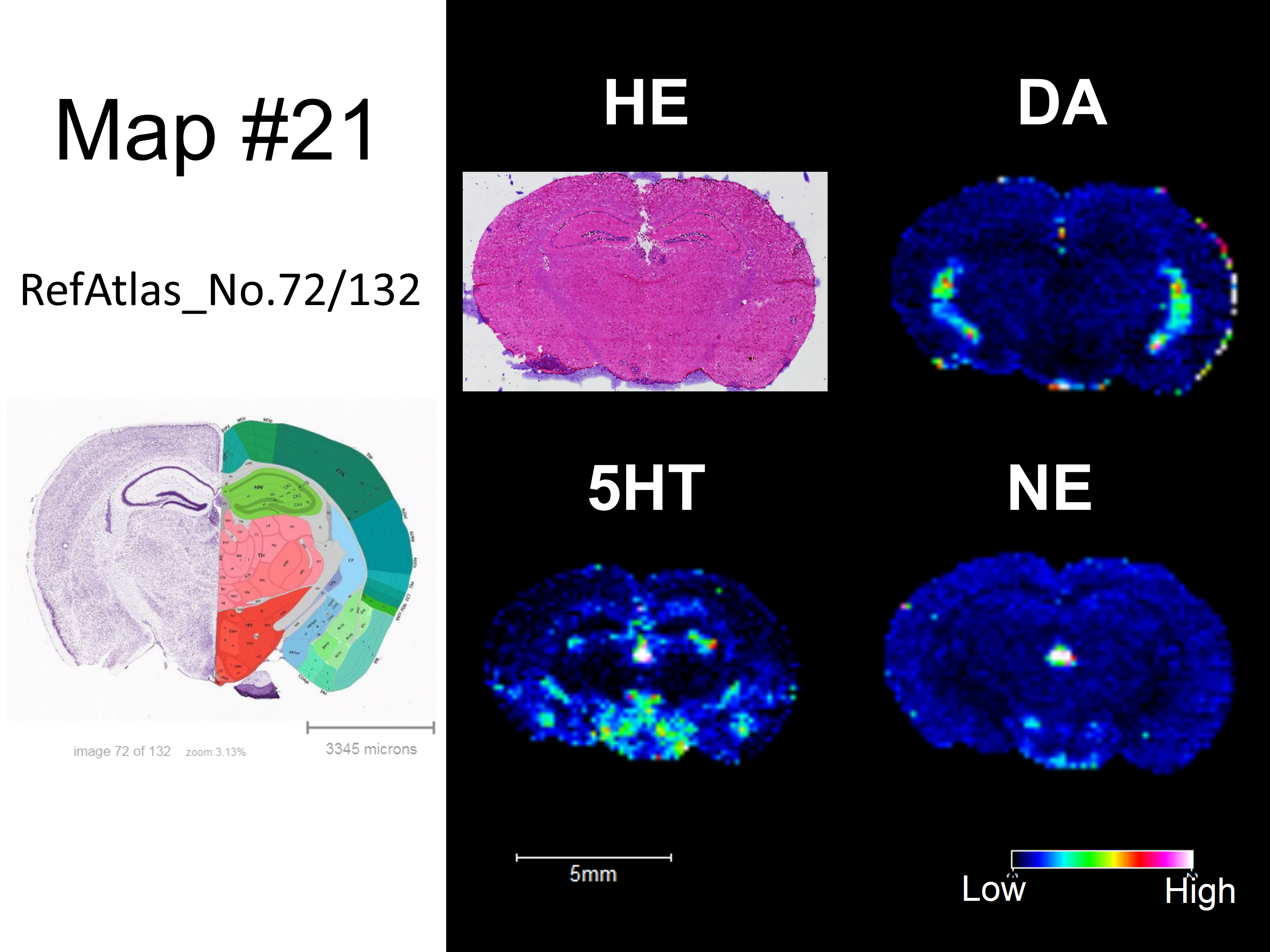

Supplement: Data S1. The Monoamine Atlas of the Mouse Brain, Related to Figure 2A [file mmc2.zip › Data1/âXâëâCâh21.TIF]

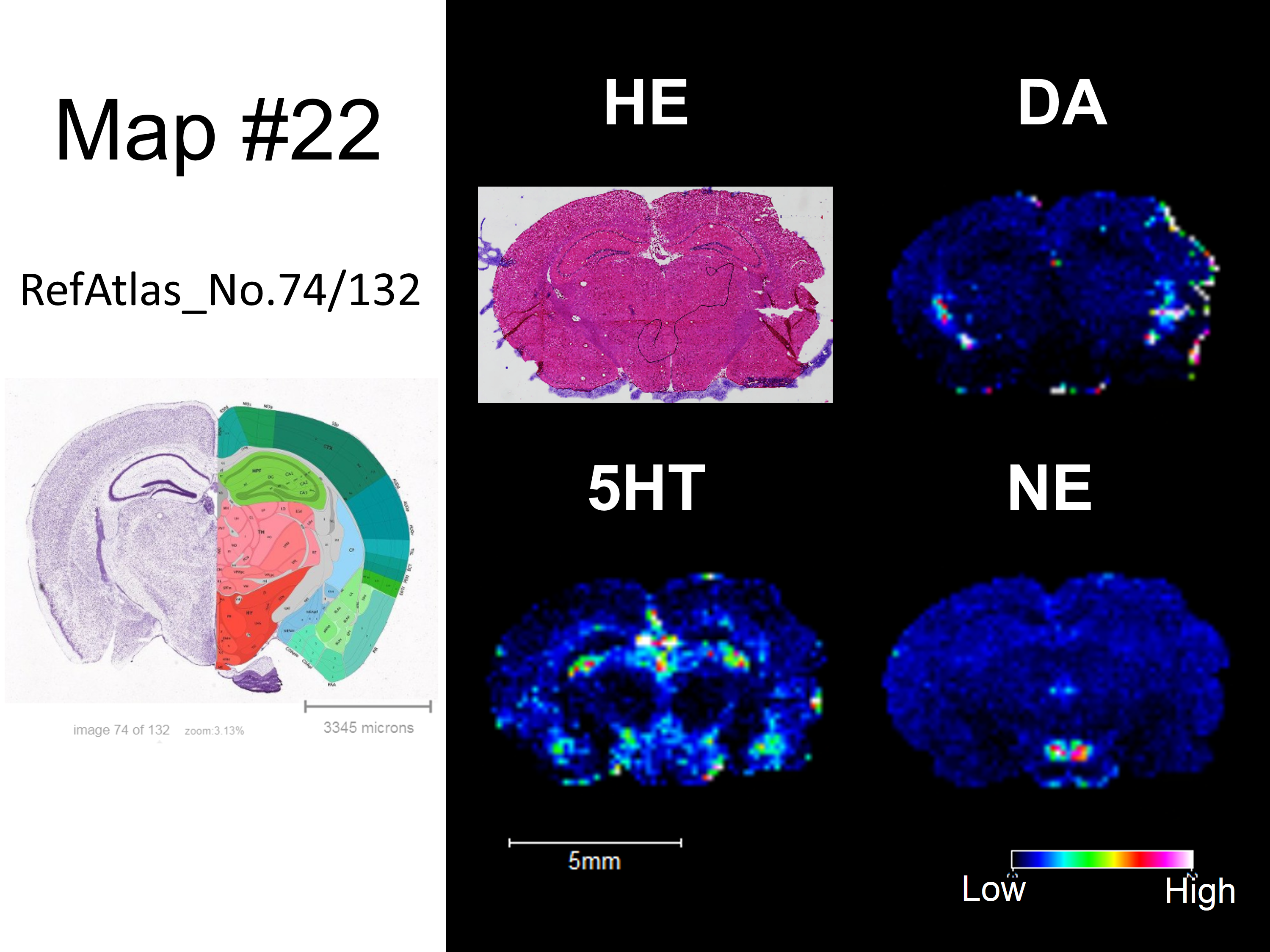

Supplement: Data S1. The Monoamine Atlas of the Mouse Brain, Related to Figure 2A [file mmc2.zip › Data1/âXâëâCâh22.TIF]

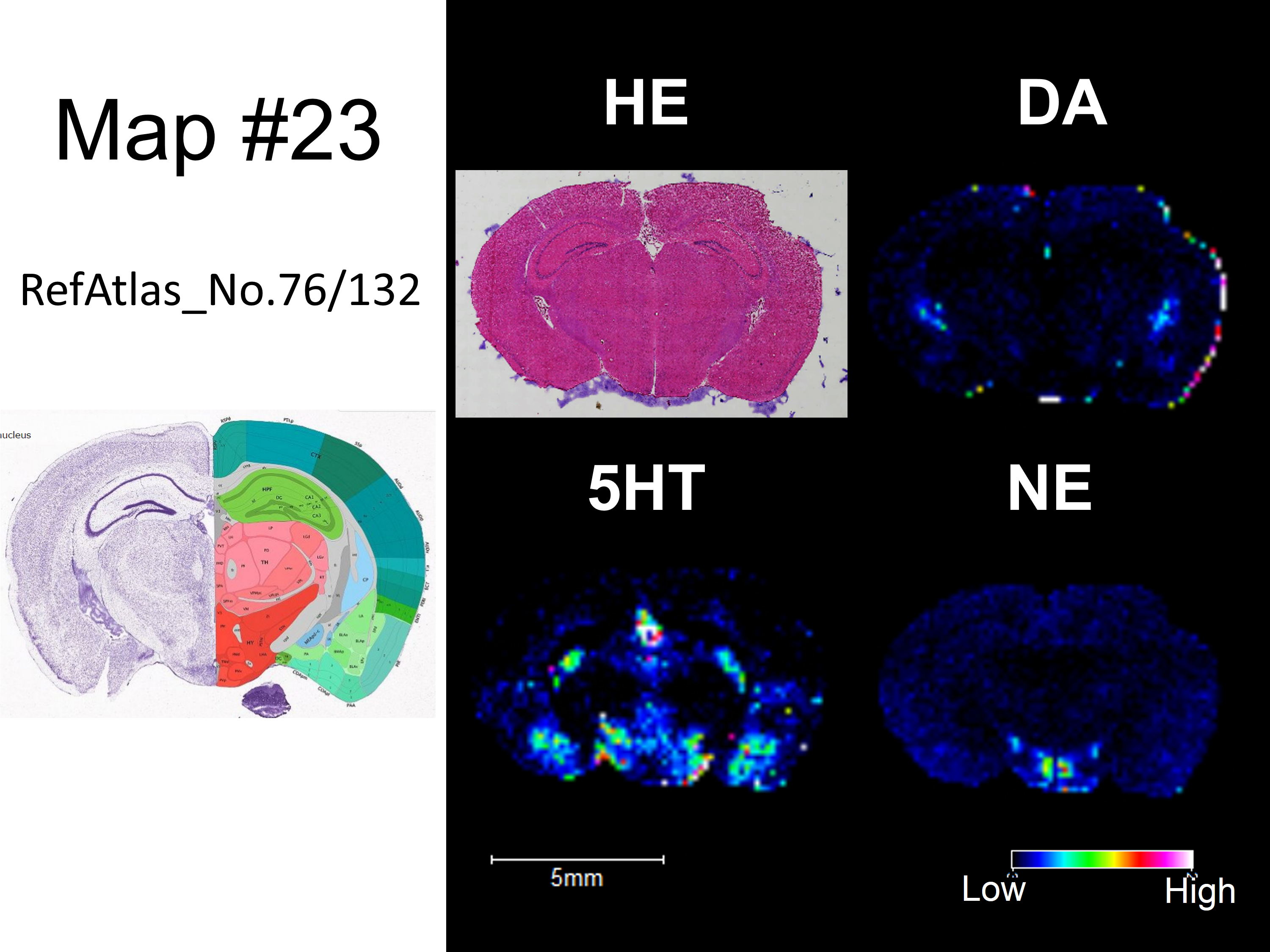

Supplement: Data S1. The Monoamine Atlas of the Mouse Brain, Related to Figure 2A [file mmc2.zip › Data1/âXâëâCâh23.TIF]

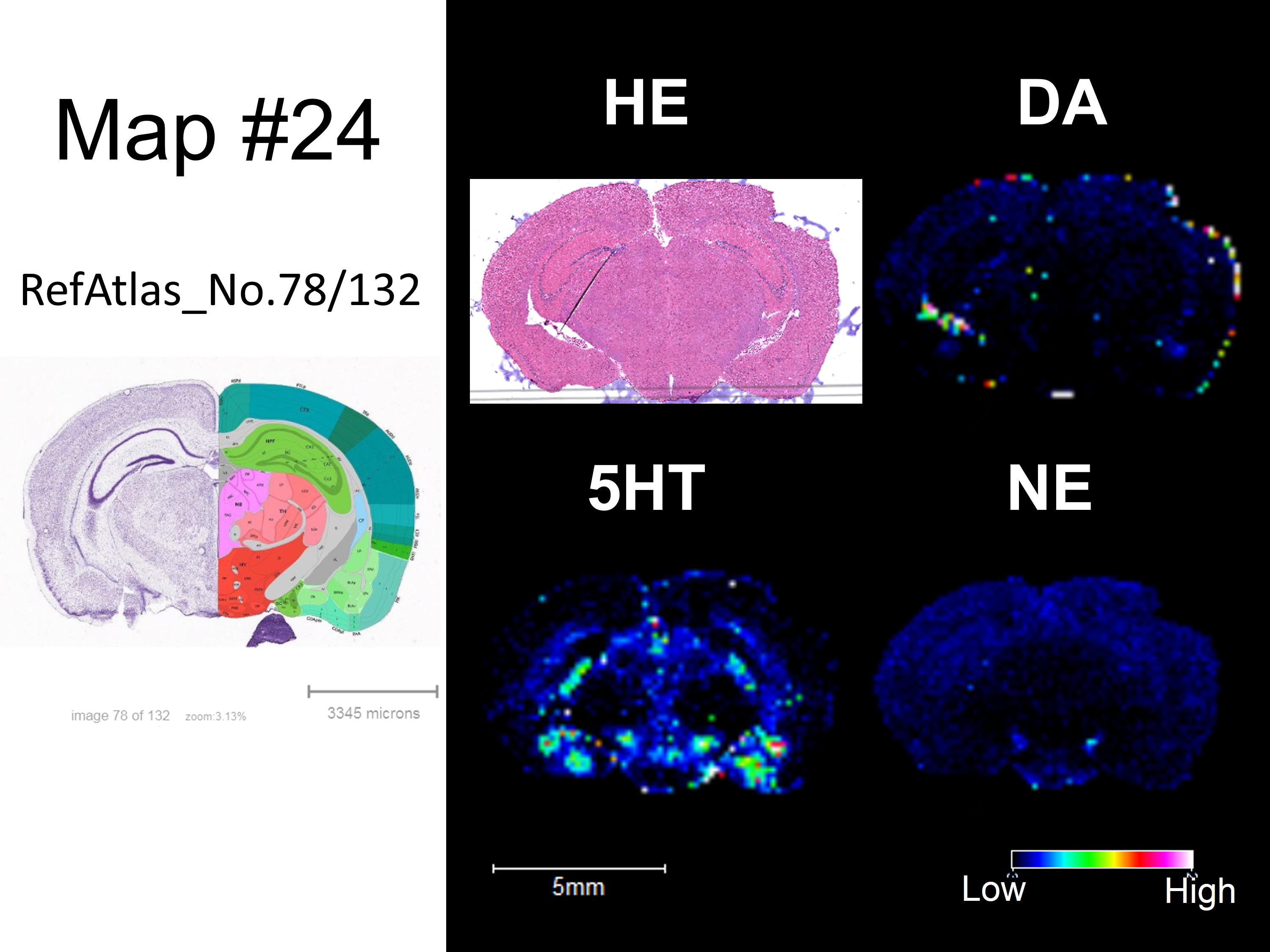

Supplement: Data S1. The Monoamine Atlas of the Mouse Brain, Related to Figure 2A [file mmc2.zip › Data1/âXâëâCâh24.TIF]

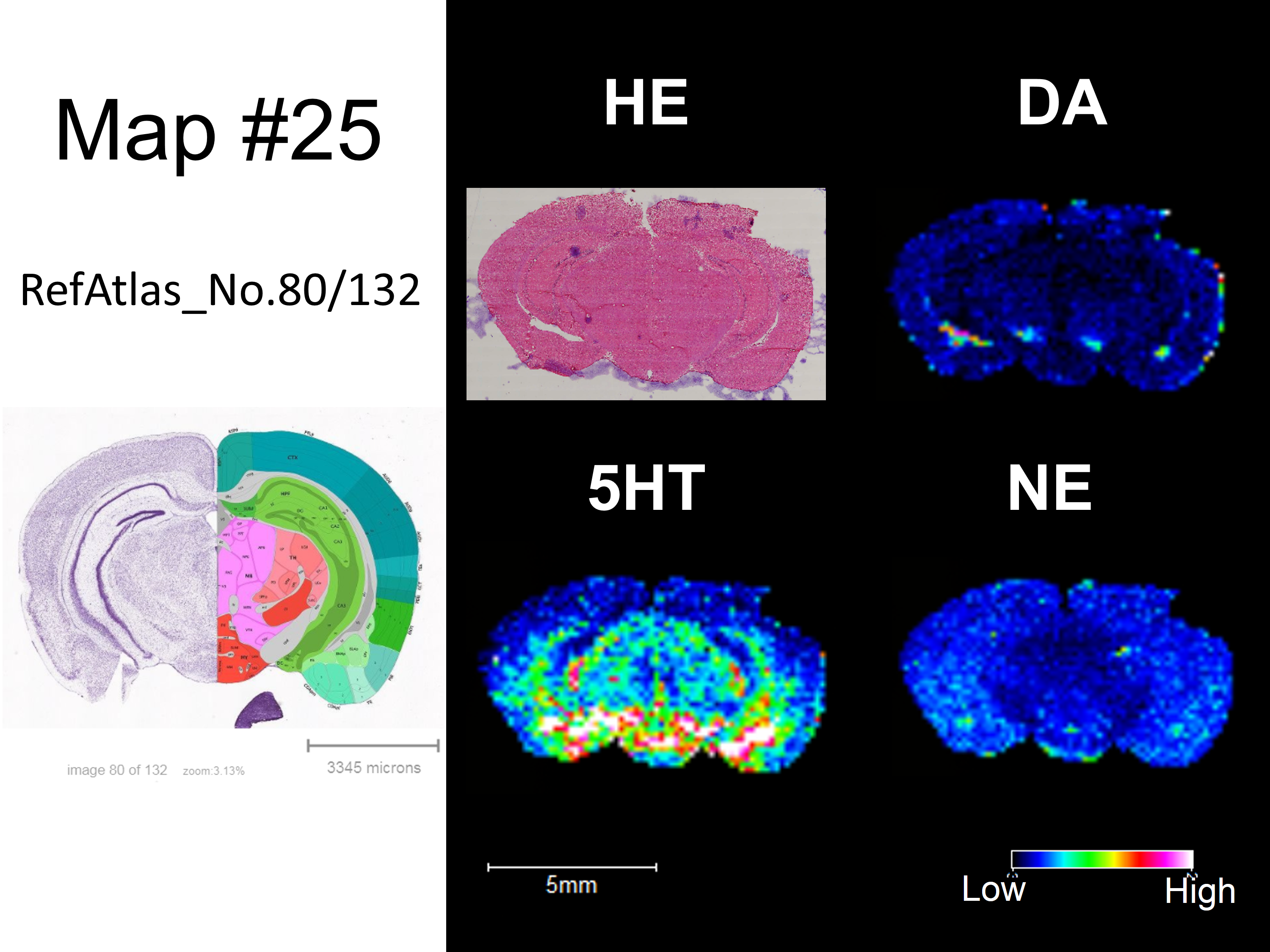

Supplement: Data S1. The Monoamine Atlas of the Mouse Brain, Related to Figure 2A [file mmc2.zip › Data1/âXâëâCâh25.TIF]

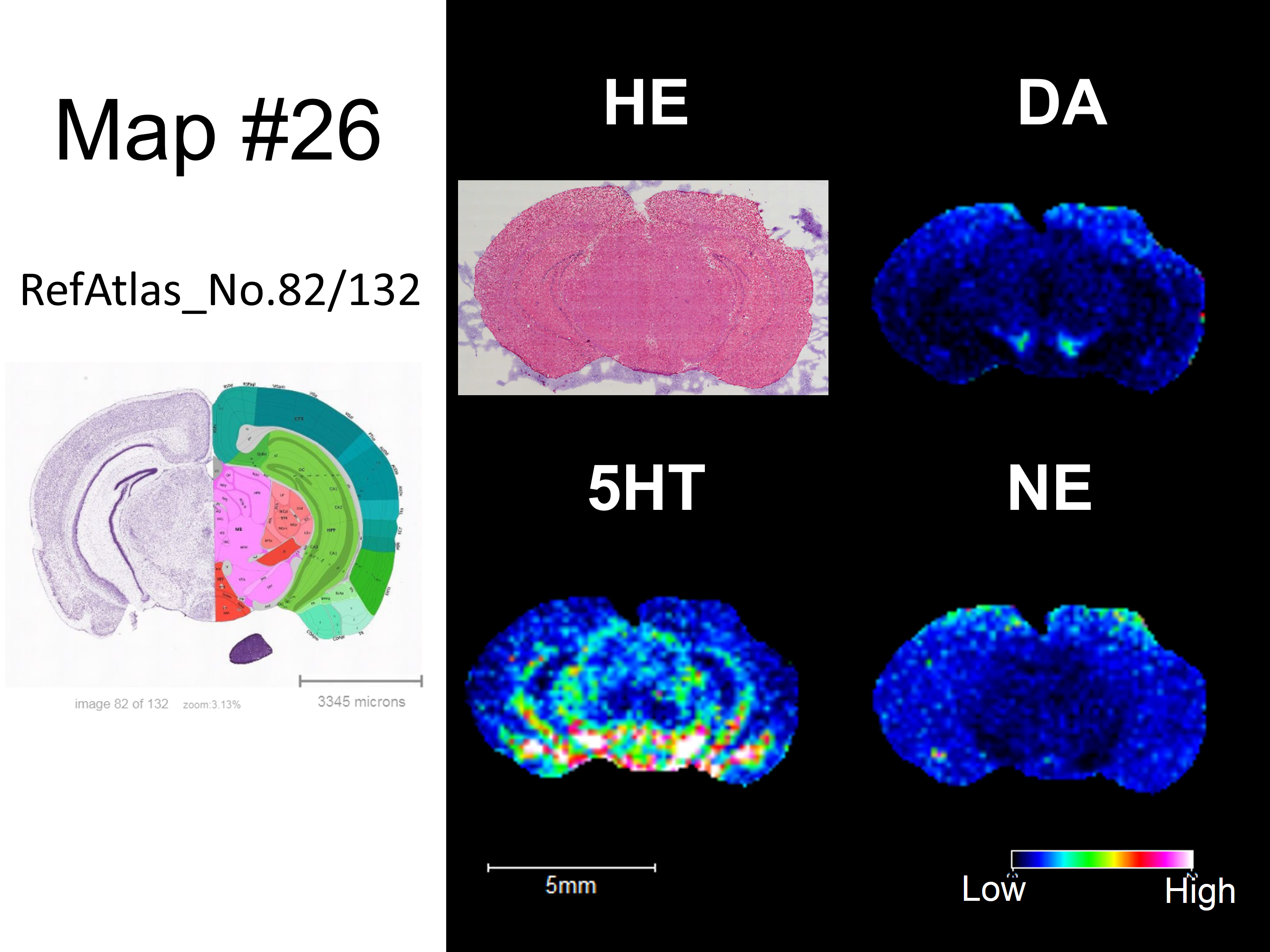

Supplement: Data S1. The Monoamine Atlas of the Mouse Brain, Related to Figure 2A [file mmc2.zip › Data1/âXâëâCâh26.TIF]

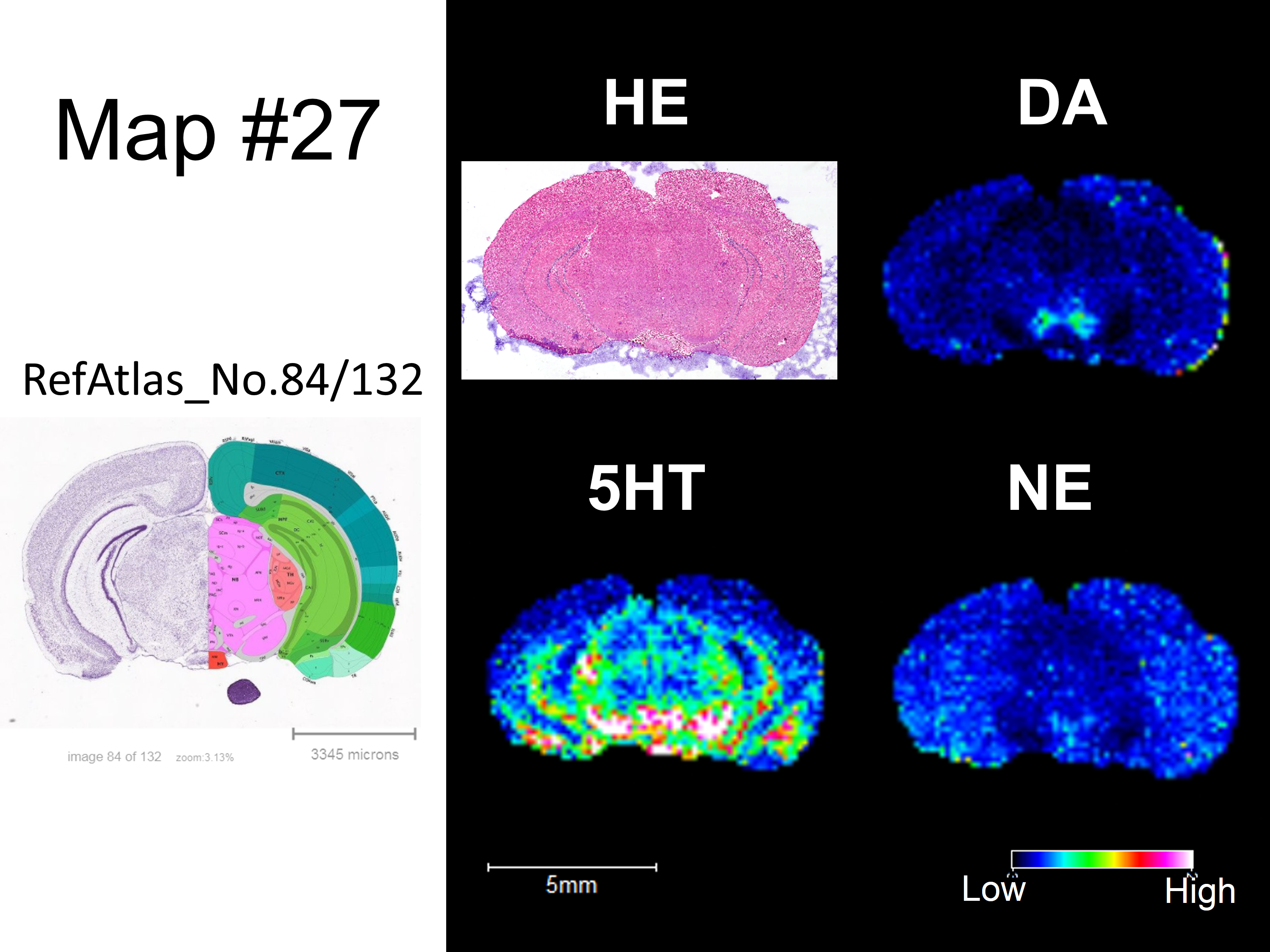

Supplement: Data S1. The Monoamine Atlas of the Mouse Brain, Related to Figure 2A [file mmc2.zip › Data1/âXâëâCâh27.TIF]

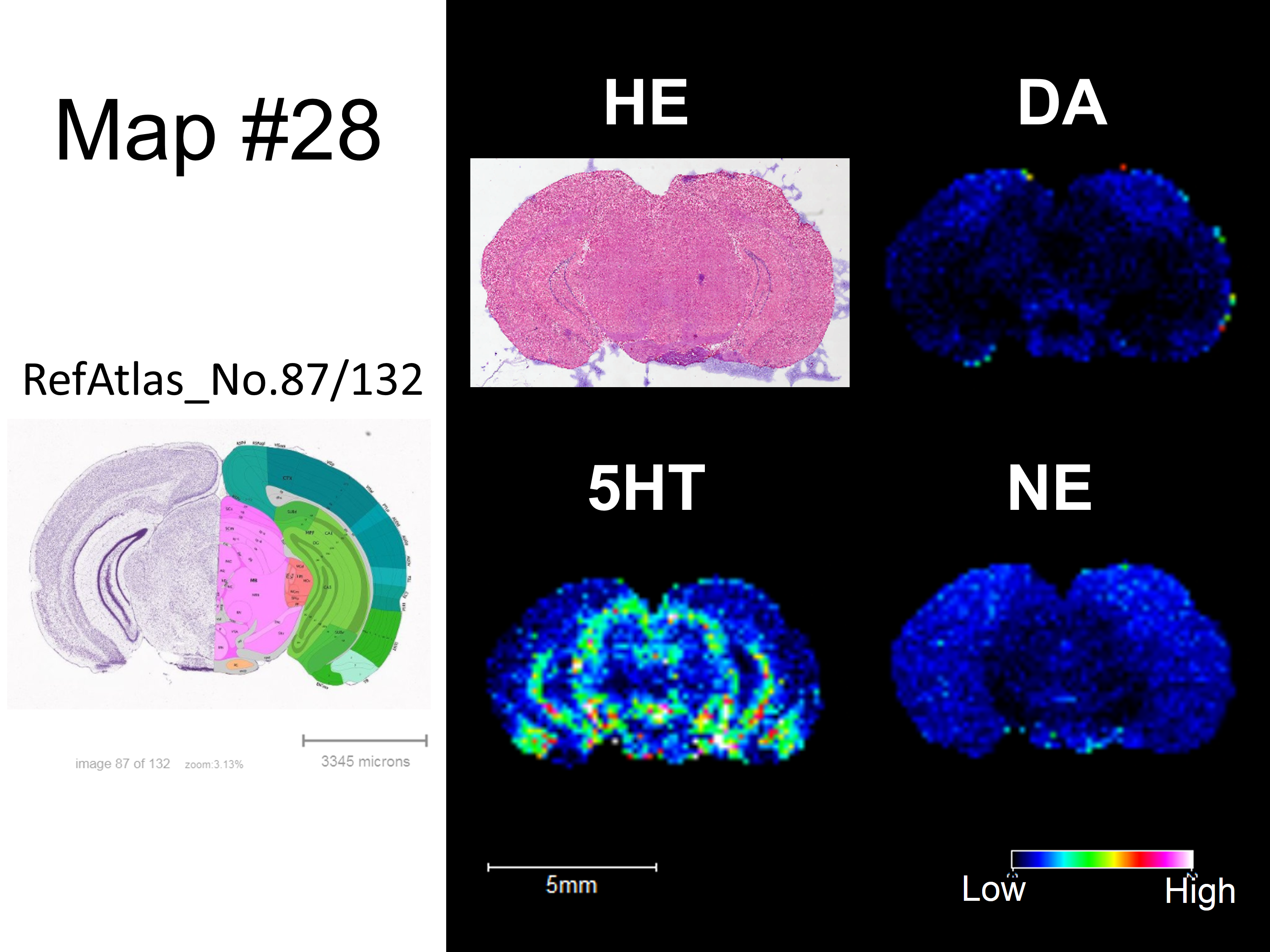

Supplement: Data S1. The Monoamine Atlas of the Mouse Brain, Related to Figure 2A [file mmc2.zip › Data1/âXâëâCâh28.TIF]

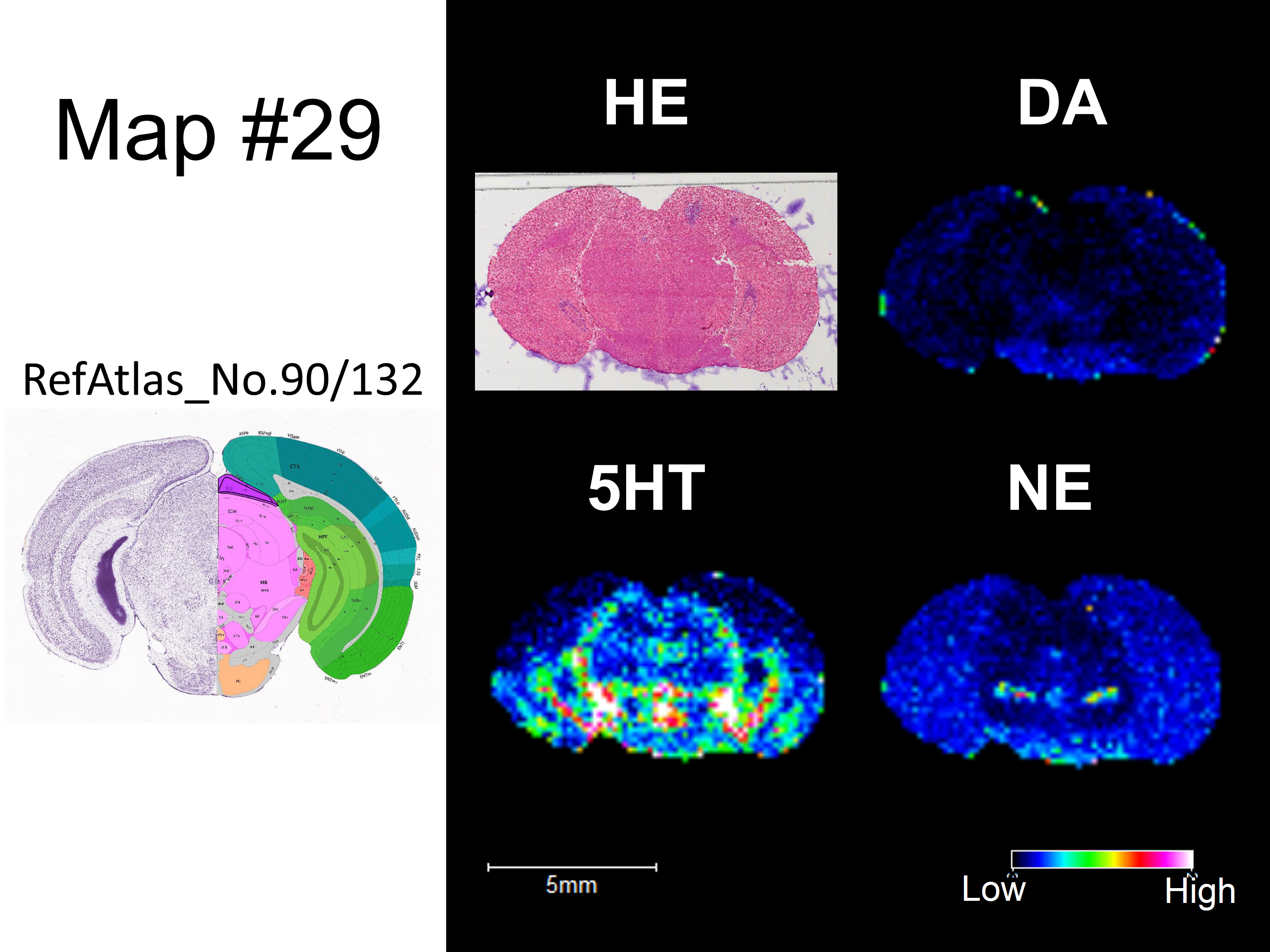

Supplement: Data S1. The Monoamine Atlas of the Mouse Brain, Related to Figure 2A [file mmc2.zip › Data1/âXâëâCâh29.TIF]

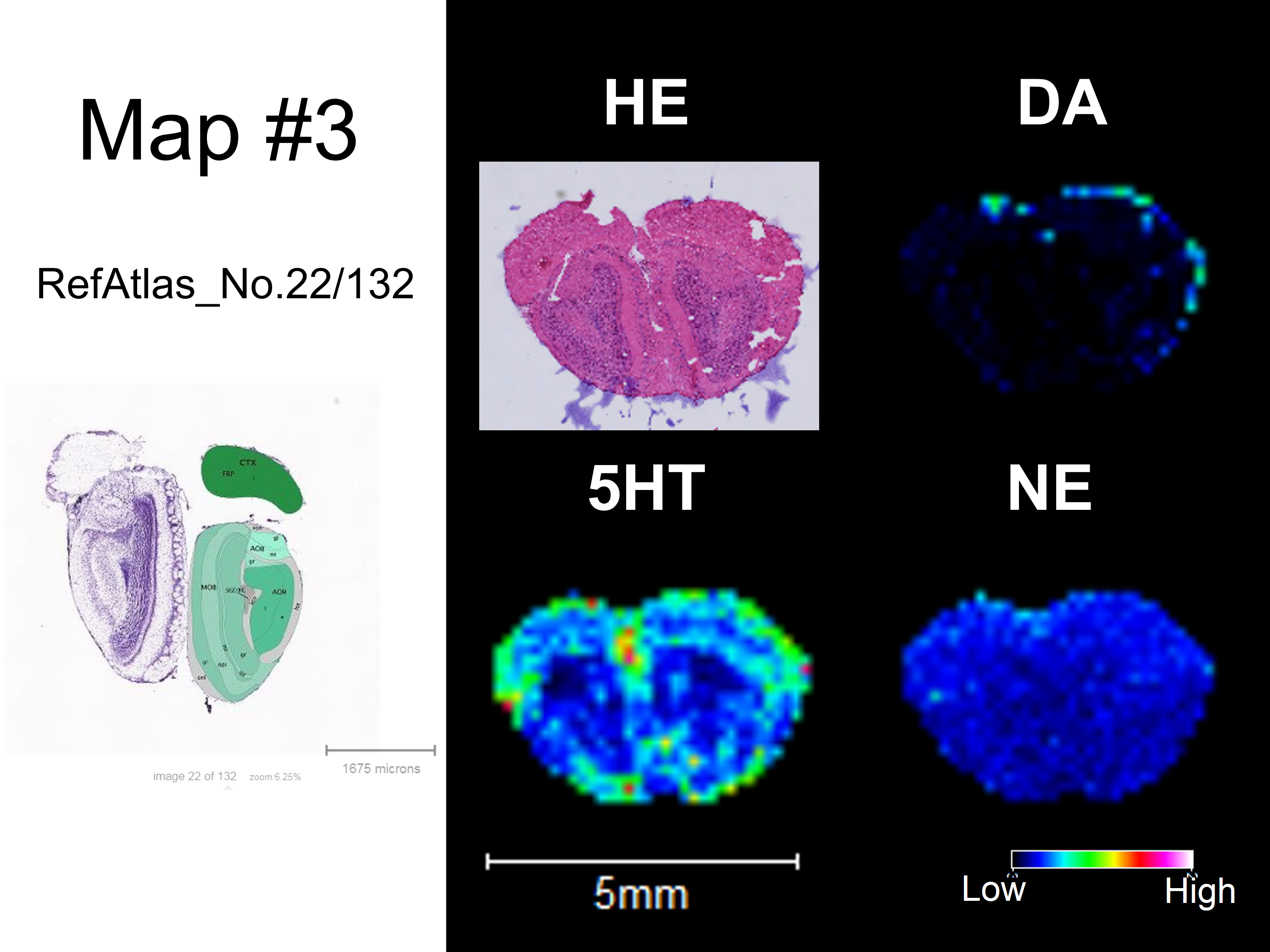

Supplement: Data S1. The Monoamine Atlas of the Mouse Brain, Related to Figure 2A [file mmc2.zip › Data1/âXâëâCâh3.TIF]

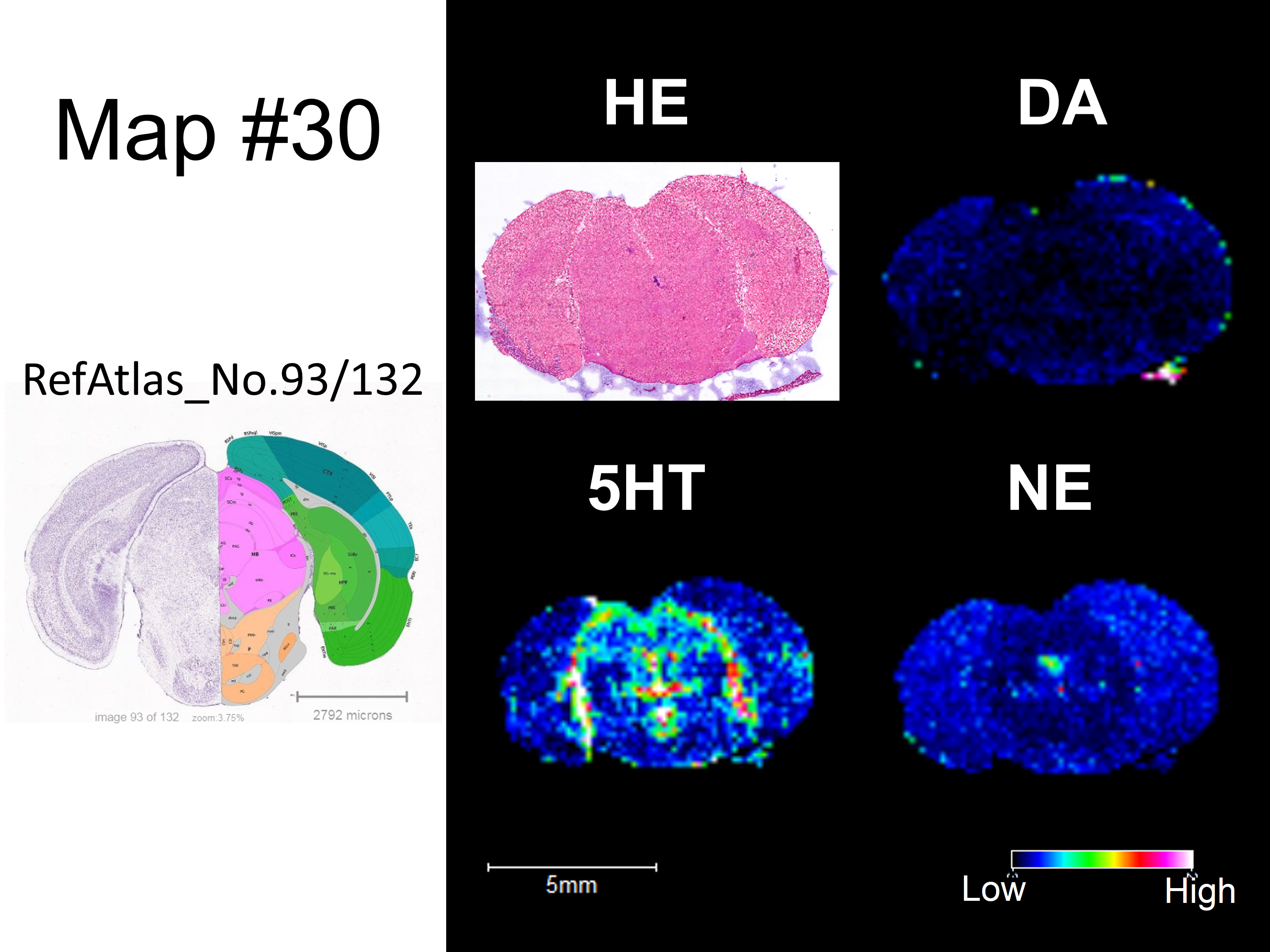

Supplement: Data S1. The Monoamine Atlas of the Mouse Brain, Related to Figure 2A [file mmc2.zip › Data1/âXâëâCâh30.TIF]

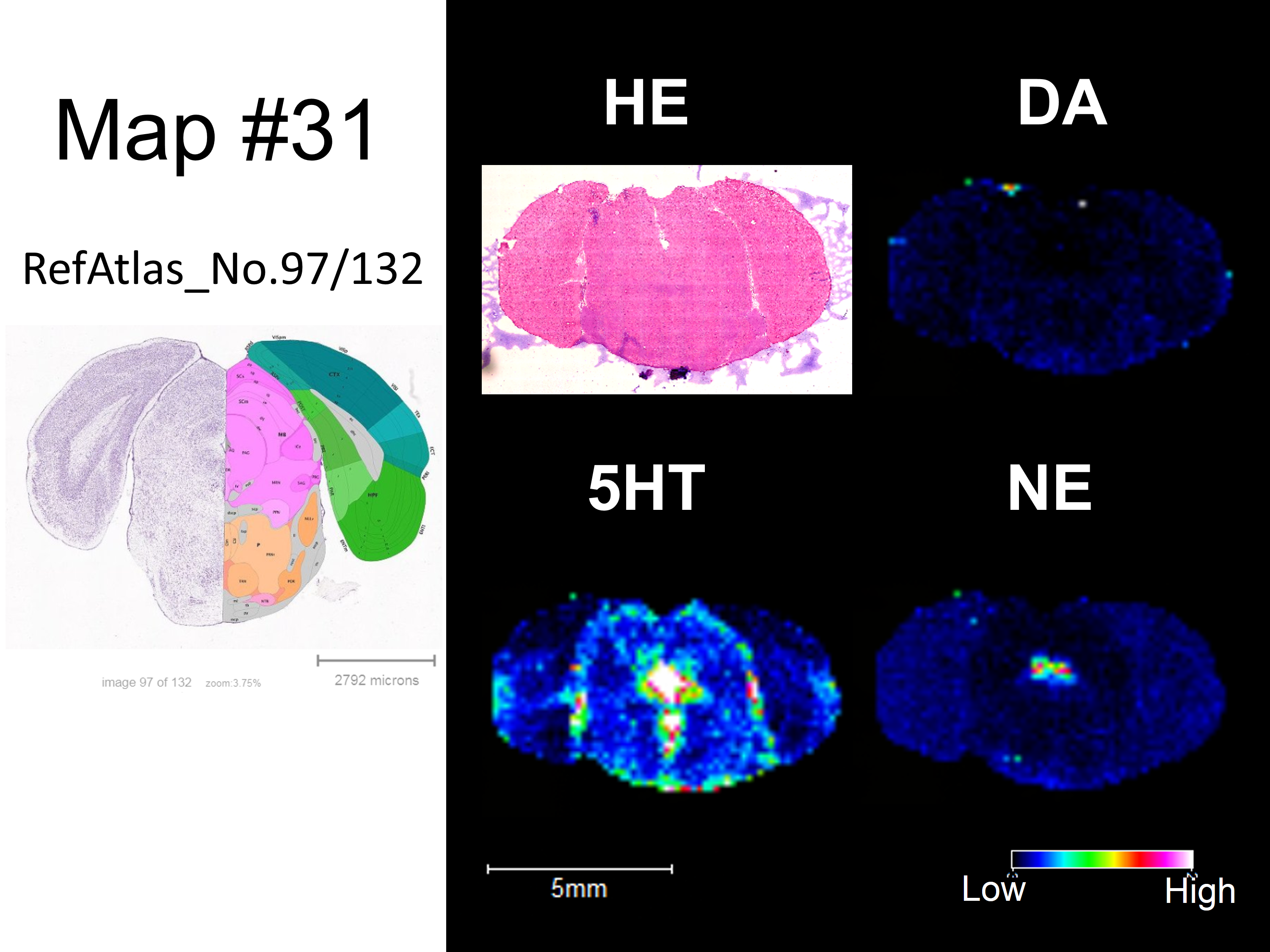

Supplement: Data S1. The Monoamine Atlas of the Mouse Brain, Related to Figure 2A [file mmc2.zip › Data1/âXâëâCâh31.TIF]

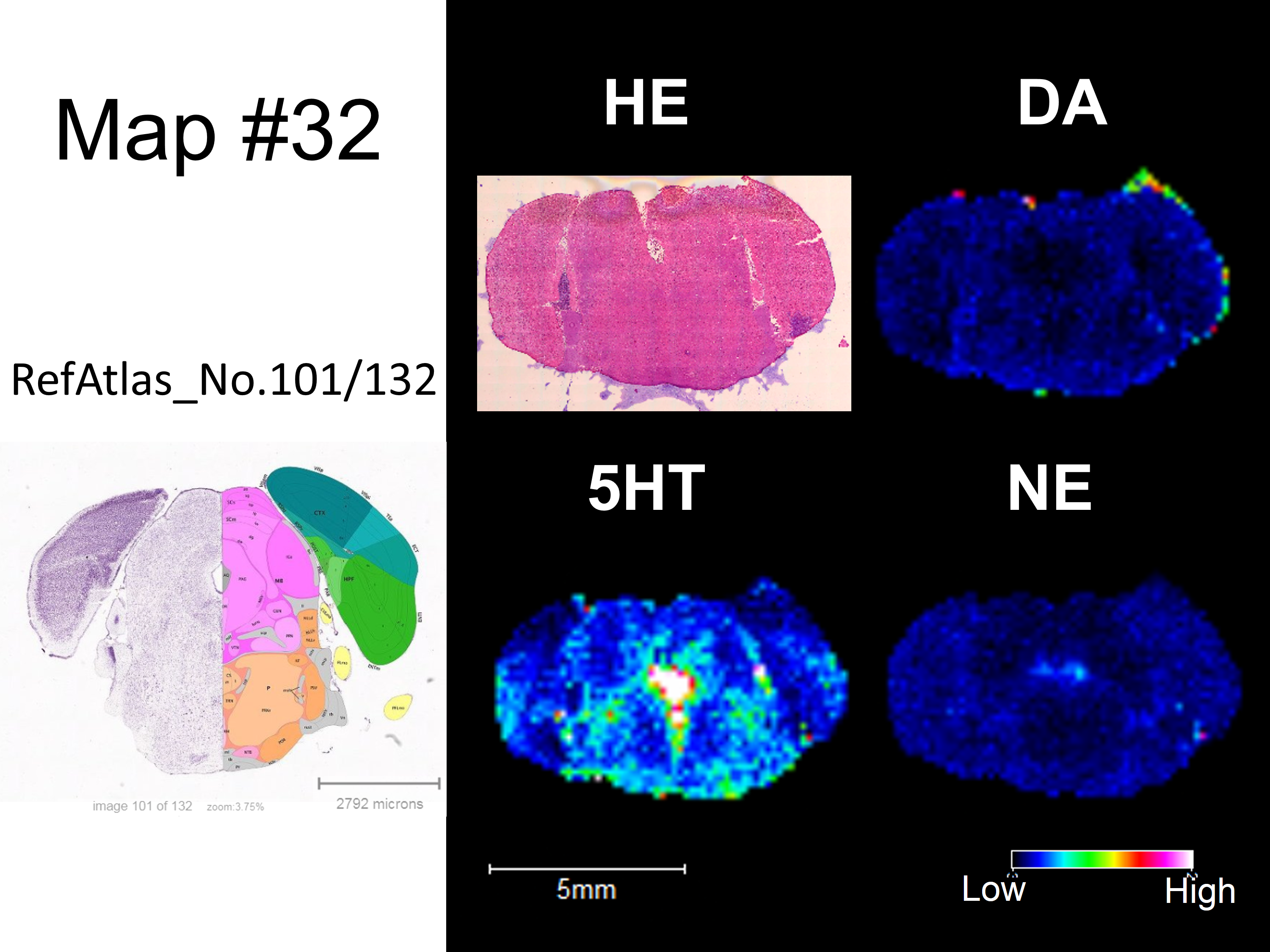

Supplement: Data S1. The Monoamine Atlas of the Mouse Brain, Related to Figure 2A [file mmc2.zip › Data1/âXâëâCâh32.TIF]

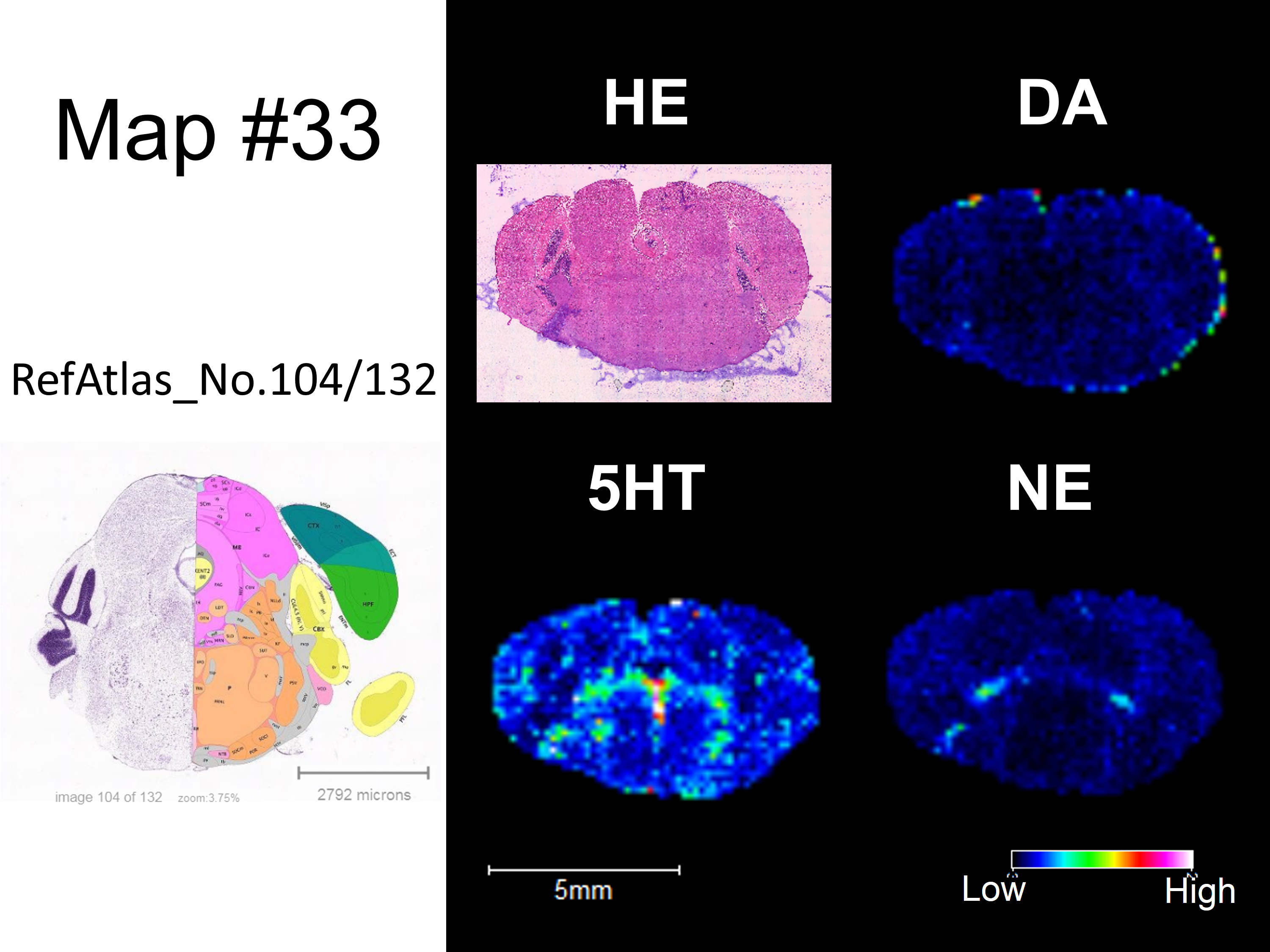

Supplement: Data S1. The Monoamine Atlas of the Mouse Brain, Related to Figure 2A [file mmc2.zip › Data1/âXâëâCâh33.TIF]

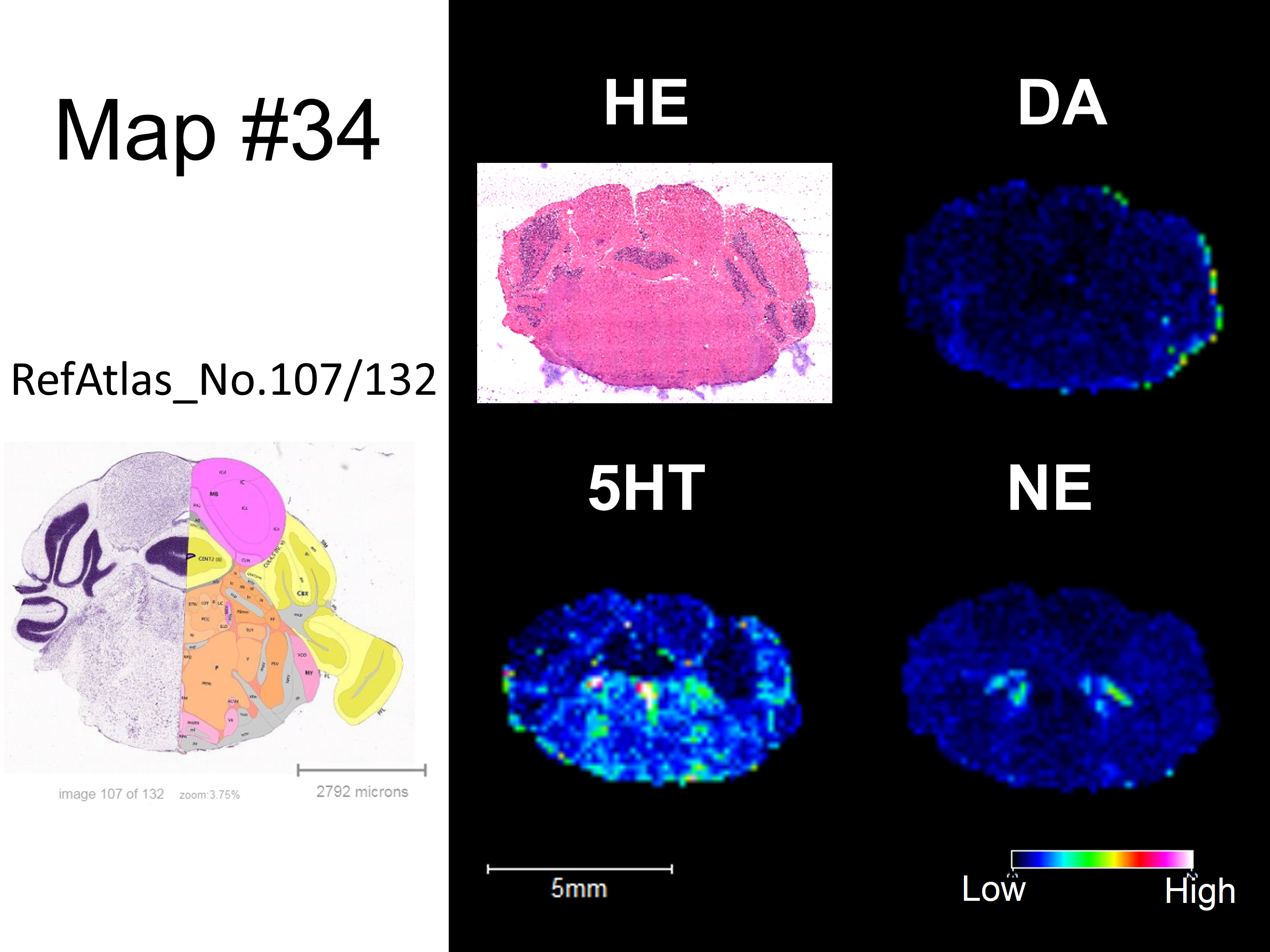

Supplement: Data S1. The Monoamine Atlas of the Mouse Brain, Related to Figure 2A [file mmc2.zip › Data1/âXâëâCâh34.TIF]

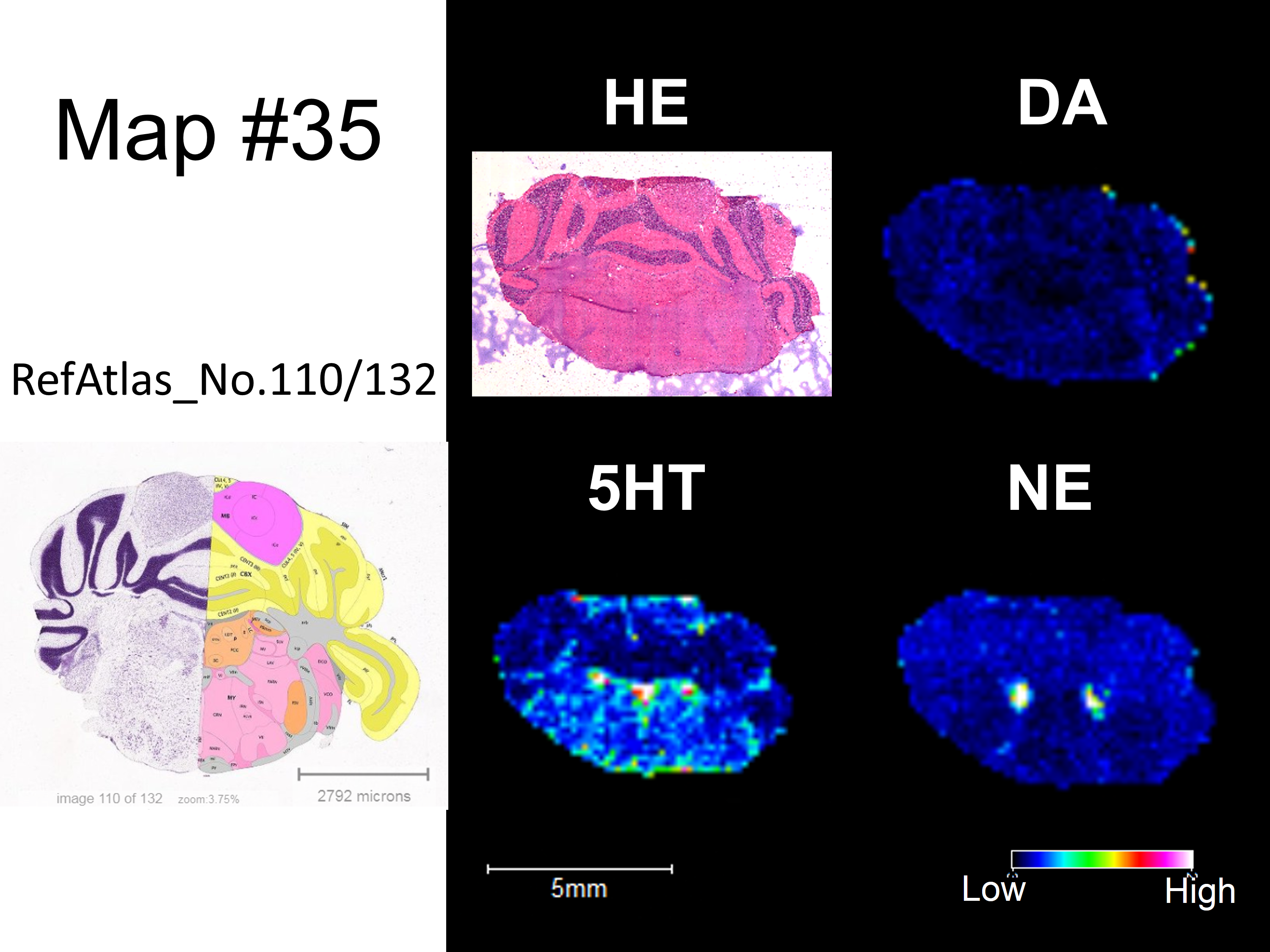

Supplement: Data S1. The Monoamine Atlas of the Mouse Brain, Related to Figure 2A [file mmc2.zip › Data1/âXâëâCâh35.TIF]

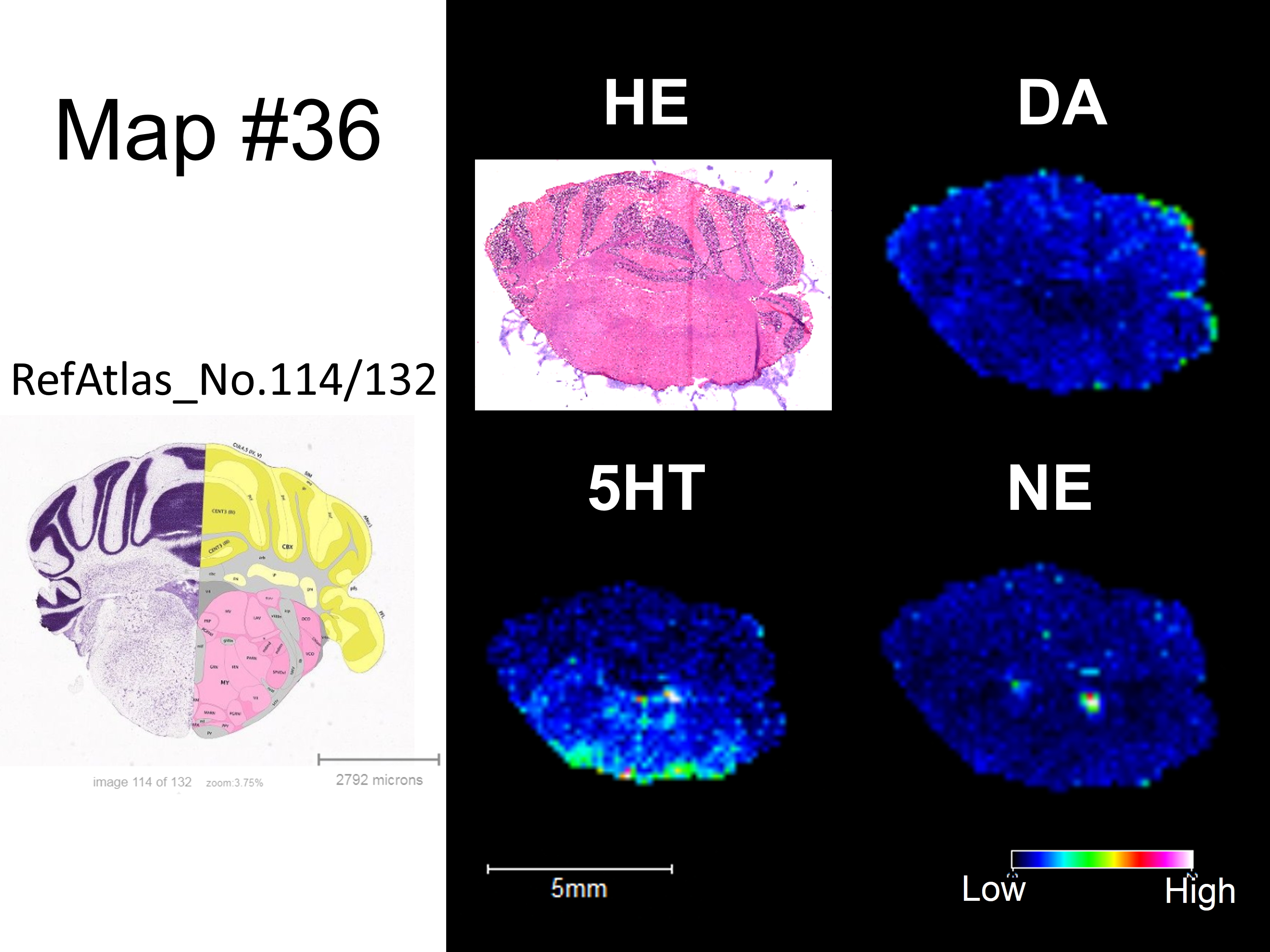

Supplement: Data S1. The Monoamine Atlas of the Mouse Brain, Related to Figure 2A [file mmc2.zip › Data1/âXâëâCâh36.TIF]

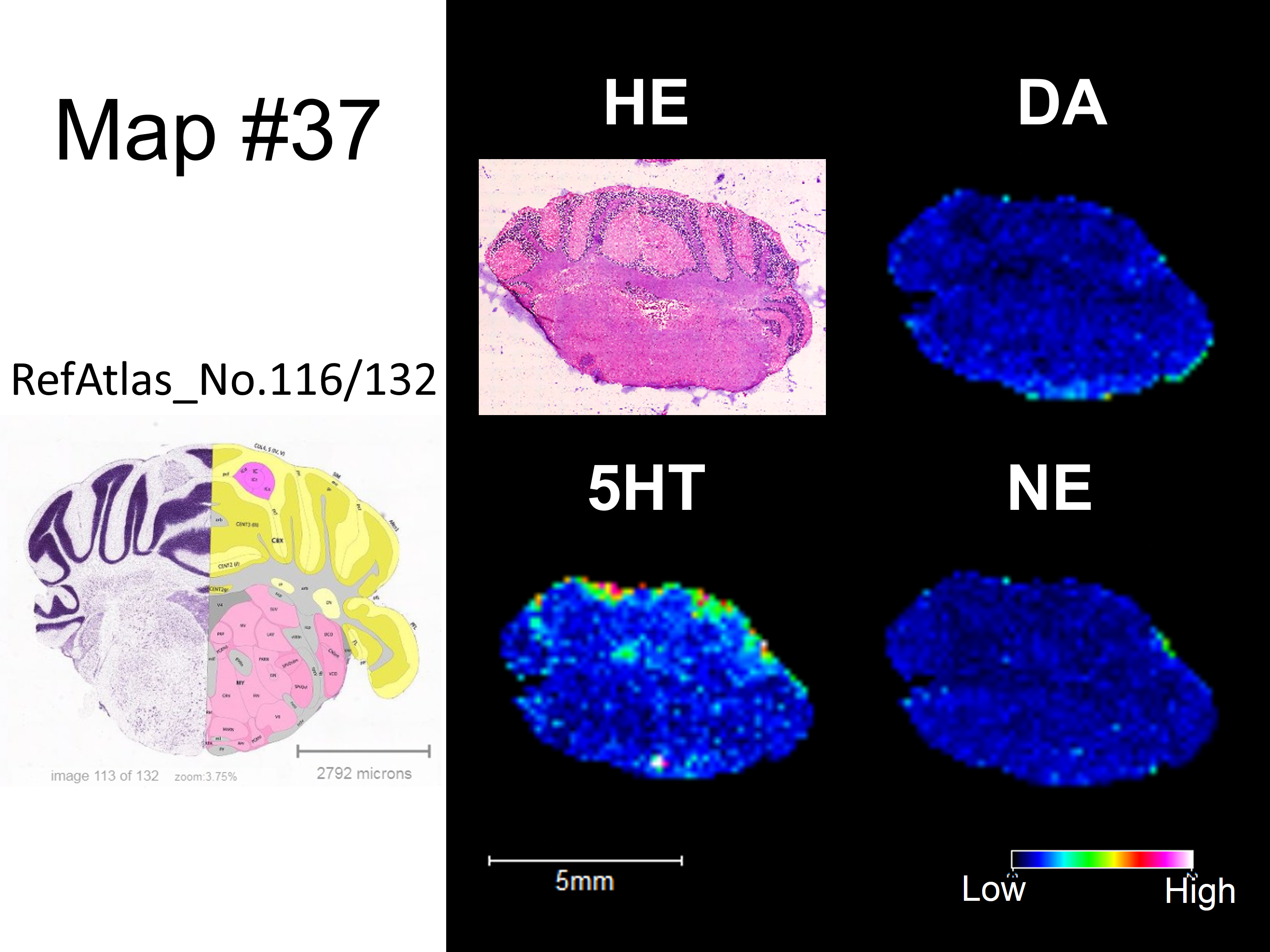

Supplement: Data S1. The Monoamine Atlas of the Mouse Brain, Related to Figure 2A [file mmc2.zip › Data1/âXâëâCâh37.TIF]

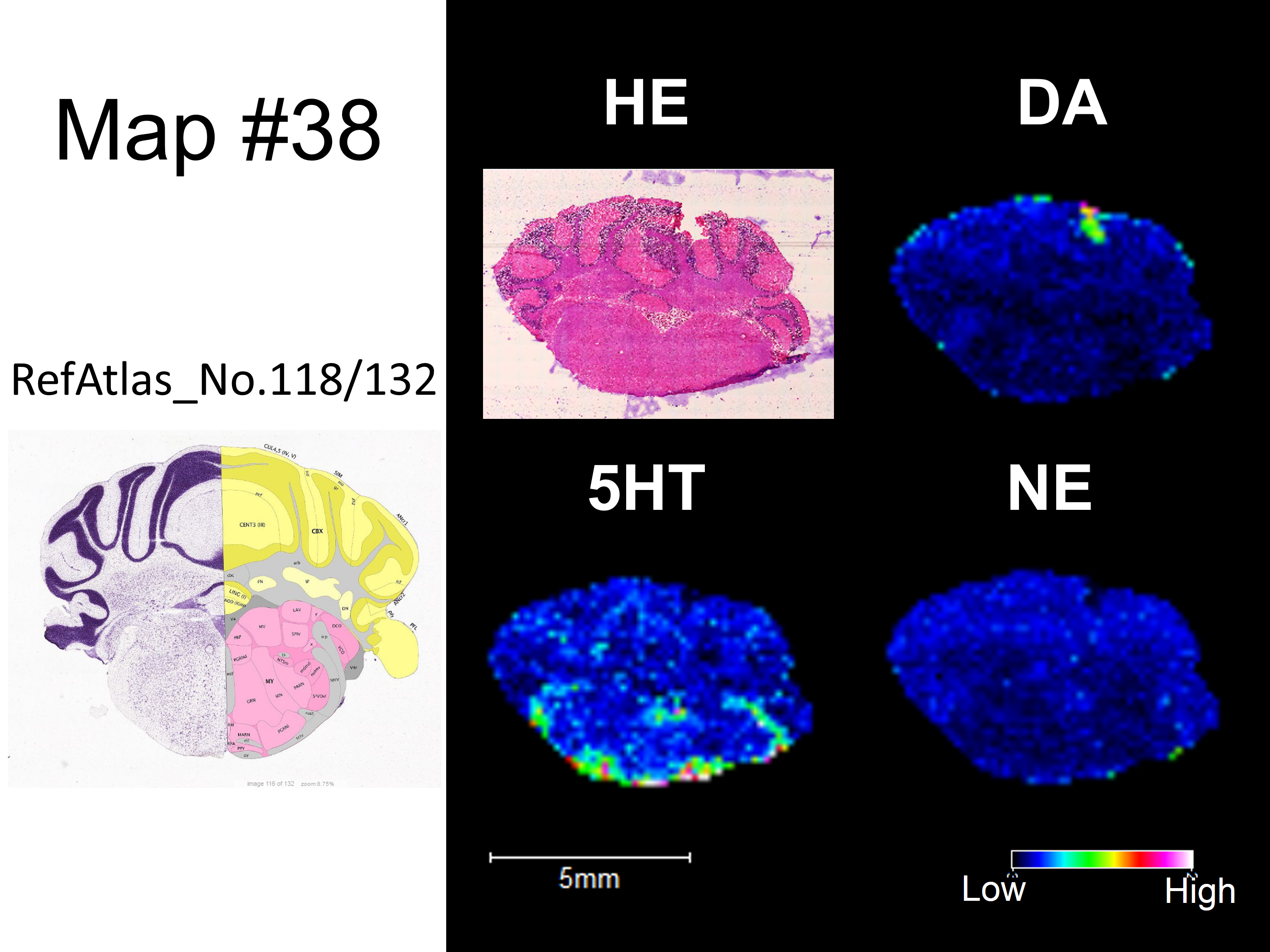

Supplement: Data S1. The Monoamine Atlas of the Mouse Brain, Related to Figure 2A [file mmc2.zip › Data1/âXâëâCâh38.TIF]

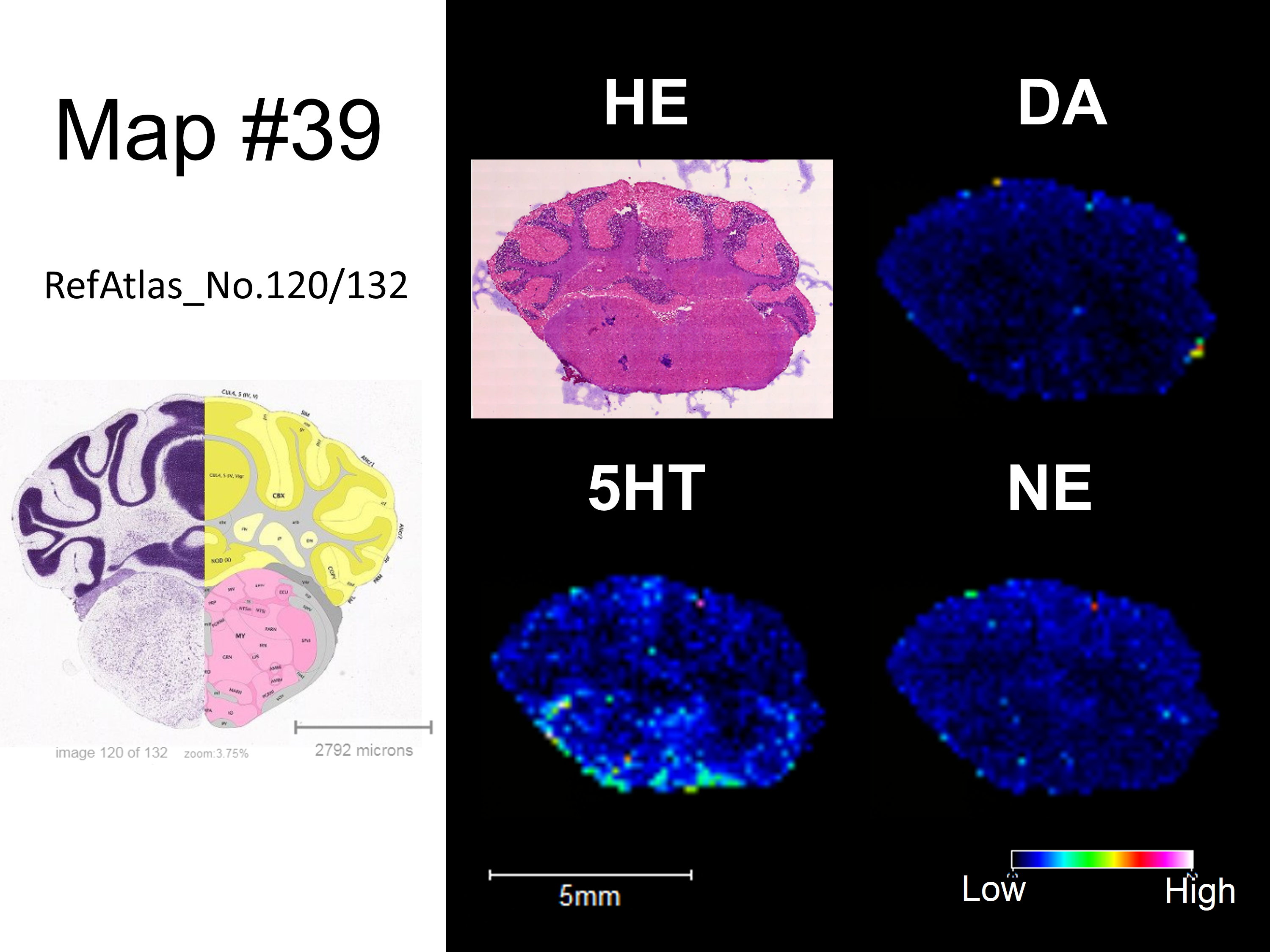

Supplement: Data S1. The Monoamine Atlas of the Mouse Brain, Related to Figure 2A [file mmc2.zip › Data1/âXâëâCâh39.TIF]

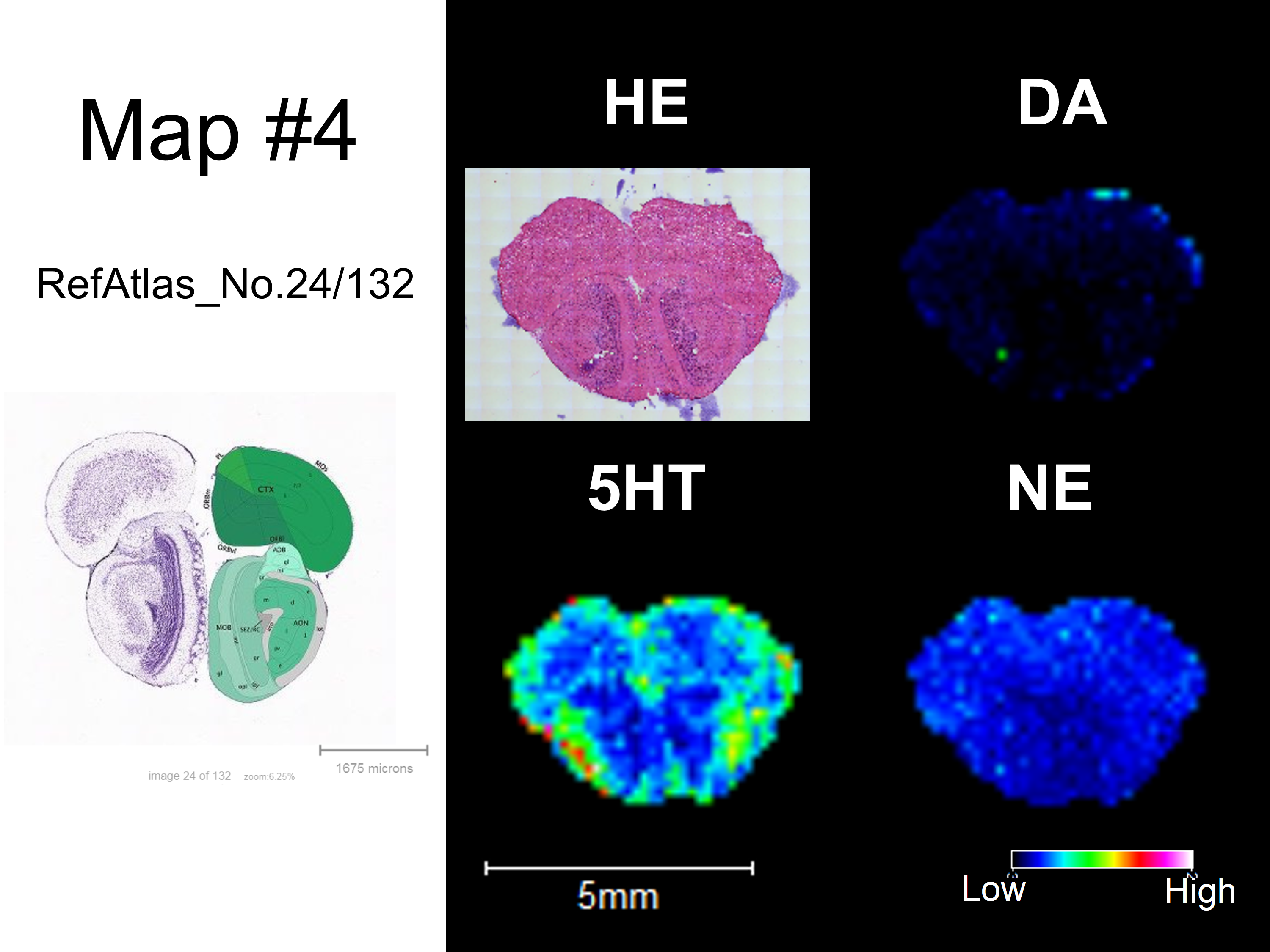

Supplement: Data S1. The Monoamine Atlas of the Mouse Brain, Related to Figure 2A [file mmc2.zip › Data1/âXâëâCâh4.TIF]

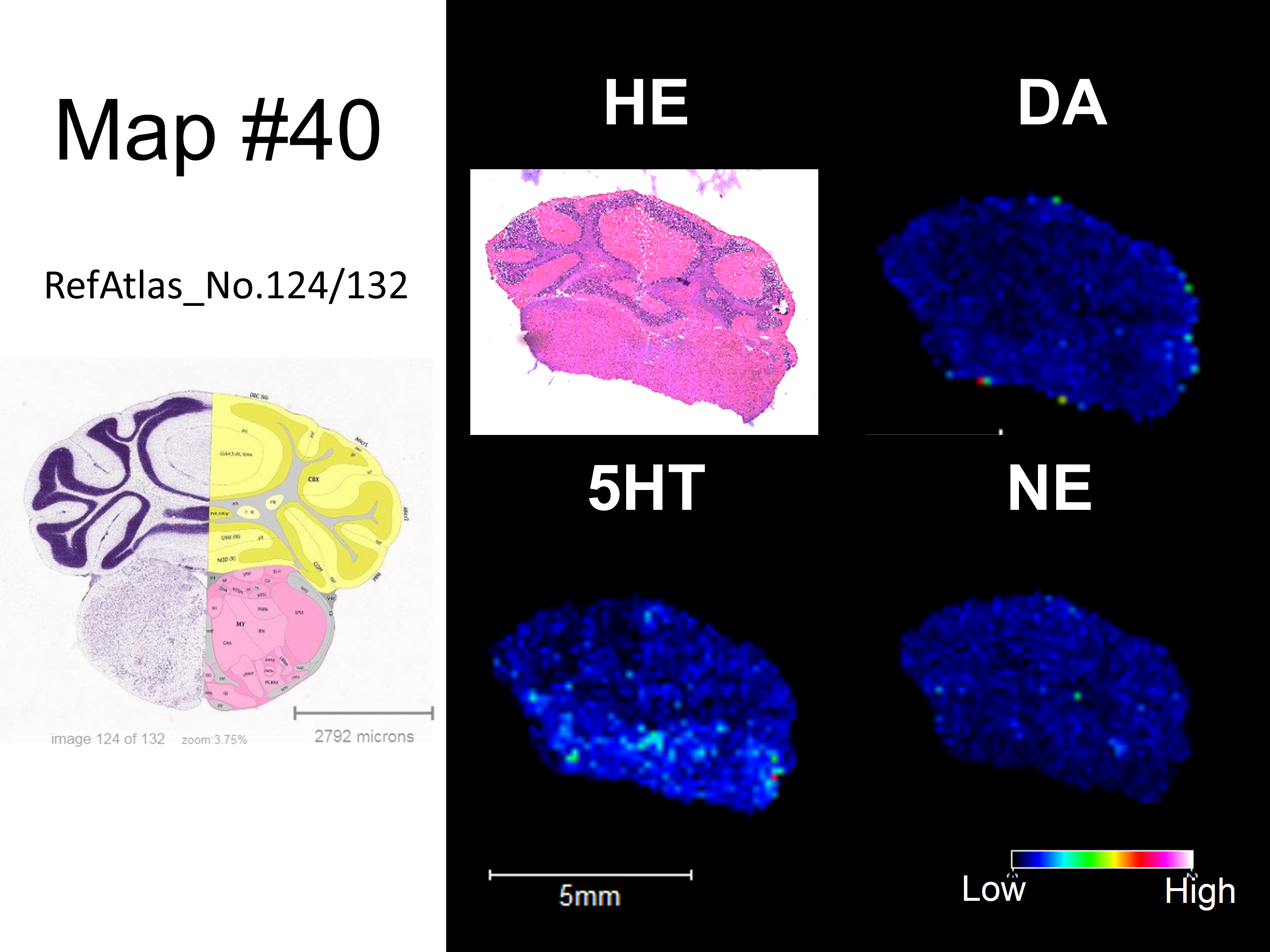

Supplement: Data S1. The Monoamine Atlas of the Mouse Brain, Related to Figure 2A [file mmc2.zip › Data1/âXâëâCâh40.TIF]

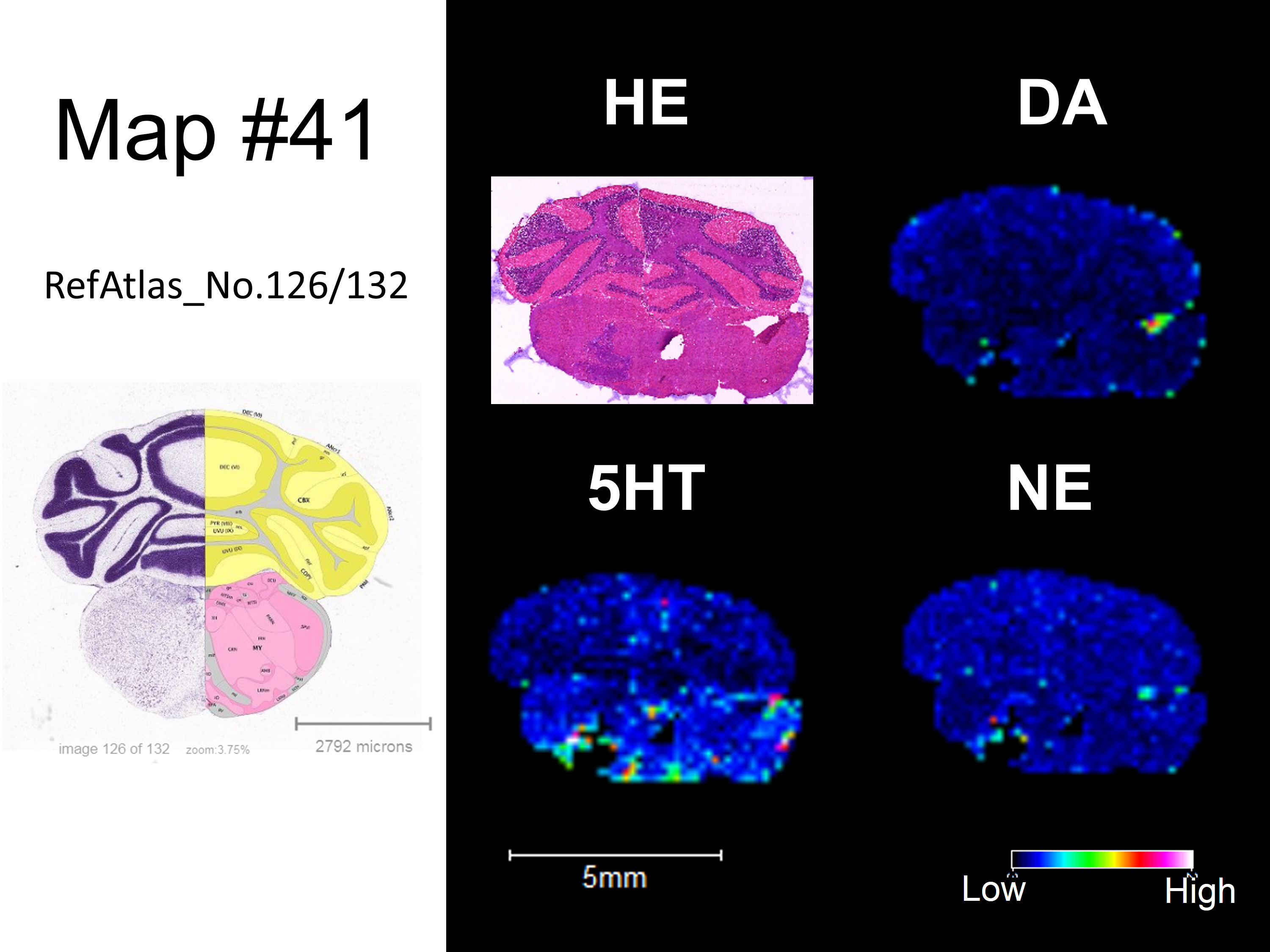

Supplement: Data S1. The Monoamine Atlas of the Mouse Brain, Related to Figure 2A [file mmc2.zip › Data1/âXâëâCâh41.TIF]

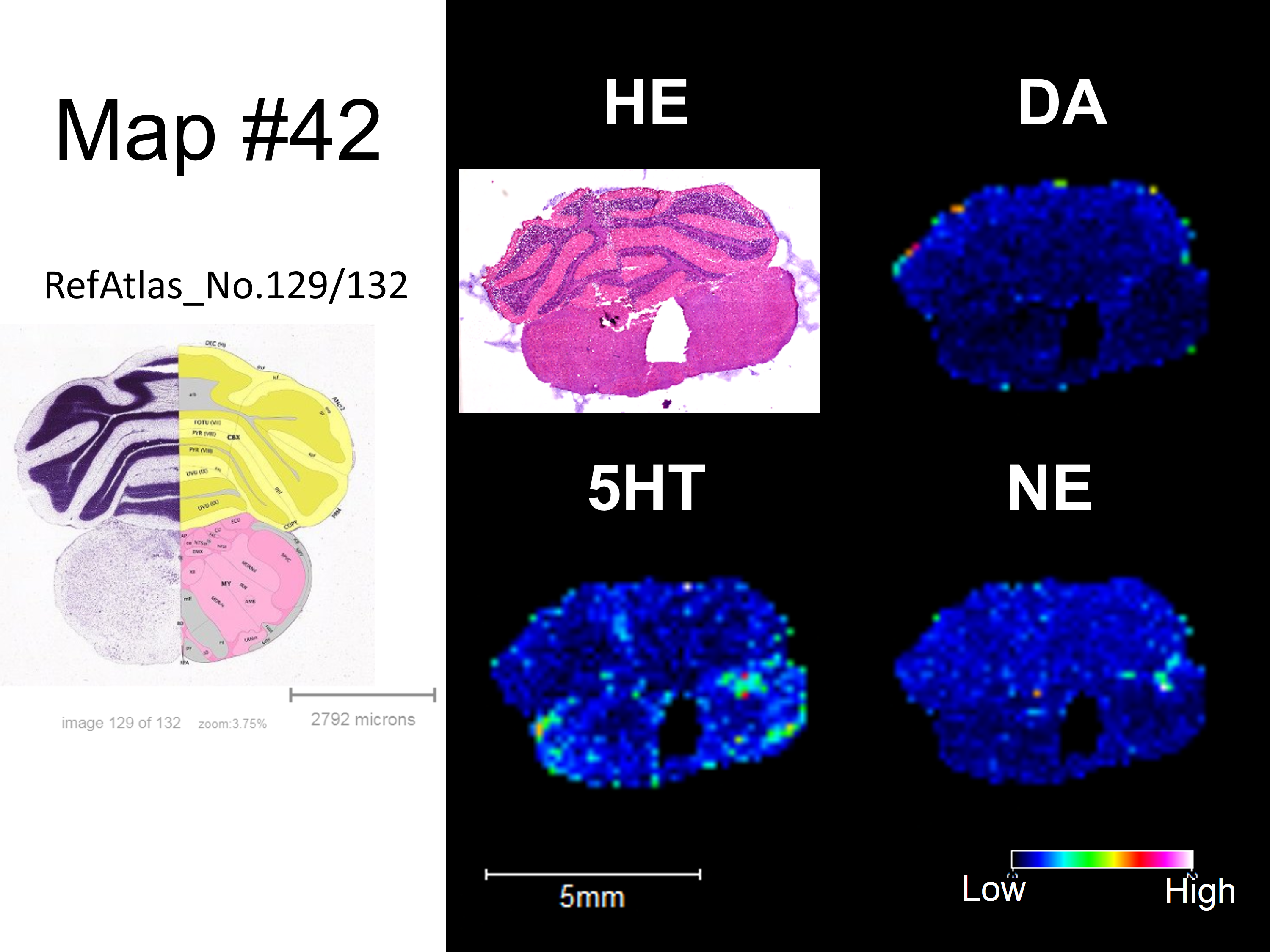

Supplement: Data S1. The Monoamine Atlas of the Mouse Brain, Related to Figure 2A [file mmc2.zip › Data1/âXâëâCâh42.TIF]

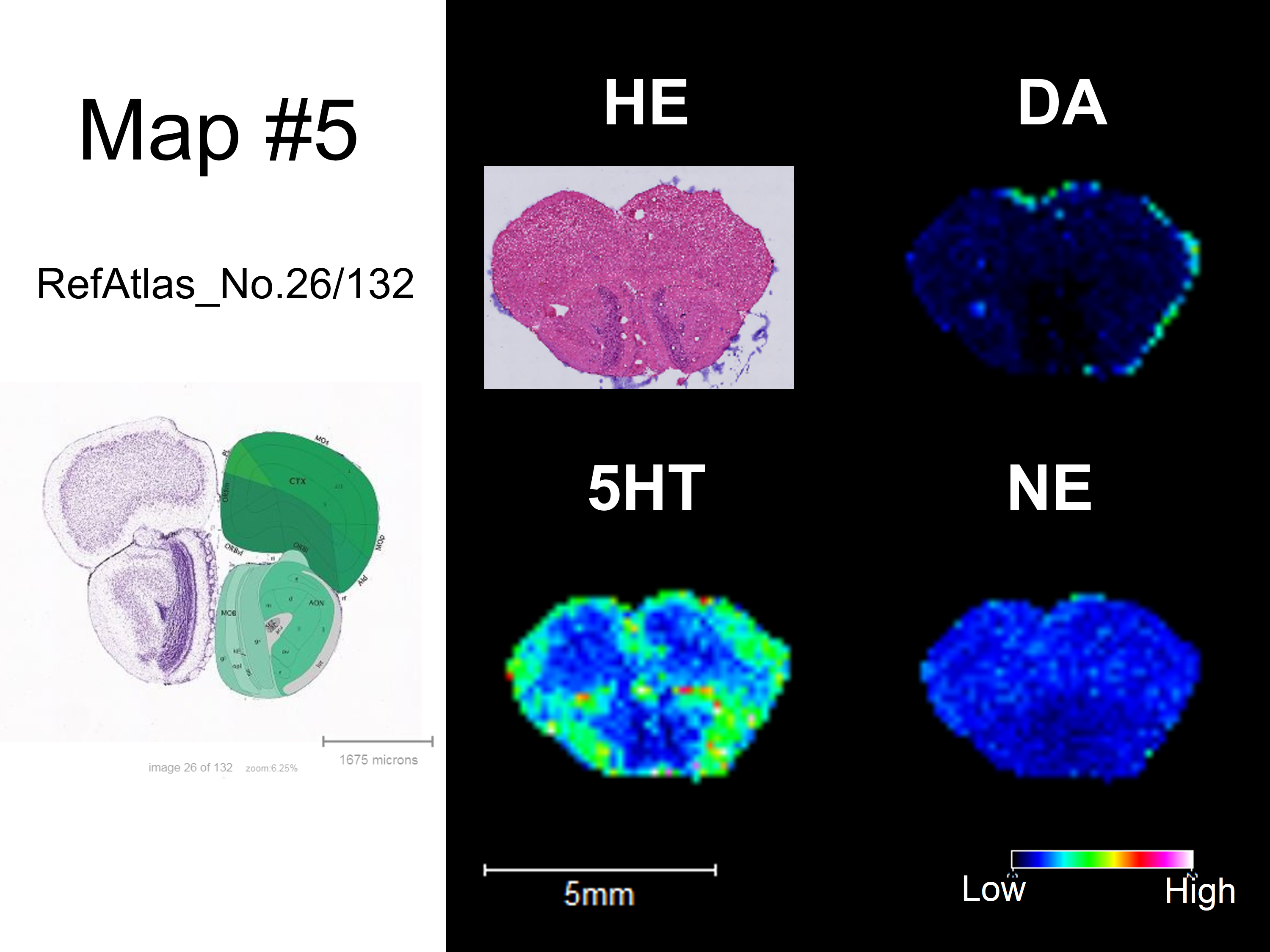

Supplement: Data S1. The Monoamine Atlas of the Mouse Brain, Related to Figure 2A [file mmc2.zip › Data1/âXâëâCâh5.TIF]

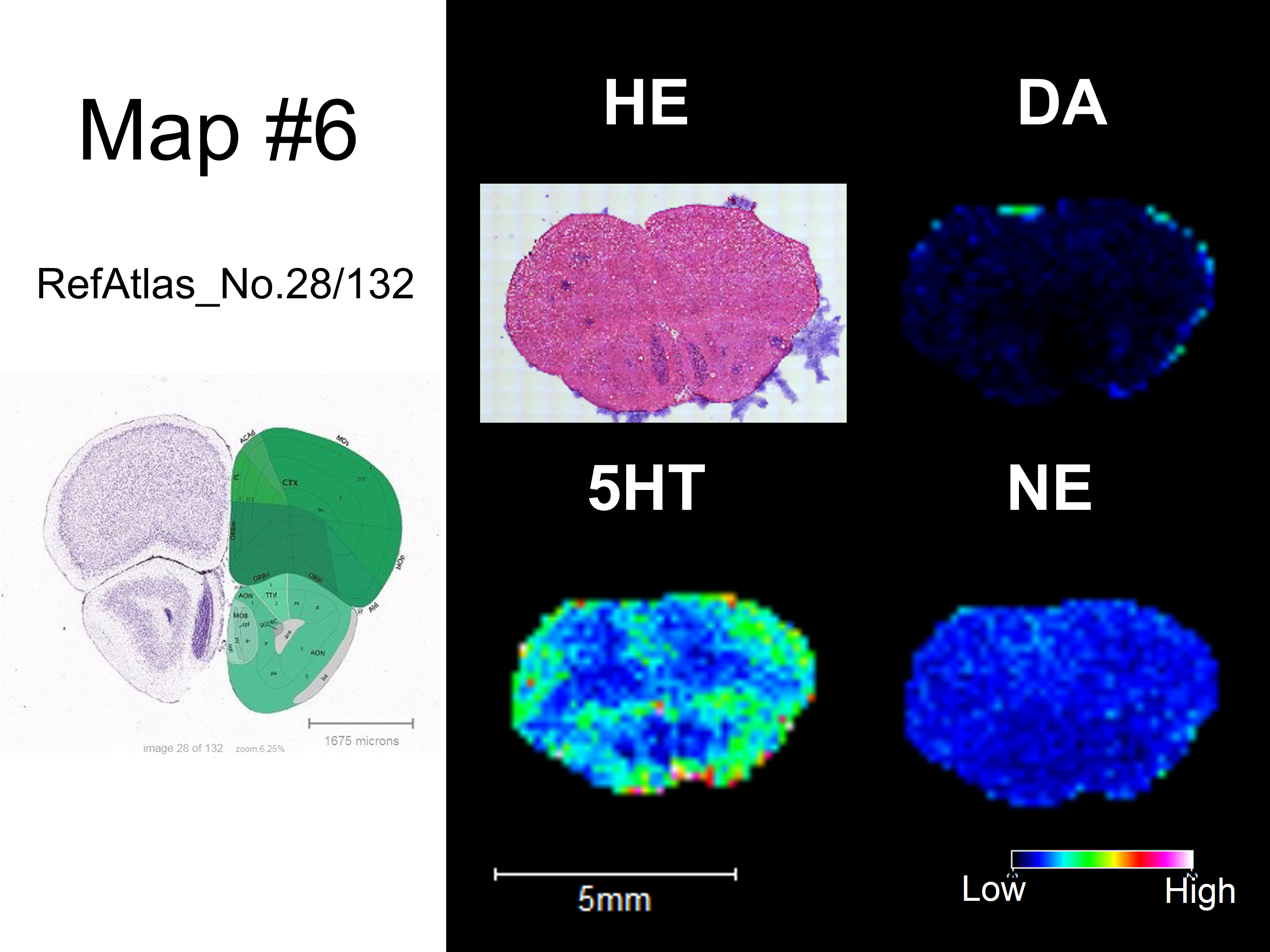

Supplement: Data S1. The Monoamine Atlas of the Mouse Brain, Related to Figure 2A [file mmc2.zip › Data1/âXâëâCâh6.TIF]

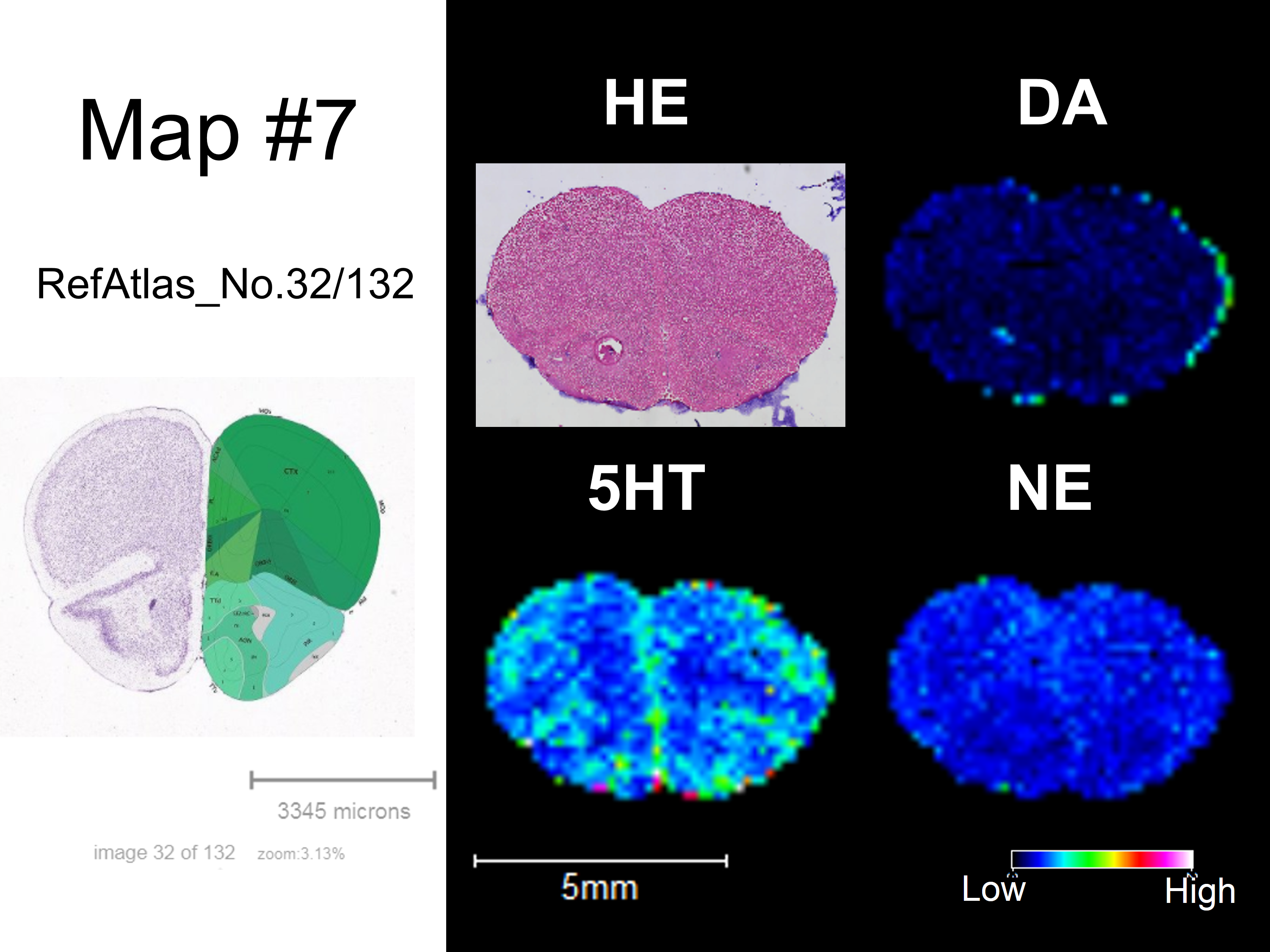

Supplement: Data S1. The Monoamine Atlas of the Mouse Brain, Related to Figure 2A [file mmc2.zip › Data1/âXâëâCâh7.TIF]

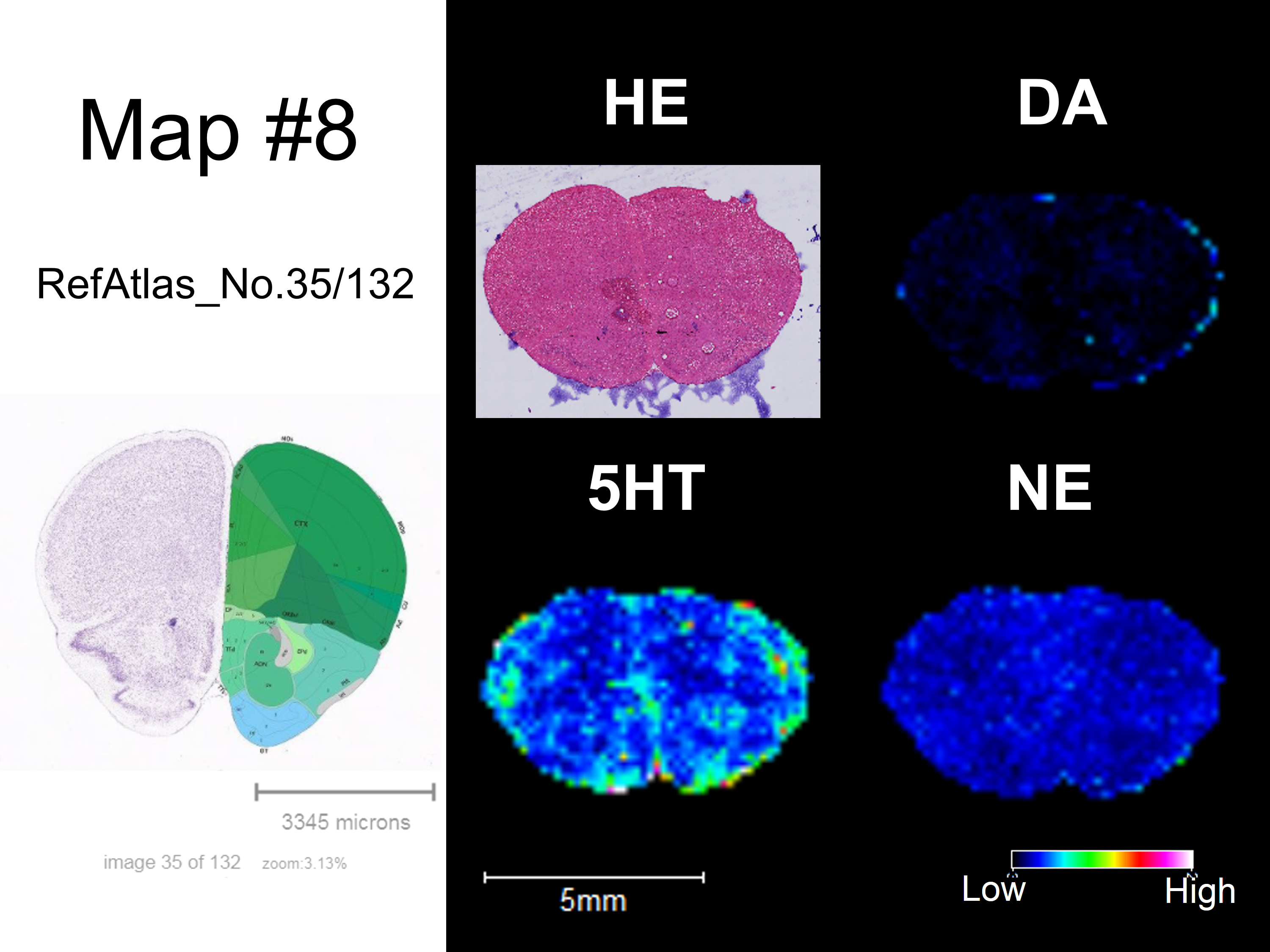

Supplement: Data S1. The Monoamine Atlas of the Mouse Brain, Related to Figure 2A [file mmc2.zip › Data1/âXâëâCâh8.TIF]

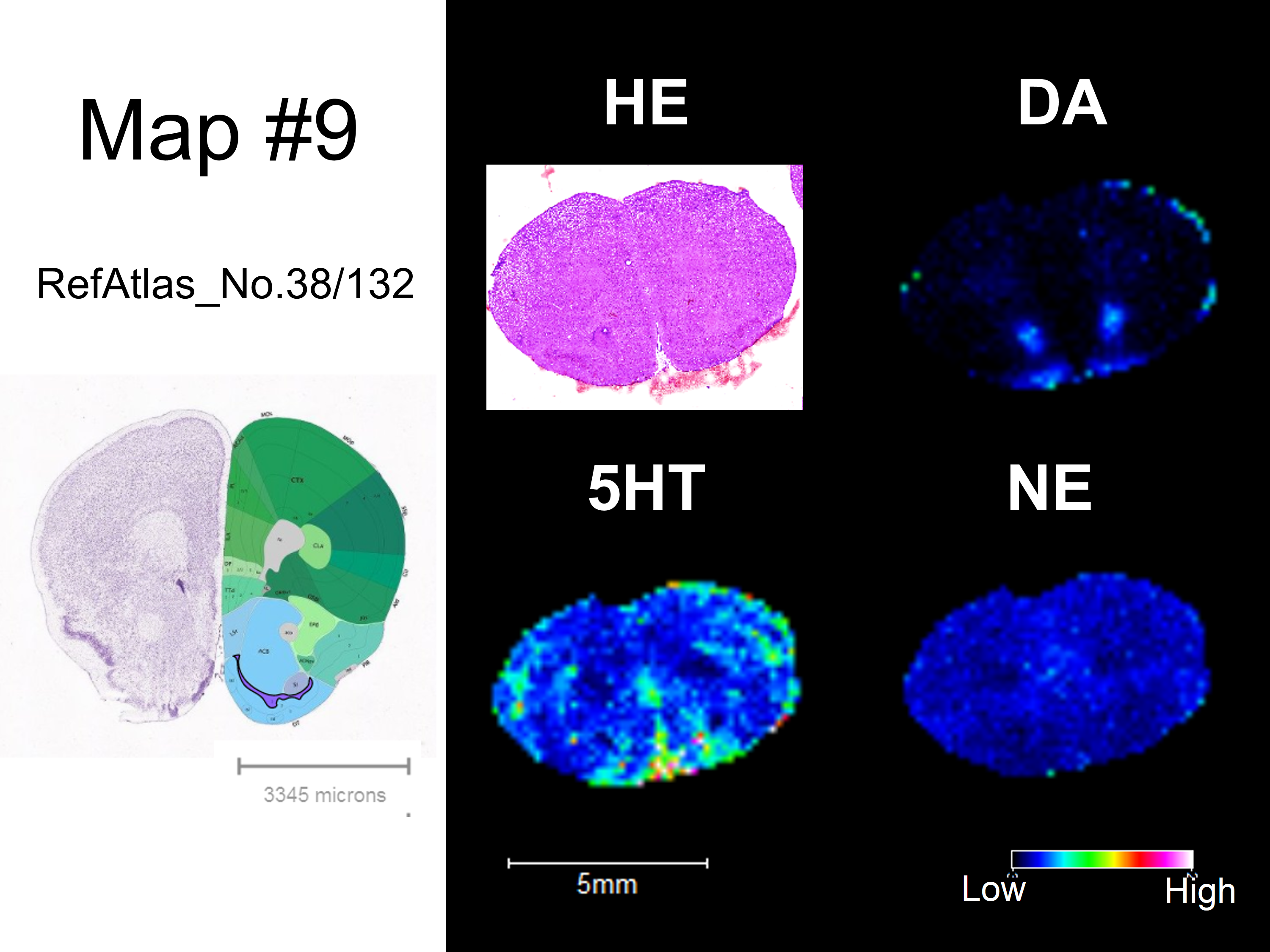

Supplement: Data S1. The Monoamine Atlas of the Mouse Brain, Related to Figure 2A [file mmc2.zip › Data1/âXâëâCâh9.TIF]
